# Dose accuracy evaluation of multi-leaf collimator modeling in single-isocenter multi-target stereotactic radiosurgery

**DOI:** 10.1016/j.phro.2026.101076

**Published:** 2026-09-10

**Authors:** Farnaz Rahim Li, Rex A. Cardan, Joel A. Pogue, Natalie N. Viscariello, Rodney J. Sullivan, John B. Fiveash, Carlos E. Cardenas, Richard A. Popple

**Affiliations:** a Department of Radiation Oncology, The University of Alabama at Birmingham, Birmingham, AL, USA; b Department of Biomedical Engineering, The University of Alabama at Birmingham, Birmingham, AL, USA

**Keywords:** Enhanced leaf model, MLC configuration, Dosimetric leaf gap, Stereotactic radiosurgery

## Abstract

**Background and purpose:**

Accurate modeling of multi-leaf collimators is critical for dose calculation in stereotactic radiosurgery (SRS), where steep dose gradients and small target volumes amplify modeling uncertainties. This study compared enhanced leaf modeling and dosimetric leaf gap approaches using two clinically implemented dose calculation algorithms in single-isocenter SRS plans.

**Materials and methods:**

Sixty clinical volumetric modulated arc therapy (VMAT) single-isocenter SRS plans (30 single-target, SIST; 30 multi-target, SIMT) were selected and measured with a two-dimensional diode array, normalized to a point measurement. Dose was calculated using both enhanced leaf model (ELM) and dosimetric leaf gap (DLG) models and the anisotropic analytical algorithm (AAA) and Acuros XB (AXB) algorithms across several spot-size configurations. Plan quality assurance was assessed using gamma analysis for several dose-distance criteria. Dose differences were further analyzed as functions of target size and distance from isocenter.

**Results:**

Under 2%/1 mm gamma criteria, AXB-DLG having spot size 0.5 × 0.5 mm^2^ showed the highest median and mean gamma pass rate (fraction of points having gamma >1), 98.7% and 97.4% respectively, while AXB-ELM having spot-size 0.5 × 0.7 mm^2^ had the lowest, 98% and 96.3%, respectively. SIST and SIMT plans showed no clinically significant differences. AAA-ELM (spot size: 0 × 0 mm^2^) achieved the highest median and mean gamma values, while AXB-DLG remained robust across plan types. Dose calculations were accurate for both configurations, with minimal mean differences (0.0%, range − 2.7% to +1.8%) reported.

**Conclusions:**

DLG showed slightly better VMAT SRS agreement than ELM, but differences were clinically negligible. ELM's model-based MLC configuration removes user-specific DLG variability, potentially improving dose-calculation precision and consistency across institutions.

## Highlights

• Multileaf collimator modeling impacts dose accuracy in stereotactic radiosurgery.
• Mean dose differences were 0.0% (range: −2.7% to +1.8%).
• Gamma pass rates reached 98.7% using 2%/1 mm criteria.
• Enhanced leaf model achieved clinical agreement without parameter tuning.
• Enhanced leaf model reduced dependence on user-selected configuration.

## Graphical abstract

**Figure:**
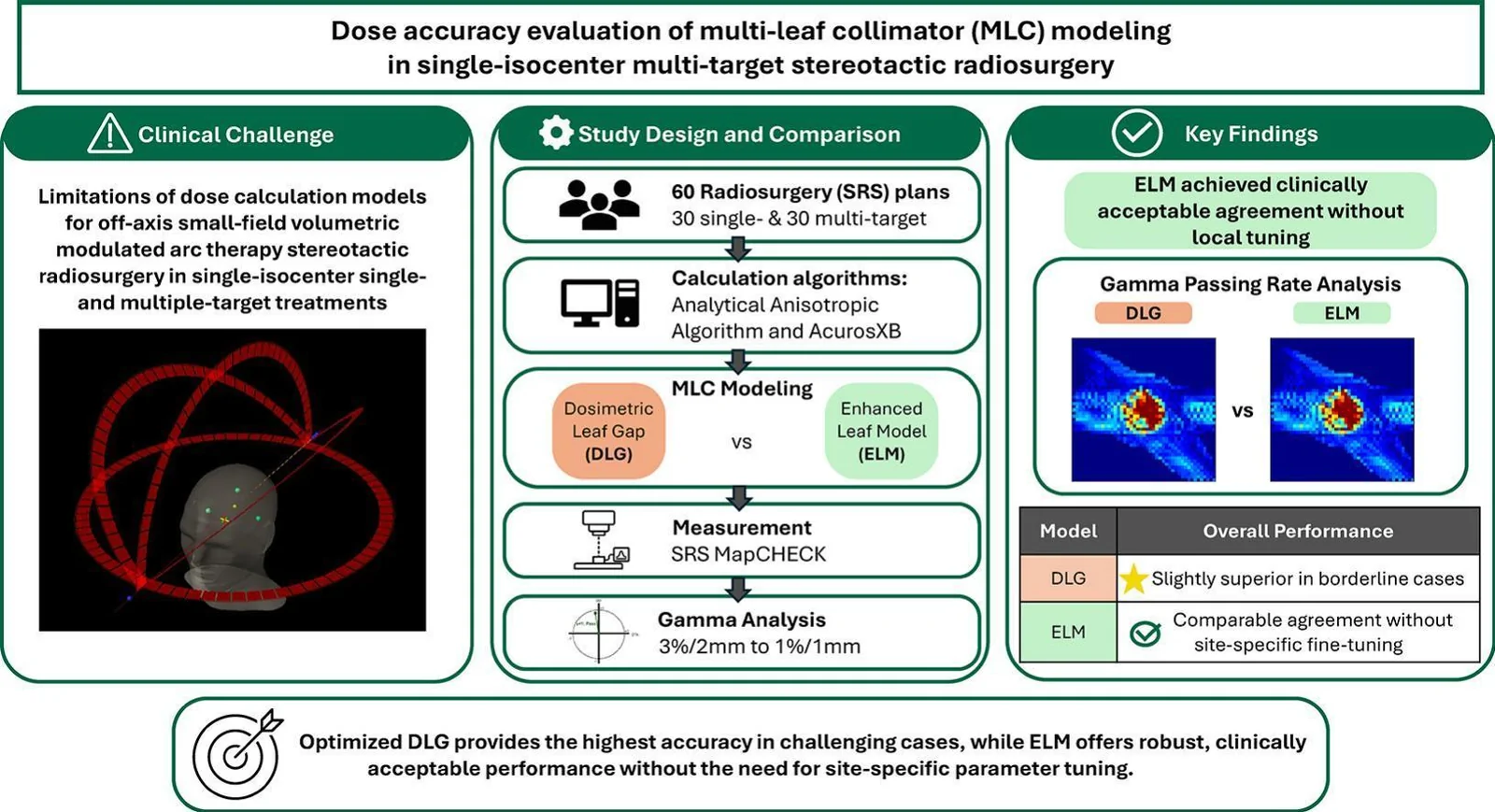

## 1 Introduction

Accurate dose calculation is critical for radiotherapy treatment planning. Available dose calculation algorithms often use a simple model of the multileaf collimator (MLC), with leaves assumed to have a flat end aligned with beam divergence and uniform attenuation. The effect of the rounded leaf ends was approximated by a fixed increment to the leaf opening, referred to as the dosimetric leaf gap (DLG) [1]. While this approach captures first-order behavior, DLG-based modeling can be challenged by the small fields, steep gradients, and dynamic leaf motion common in stereotactic radiosurgery (SRS), and multiple reports have shown clinically relevant discrepancies between calculated and measured doses that are partially attributable to MLC modeling limitations [2], [3], [4], [5], [6]. Several studies have examined strategies to optimize DLG-based modeling [7], [8], [9], [10], [11], however, optimizing the DLG remains labor intensive and prone to user error.

Recent upgrades to a standard treatment planning system (TPS) have enabled a physics-based representation that explicitly models rounded leaf tips and drive screw cutouts and incorporates a leaf-design–based attenuation model, thereby reducing reliance on user-tuned parameters such as DLG [12], [13]. Direct leaf modeling is therefore expected to improve dosimetric agreement, particularly for small fields and off-axis targets in complex volumetric modulated arc therapy (VMAT) deliveries.

Several studies have evaluated direct leaf modeling performance in stereotactic radiosurgery (SRS) contexts and generally report improved agreement relative to DLG-based calculations [13], [14], [15]; however, important clinical questions remain unresolved. Prior work has often been limited by small cohorts, heterogeneous validation strategies, and a focus on convolution-superposition algorithm-based calculations, and it remains unclear whether the reported leaf modeling advantages persist when the baseline DLG model is carefully optimized for SRS. In addition, the practical sensitivity of leaf modeling performance to commissioning choices (e.g., effective spot-size settings) has not been clearly established, despite being directly relevant to clinical adoption.

The objective of this study was to quantify whether direct leaf modeling improved VMAT-SRS patient-specific quality assurance (PSQA) performance relative to a clinically optimized DLG-based model specifically tuned for SRS. Secondary objectives were to evaluate the robustness of leaf modeling across commonly used spot-size configurations and to compare model performance for single-isocenter single-target and single-isocenter multi-target plans, including small and off-axis targets.

## 2 Materials and methods

### 2.1 Patient selection and measurements

Sixty HyperArc VMAT-SRS plans were selected from our institutional clinical database under Institutional Review Board (IRB) approval number IRB-120703005 [16]. The cohort included 30 single-target and 30 multi-target plans, all treated with a single isocenter using a 10 MV flattening filter-free (FFF) beam delivered on an Edge linear accelerator (Varian Medical Systems, Palo Alto, CA) equipped with an HD120 MLC having 2.5 mm central leaf and 5 mm peripheral leaf widths. Target characteristics can be found in Table 1. Each plan incorporated at least one non-coplanar arc, with 20 plans including an arc in the sagittal plane (table angle 90° or 270°). The median modulation factor (total monitor units / prescription dose) of SIST plans was 3.9 MU/cGy, compared to 4.4 MU/cGy for SIMT plans (*p*-value = 0.004, two-sample *t*-test). The monitor units for each plan are given in Supplementary Materials Table S1 and Fig. S1.

**Table 1:** Summary of target characteristics, stratified by single-isocenter single-target (SIST) and single-isocenter multi-target (SIMT) plans.

	SIST (*n* = 30)	SIMT (n = 30)
Number of targets	1	2–25
Target volume (cm^3^)	0.06–24.1	0.007–46.8
Target equivalent diameter (mm)	3.0–35.8	2.4–44.7
Target distance from isocenter (mm)	–	8.1–74.1

Planar dose measurements for comparison with TPS calculations were performed using the standard clinical PSQA workflow using the SRS MapCHECK (SRSMC) diode array (Sun Nuclear Corporation, Melbourne, FL) [17]. The SRSMC consists of 1013 n-type diodes, each with a 0.007 mm^3^ sensitive volume (0.48 mm × 0.48 mm × 0.03 mm), arranged in a 77 × 77 mm^2^ face-centered grid with a 2.47 mm diode spacing. The array was oriented in the coronal plane in a StereoPHAN (Sun Nuclear Corporation, Melbourne, FL), a polymethylmethacrylate (PMMA) phantom comprised of a 15.2 cm diameter cylinder capped with a hemisphere of the same diameter. SRSMC measurements were analyzed using the vendor provided software, which included corrections for pulse repetition rate, diode temperature, and angular dependence, as described in Popple et al. [17]. Based on prior validation, residual uncertainties after correction are generally expected to be within ∼2% for typical delivery conditions. The plans were delivered using the true composite method described in the report of the American Association of Physicists in Medicine Task Group 218 [18]. In particular, the planned table angles were used. Two measurements were performed for SIMT plans by placing the center of the diode array at the centers of the largest and smallest targets, resulting in 60 total measurements for the 30 SIMT plans, and 90 measurements total. Before each measurement session, a dose calibration was done using a 4 cm × 4 cm field. The calibration was dose-to-water (not PMMA). The measurement protocol is further described in Popple et al. [17], which demonstrated that the SRSMC under responds by about 1%. Therefore, the diode array measurements were normalized to a point measurement using a W2 scintillator detector (Standard Imaging, Madison, WI) [19] positioned at the location of the central diode.

### 2.2 Beam modeling and dose calculation

The StereoPHAN was modeled in the Eclipse TPS (Siemens Healthineers, Munich, Germany) using synthetic CT images having 1 mm spacing and an external contour generated to match the phantom dimensions, as described in Popple et al. [17]. The CT values inside the external contour were assigned material type PMMA having mass density 1.19 g/cm^3^ and corresponding relative electron density 1.16.

Dose was re-calculated for the 90 measurement configurations using a 1 mm grid spacing with both Eclipse AcurosXB (AXB) and Analytical Anisotropic Algorithm (AAA). Several combinations of software version, MLC model, and spot-sizes were investigated and are summarized in Table 2. For AcurosXB v.16, two parameter combinations were evaluated. The first employed a spot-size of 0.5 mm × 0.5 mm with a dosimetric leaf gap (DLG) of 0.51 mm, corresponding to the clinically commissioned beam model used for the VMAT-SRS treatments. For reference, the measured DLG value was 0.49 mm. The second combination used the vendor-recommended spot-size of 1.5 mm × 0 mm with a DLG of 0.91 mm, which was selected to optimize agreement with measurements using the same optimization methodology applied during clinical beam model tuning. For consistency with the diode array calibration and AAA calculations, AcurosXB was configured to report dose-to-water. For direct leaf modeling with the Eclipse enhanced leaf model (ELM), symmetric spot-sizes and the vendor recommended spot-size (0.5 × 0.7 mm^2^) were tested.

**Table 2:** Overview of calculation parameters in Eclipse TPS applied for gamma analysis.

Algorithm	Version	MLC model	Spot-size (X x Y) (mm^2^)
AXB	18	ELM	0 × 0 0.25 × 0.25 0.5 × 0.5 0.5 × 0.7⁎
AXB	16	DLG 0.51 mm DLG 0.91 mm	0.5 × 0.5 1.5 × 0⁎
AAA	18	ELM	0 × 0

⁎ Recommended vendor value.

Gamma analysis was done using software based on the work by Krawczyk and Grzanka [20]. Gamma analysis used global normalization, a 10% dose threshold, and dose/distance criteria of 1%/1 mm, 2%/1 mm, 3%/1 mm, and 3%/2 mm. As this study reviews SRS plan QA, the results are focused on the gamma passing rate (GPR) test with the 2%/1 mm criterion, providing a balance between stringent assessment and clinical relevance. The dose difference between calculation and scintillator measurement were also evaluated.

## 3 Results

Under the most stringent gamma criteria (1%/1 mm), the model having the closest agreement with measurement was AXB-DLG with a spot-size of 0.5 × 0.5 mm^2^, which demonstrated the most robust and stable results across all gamma criteria and plan types, achieving a median of 96.0% and a mean of 92.6% (Table 3 and Fig. 1.a). In contrast, AXB-ELM with a spot-size of 0.5 × 0.7 mm^2^ had the largest disagreement, with a median GPR of 94.9% and a mean of 90.6%, indicating reduced accuracy in tight tolerance conditions.

**Table 3:** Summary of GPR results (mean% ± SD% [minimum-maximum]%) for each configuration model across four gamma criteria (3%/2 mm, 3%/1 mm, 2%/1 mm, and 1%/1 mm) in both single-isocenter single-target (SIST) and single-isocenter multi-target (SIMT) plans.

Model		1%/1 mm	2%/1 mm	3%/1 mm	3%/2 mm
AAA-ELM (0 × 0 mm^2^)		89.5 ± 14.6 [21−100]	96.2 ± 5.6 [65.-100]	98.2 ± 2.6 [85–100]	99.4 ± 1.5 [90–100]
	**SIST**	94.9 ± 3.9 [82–100]	96.1 ± 3.4 [83–100]	97.3 ± 3.1 [85–100]	98.7 ± 2.2 [90–100]
	**SIMT**	86.8 ± 17.0 [21–99]	96.2 ± 6.5 [65–100]	98.7 ± 2.2 [88–100]	99.8 ± 0.7 [96–100]
AXB-DLG (0.5 × 0.5 mm^2^) DLG 0.51 mm		92.6 ± 9.1 [56–100]	97.4 ± 3.1 [83–100]	98.4 ± 2.3 [85–100]	99.3 ± 1.6 [89–100]
	**SIST**	94.0 ± 4.7 [81–100]	95.7 ± 3.7 [83–100]	96.8 ± 3.2 [85–100]	98.2 ± 2.4 [89–100]
	**SIMT**	91.9 ± 10.6 [56–100]	98.2 ± 2.5 [85–100]	99.2 ± 1.1 [93–100]	99.9 ± 0.4 [97–100]
AXB-DLG (1.5 × 0 mm^2^) DLG 0.91 mm		73.9 ± 27.7 [5–100]	87.2 ± 18.9 [17–100]	95.4 ± 7.9 [56–100]	99.1 ± 2.7 [83–100]
	**SIST**	95.7 ± 3.8 [85–100]	97.6 ± 3.0 [87–100]	98.8 ± 1.8 [93–100]	99.6 ± 0.8 [97–100]
	**SIMT**	63.0 ± 28.1 [5–99]	82.0 ± 21.3 [17–100]	93.7 ± 9.2 [56–100]	98.8 ± 3.3 [83–100]
AXB-ELM (0 × 0 mm^2^)		91.2 ± 12.6 [29–100]	96.5 ± 4.8 [73–100]	98.1 ± 2.8 [84–100]	99.3 ± 1.8 [87–100]
	**SIST**	94.4 ± 4.7 [80–99]	95.8 ± 3.8 [82–99]	96.9 ± 3.3 [84–100]	98.4 ± 2.7 [87–100]
	**SIMT**	89.4 ± 14.9 [29–100]	96.9 ± 5.2 [73–100]	98.7 ± 2.3 [87–100]	99.8 ± 0.8 [94–100]
AXB-ELM (0.25 × 0.25 mm^2^)		91.0 ± 12.7 [29–100]	96.5 ± 4.9 [72–100]	98.1 ± 2.8 [84–100]	99.3 ± 1.8 [87–100]
	**SIST**	94.3 ± 4.6 [80–99]	95.7 ± 3.8 [82–100]	96.9 ± 3.4 [84–100]	98.3 ± 2.7 [87–100]
	**SIMT**	89.4 ± 15.0 [29–100]	96.9 ± 5.3 [72–100]	98.7 ± 2.3 [86–100]	99.7 ± 0.9 [93–100]
AXB-ELM (0.5 × 0.5 mm^2^)		90.8 ± 12.9 [28–100]	96.4 ± 5.0 [71–100]	98.0 ± 3.0 [83–100]	99.2 ± 1.9 [86–100]
	**SIST**	93.7 ± 4.8 [80–100]	95.2 ± 3.9 [82–100]	96.5 ± 3.5 [83–100]	98.1 ± 2.8 [86–100]
	**SIMT**	89.4 ± 15.2 [28–100]	97.0 ± 5.4 [71–100]	98.8 ± 2.3 [86–100]	99.7 ± 1.0 [93–100]
AXB-ELM (0.5 × 0.7 mm^2^)		90.6 ± 12.9 [27–100]	96.3 ± 5.1 [70–100]	97.9 ± 3.0 [83–100]	99.1 ± 2.0 [86–100]
	**SIST**	93.3 ± 4.9 [80–99]	95.0 ± 3.9 [82–99]	96.3 ± 3.5 [83–100]	97.9 ± 2.9 [86–100]
	**SIMT**	89.3 ± 15.3 [27–100]	96.9 ± 5.6 [70–100]	98.8 ± 2.3 [86–100]	99.7 ± 1.1 [92–100]

**Fig. 1:**
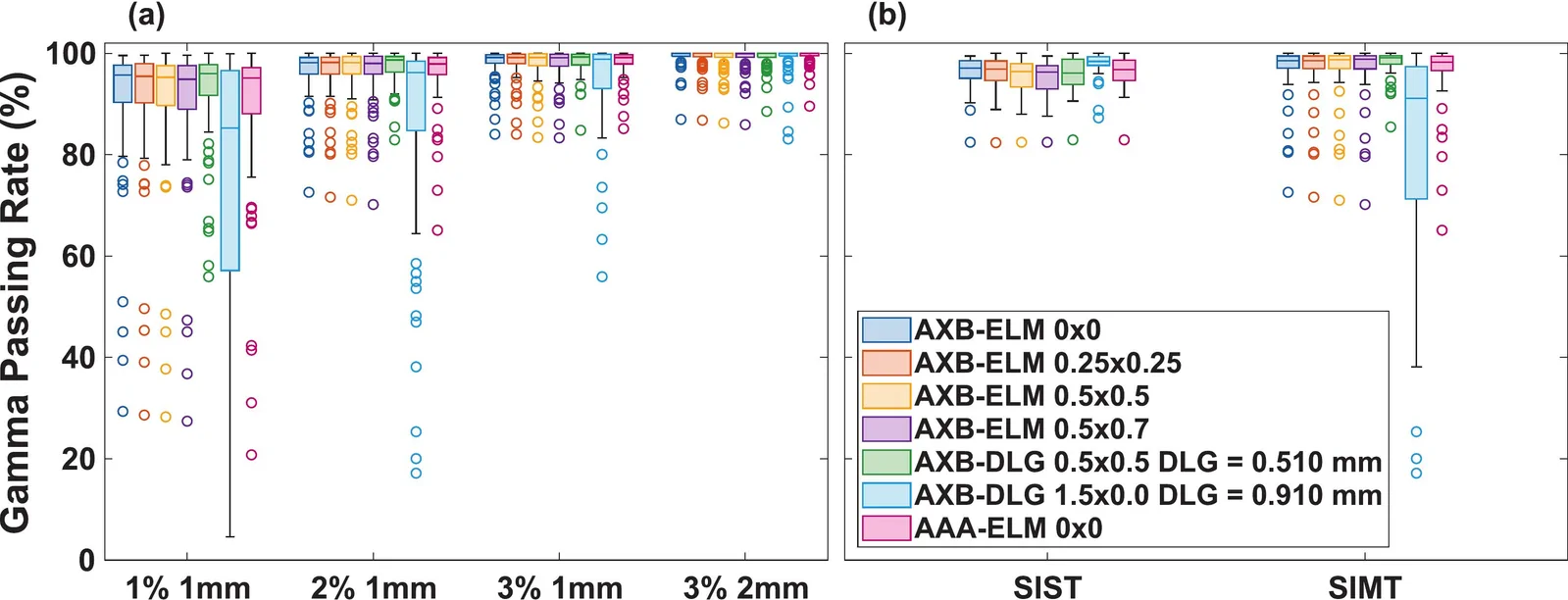
(a) Comparison of gamma passing rates (GPRs, %) across dose calculation models and spot-size settings under four gamma criteria: 1%/1 mm, 2%/1 mm, 3%/1 mm, and 3%/2 mm. Each boxplot represents the distribution of GPR values for 90 targets. Evaluated configurations include AXB-ELM, AXB-DLG, and AAA-ELM with various spot sizes. (b) GPRs (%) stratified by plan type and dose calculation configuration using the 2%/1 mm criterion. Plan types include single-isocenter/single-target (SIST) and single-isocenter/multiple-target (SIMT) plans. Colors denote the AAA-ELM, AXB-DLG, and AXB-ELM configurations with spot-size settings of 0 × 0, 0.25 × 0.25, 0.5 × 0.5, 0.5 × 0.7, and 1.5 × 0 mm^2^. Boxes indicate the interquartile range (Q1–Q3), with the horizontal line representing the median; whiskers extend to 1.5 times the interquartile range, and circles indicate individual outliers.

At the 2%/1 mm criterion, differences in median GPR among configurations were small. For single-isocenter multi-target (SIMT) plans, the AXB-DLG configuration with a 0.5 × 0.5 mm^2^ spot-size achieved the highest median GPR (99.1%). In contrast, for single-isocenter single-target (SIST) plans, the highest median GPR (98.4%) was achieved with the AXB-DLG configuration using a 1.5 × 0.0 mm^2^ spot-size. Whereas the AXB-ELM configurations produced slightly lower median GPRs (Fig. 1b), with values of 96.4% for SIST and 98.8% for SIMT. SIST plans demonstrated tighter GPR distributions and less variability across configurations than SIMT plans, with a mean GPR of 95.6% ± 3.7% [82–100]%. In contrast, SIMT plans achieved a higher pooled mean of 97.0% ± 5.2% [65–100]% but exhibited wider distributions and more pronounced low-value outliers.

For AXB-ELM, GPRs obtained with the 0 × 0 and 0.5 × 0.7 mm^2^ spot-size configurations were strongly correlated at the 2%/1 mm criterion (R^2^ = 0.9822; Fig. 2a). This strong association indicated that variations in GPR among plans were similar for the two evaluated spot-size settings. GPRs obtained with the DLG and ELM configurations were moderately to strongly correlated at the same criterion (R^2^ = 0.71; Fig. 2b).

**Fig. 2:**
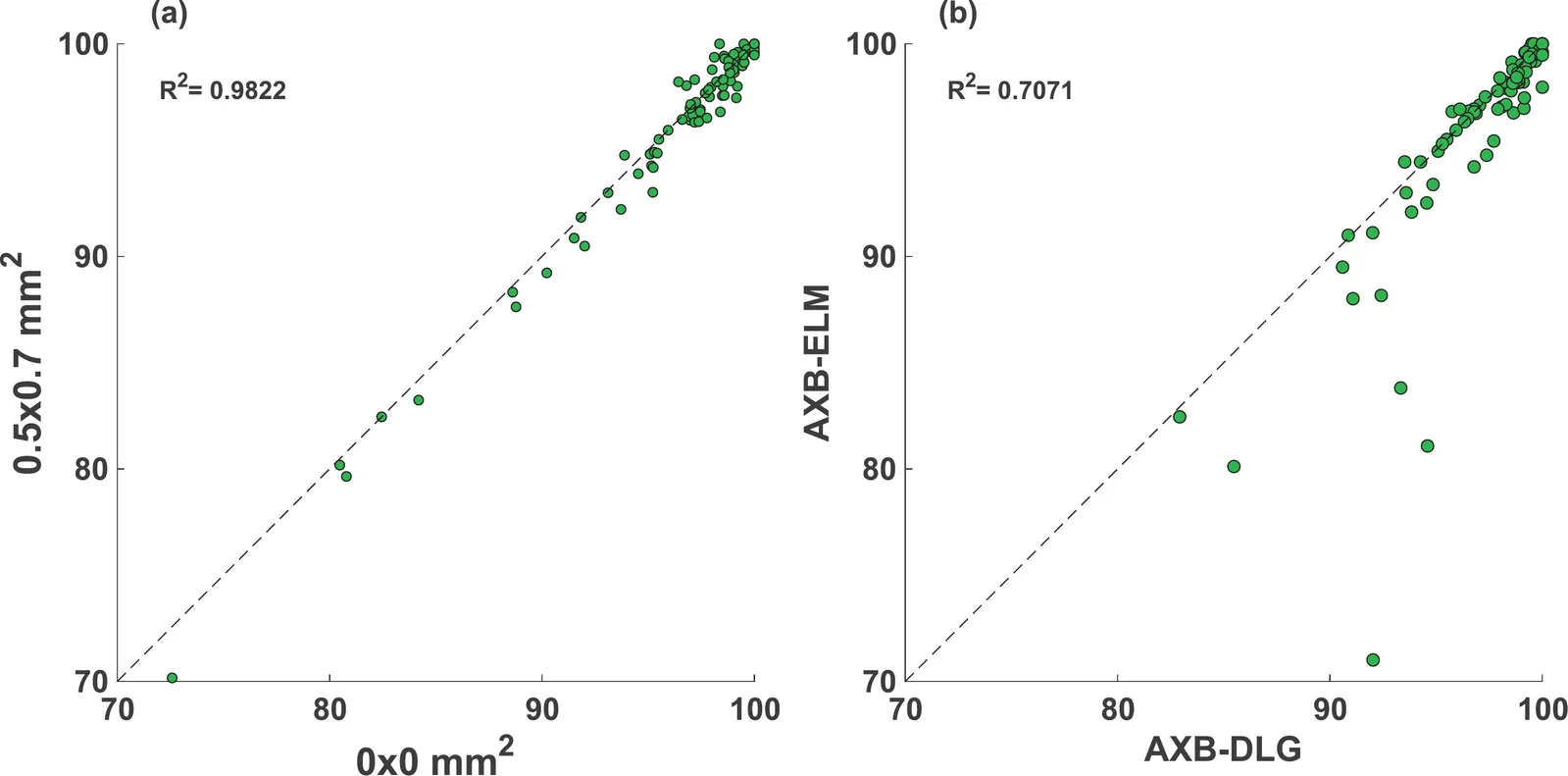
(a) Scatter plot comparing GPR between AXB-ELM Spot Size 0 × 0 mm^2^ and 0.5 × 0.7 mm^2^ configurations under 2%/1 mm gamma criteria. The strong correlation (R^2^ = 0.98) and tight clustering around the identity line indicate high agreement between the two spot sizes across all evaluated plans. (b) Comparison of GPR between AXB-DLG V16 and AXB-ELM MLC configurations for spot size 0.5 × 0.5 mm^2^ using the 2%/1 mm criterion. Each point represents gamma value in each calculation model, with the dashed line indicating the identity (perfect agreement) line.

Center-diode dose differences demonstrated similar patterns for the ELM and DLG configurations across target volumes and distances from the isocenter. Several representative configurations are shown in Fig. 3 and plots for other configuration combinations are provided in the Supplementary Materials Figs. S2a, S2b, and S2c. For targets located within 15 mm of the isocenter, the mean dose difference between calculated and measured doses was 2.1% and 2.8% for the AXB-DLG and AXB-ELM MLC configurations, respectively. For small targets (<1 cm^3^) located more than 15 mm from the isocenter, the mean dose difference was −1.4% for the AXB-DLG V16 configuration and − 0.8% for the AXB-ELM MLC configuration. For larger targets (>1 cm^3^) located more than 15 mm from the isocenter, measured doses again tended to exceed calculated doses, with mean dose differences of 0.4% and 1.7% for the AXB-DLG V16 and AXB-ELM MLC configurations, respectively. Across all measurements, the mean calculation-minus-measurement dose differences were − 0.7% for both ELM and DLG. The mean paired difference between ELM and DLG was 0.0%, with individual differences ranging from −2.7% to +1.8%.

**Fig. 3:**
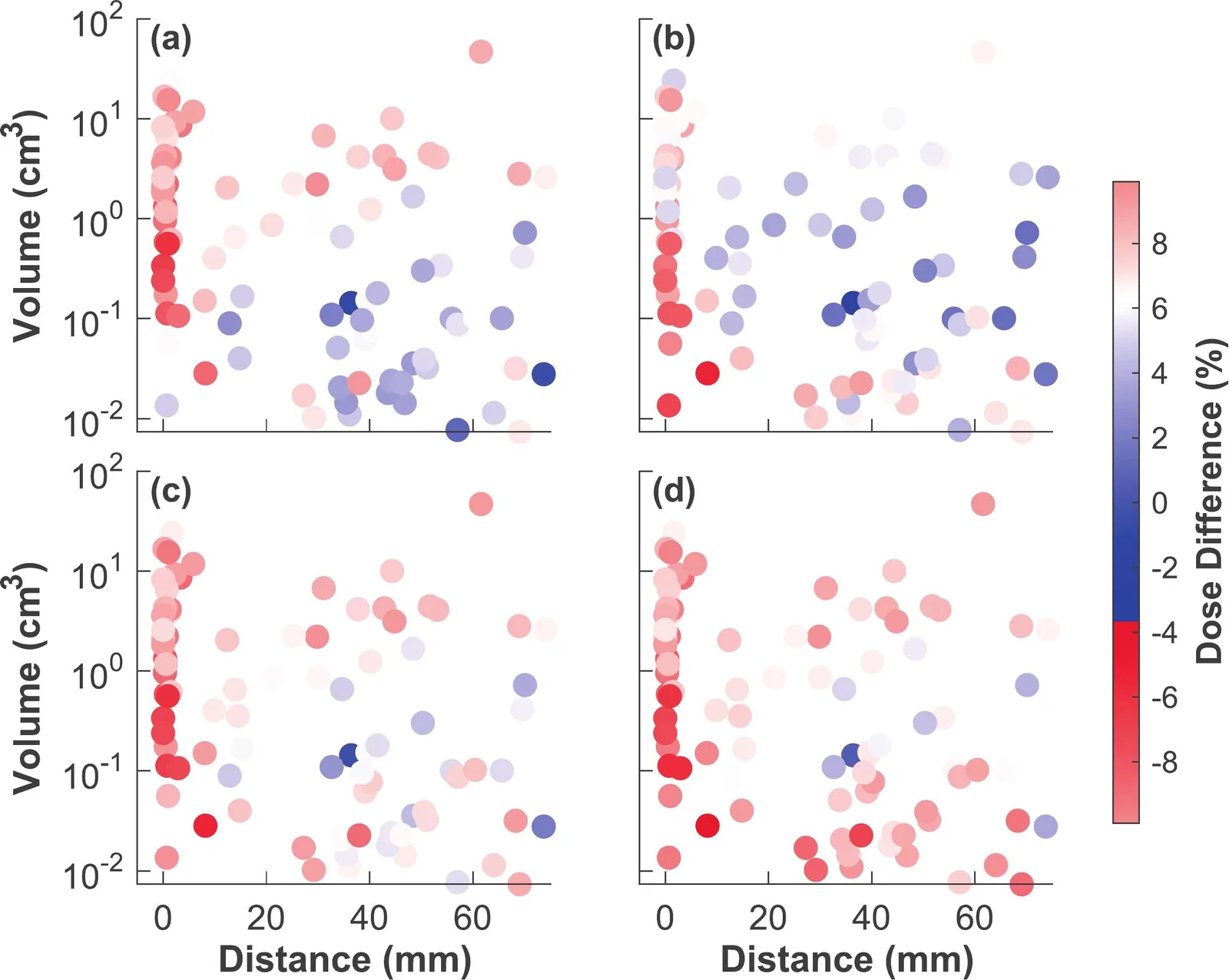
Relative central-dose difference ([measured – calculated] / measured) for each measurement configuration, shown as a function of target size and distance from isocenter for following configurations: (a) AXB-DLG with a 0.5 × 0.5 mm^2^ spot size and DLG = 0.510 mm; (b) AXB-DLG with a 1.5 × 0 mm^2^ spot size and DLG = 0.910 mm; (c) AXB-ELM with a 0.5 × 0.5 mm^2^ spot size; and (d) AXB-ELM with a 0.5 × 0.7 mm^2^ spot size. Color indicates the magnitude and direction of the measurement–calculation dose difference, rather than the paired ELM–DLG difference.

## 4 Discussion

This study evaluated whether enhanced MLC modeling improves dose calculation accuracy relative to a clinically optimized DLG model for single-isocenter VMAT-SRS treatments. Overall, enhanced MLC modeling did not demonstrate a consistent dosimetric advantage over a clinically optimized DLG model for single-isocenter VMAT-SRS treatments. The AXB-DLG model with a 0.5 × 0.5 mm^2^ spot-size provided the highest and most stable gamma passing rates across both SIST and SIMT plans, although differences among configurations were generally small at the clinically emphasized 2%/1 mm criterion. ELM performance was largely insensitive to the evaluated spot-size settings, and both models demonstrated nearly identical agreement with point-dose measurements. These findings indicate that a carefully optimized DLG model remains highly accurate for VMAT-SRS and that adoption of ELM may not independently resolve the dosimetric variability observed in complex SIMT treatments.

As anticipated, tightening the gamma criteria from 3%/2 mm to 1%/1 mm resulted in progressively lower GPRs across all configurations, consistent with the expected sensitivity of gamma analysis to stricter tolerances. When evaluated under the strictest criterion (1%/1 mm), however, the spread in GPRs across configurations became more pronounced, revealing the influence of specific algorithm–MLC–spot-size combinations on dose calculation accuracy. Notably, the AXB–DLG model with a spot-size of 0.5 × 0.5 mm^2^ consistently demonstrated superior agreement with measurement, with the highest median (96%) and mean (92.6%) GPRs. Conversely, the lower performance of AXB–ELM with a 0.5 × 0.7 mm^2^ spot-size (mean GPR of 90.6% under 1%/1 mm) highlighted the potential limitations of the ELM configuration and larger spot-sizes in capturing fine resolution dose gradients accurately. This may be due to increased smoothing or under-sampling effects, particularly impactful in the context of steep dose gradients commonly encountered in stereotactic treatments [21].

Importantly, review of the spatial gamma maps (Supplemental Materials Figs. S3-S92) did not reveal a consistent pattern in the location or dosimetric characteristics of failing points across plans or models. The observed discrepancies appeared to be plan-dependent rather than linked to any recurring spatial feature or systematic failure mode. This further supported the interpretation that the differences observed under stricter evaluation criteria are driven primarily by case-specific characteristics and modeling choices rather than a uniform, reproducible spatial bias.

While these results reflect strong overall agreement between calculated and measured doses, the differences observed under stricter evaluation criteria highlight the need for further investigation into how specific modeling choices impact clinical accuracy for single-isocenter SRS.

Compared with SIST plans, SIMT plans demonstrated higher median GPR values across all configurations but also exhibited a wider spread of GPR values. This finding was consistent with studies reporting better dosimetric agreement and lower QA failure rates in simpler stereotactic plans [22], [23], [24], [25]. The analysis across four ELM spot-sizes demonstrated that, within the spot-size range tested, spot-size selection had negligible impact on clinically relevant dosimetric endpoints, suggesting the clinical equivalence of spot-size selection and supporting adoption of a single default configuration without compromising treatment quality or safety. Comparison between ELM and DLG calculations revealed three distinct patterns tied to target volume and spatial location relative to the isocenter (Fig. 3, visualization approach adapted from Erickson et al. [26]), providing clinically valuable insights. In close-proximity targets (<15 mm from isocenter), both ELM and DLG configurations consistently underestimated the delivered dose, a trend seen predominantly in SIST plans. In contrast, for very small and distant targets (<1 cm^3^ & >15 mm from isocenter), primarily present in SIMT setups, both configurations resulted in calculated doses that were generally higher than measured doses. This was consistent with SIMT treatment planning, where maintaining dosimetric precision for small-volume lesions located farther from the isocenter can be more challenging. For large targets (>1 cm^3^), both ELM and DLG again showed a trend toward higher measured doses compared to calculations, regardless of plan type or distance from isocenter. Importantly, the consistency of these trends across both ELM and DLG configurations suggests that while absolute dose differences are small and clinically acceptable, the models shared inherent behavioral tendencies that should be acknowledged during plan evaluation. Understanding these tendencies allows for more informed interpretation of QA metrics.Van Esch et al. demonstrated that ELM modeling improved dose calculation accuracy at off-axis positions, reporting 3%–12% differences between ELM and DLG at 0–12 cm off-axis using a 3 mm dynamic zebra crosswalk [13]. In line with these findings, the clinical plans showed mean dose differences of −0.66% for ELM and − 0.69% for DLG, indicating close agreement in overall dose accuracy. Moktan et al. reported up to 19% reduction in dose agreement for ELM with narrow sweeping gaps at off-axis positions, where central profile discrepancies increased with smaller gap sizes and greater off-axis distances [14]. This trend was consistent with the observed influence of spatial location was consistent with an effect of spatial location on model accuracy. Yang et al. reported better agreement with AAA-ELM at 1%/1 mm for multi-target plans, evaluating three cases with 2, 4, and 8 targets [15]. In comparison, the present study analyzed a broader range of 2 to 25 targets, including 30 clinical SRS plans with target volumes spanning 0.007 to 46.8 cm^3^. Despite the differences in study design, the results were in good agreement with theirs. Fogliata et al. evaluated AXB-ELM spot-size effectiveness for small fields (0.5 × 0.5–4 × 4 cm^2^) and reported a 0.2% ± 0.4% mean dose difference. Similarly, comparison of the two spot-size configurations (0 × 0 mm^2^ vs. 0.5 × 0.7 mm^2^) showed strong agreement (R^2^ = 0.9822) with 95–100% GPR and only minor, clinically insignificant variations [27].

This study evaluated the dose calculation accuracy across several physics variables, including the dose calculation algorithm and key algorithm/modeling parameters (e.g. effective spot-size and, for DLG-based calculations, dosimetric gap leaf) using a substantial number of clinically delivered single-isocenter SRS targets to assess uncertainty of the new MLC configuration. However, other beam modeling parameters that may influence TPS dose agreement were not investigated and represent important limitations of this work. All plans were calculated using a single beam quality, 10 MV FFF, and the reported findings were therefore strictly applicable to the evaluated beam energy. Because effective spot-size and associated small-field dosimetric behavior are beam-quality dependent and may differ for other energies, the configurations evaluated in this study may not translate directly to other beam qualities. The conclusions should therefore be interpreted within the 10 MV FFF conditions investigated, and ELM and DLG performance should be evaluated for additional beam energies. In addition, only two AXB DLG/spot-size combinations were evaluated. Although these combinations provided a limited sensitivity analysis, they did not represent a comprehensive exploration of the parameter space; therefore, definitive conclusions could not be drawn regarding the broader effects of DLG values or DLG determination methods. Another limitation was that the DLG configuration was evaluated only with AXB and not with AAA. Consequently, direct comparisons between ELM and DLG were limited to a single dose calculation algorithm, and the findings may not generalize to AAA-based implementations. Comparisons among modeling configurations were also based primarily on descriptive analyses of GPR and dose-difference metrics rather than formal paired statistical testing. Although the observed differences were generally small and likely of limited clinical significance, the absence of paired statistical analyses limited inference regarding whether differences between configurations were statistically significant. Finally, although the SRS MapCHECK system had been validated against radiochromic film for noncoplanar VMAT-SRS deliveries, systematic absolute-dose differences of approximately 1% had been reported. Accordingly, the PSQA results should be interpreted within this known measurement uncertainty and were most robust for comparing relative agreement among configurations. Future studies should systematically investigate the combined effects of beam energy, DLG measurement strategy, dosimetric field size, and additional beam-modeling parameters to further characterize ELM and DLG performance in stereotactic radiotherapy.

Agreement between measurement and calculation is a combination of dose calculation accuracy, delivery accuracy, and experimental uncertainty [18]. Mechanical factors such as gantry sag, MLC positioning accuracy, beam steering, and imaging-to-treatment isocenter alignment contribute to the delivery accuracy [28], [29]. A robust quality assurance program reduces but does not eliminate these factors and their effect should be considered when interpreting the results reported here [28], [29]. Additionally, the finite spatial resolution of the SRS MapCHECK system (2.47 mm detector spacing) is an additional limitation, particularly under stringent gamma criteria (e.g., 2%/1 and 1%/1 mm) in small-field SRS dosimetry, as it may reduce sensitivity to fine-scale dose differences between configurations [17], [18].

In this large cohort of clinically delivered HyperArc VMAT-SRS plans, AXB-ELM and a clinically optimized AXB-DLG model showed similarly high PSQA performance (GPR > 95%), with DLG modestly outperforming ELM in borderline cases. Absolute dose differences were small (less than 1% on average) and comparable between ELM and DLG (mean paired difference of −0.03%), with residual outliers primarily observed in SIMT cases, indicating that ELM does not fully resolve the known single-isocenter multi-target off-axis agreement challenge. Notably, the DLG model evaluated here was fine-tuned using SIMT/SIST SRS measurements, which may not reflect typical commissioning practices. Despite slightly better performance with a carefully tuned DLG model, ELM remains attractive for clinical adoption because it achieves clinically acceptable agreement without site-specific fine-tuning.

## CRediT authorship contribution statement

**Farnaz Rahim Li:** Writing – review & editing, Writing – original draft, Visualization, Methodology, Formal analysis, Conceptualization. **Rex A. Cardan:** Writing – review & editing, Software. **Joel A. Pogue:** Writing – review & editing, Formal analysis, Conceptualization. **Natalie N. Viscariello:** Writing – review & editing, Conceptualization. **Rodney J. Sullivan:** Writing – review & editing, Data curation. **John B. Fiveash:** Writing – review & editing, Resources, Funding acquisition. **Carlos E. Cardenas:** Writing – review & editing, Writing – original draft, Supervision, Resources, Project administration, Methodology, Funding acquisition, Formal analysis, Conceptualization. **Richard A. Popple:** Writing – review & editing, Writing – original draft, Visualization, Validation, Supervision, Software, Resources, Project administration, Methodology, Investigation, Funding acquisition, Formal analysis, Data curation, Conceptualization.

## Declaration of competing interest

The authors declare that they have no known competing financial interests or personal relationships that could have appeared to influence the work reported in this paper.
