# Supplementary material for: Dose accuracy evaluation of multi-leaf collimator modeling in single-isocenter multi-target stereotactic radiosurgery
Source: Phys Imaging Radiat Oncol. 2026 Sep 10;41:101076. doi: 10.1016/j.phro.2026.101076 (PMC13634057; doi:10.1016/j.phro.2026.101076)

Table S1. Plan characteristics

Plan	# targets	MU	Dose (cGy)	Modulation factor (MU/cGy)
1	2	7451.0	2000	3.73
2	4	2225.7	700	3.18
3	1	9153.2	2000	4.58
4	1	6901.9	2000	3.45
5	5	8805.2	2000	4.40
6	1	2007.2	600	3.35
7	9	11754.4	2000	5.88
8	1	1520.1	500	3.04
9	9	3214.8	700	4.59
10	1	7297.7	1800	4.05
11	1	1610.4	500	3.22
12	9	12410.8	2000	6.21
13	1	1743.4	500	3.49
14	7	3526.4	600	5.88
15	8	2795.2	500	5.59
16	2	5893.2	2000	2.95
17	1	3481.2	1200	2.90
18	1	8084.7	1750	4.62
19	1	1277.3	500	2.55
20	1	7561.7	1800	4.20
21	6	8891.2	2000	4.45
22	6	10893.3	2000	5.45
23	1	7879.2	2000	3.94
24	10	12921.1	1800	7.18
25	9	3918.6	600	6.53
26	1	1821.5	600	3.04
27	4	2140.3	600	3.57
28	1	7044.5	2000	3.52
29	7	9624.1	2000	4.81
30	2	6910.4	1800	3.84
31	2	8766.1	2000	4.38
32	1	8486.7	2000	4.24
33	1	1990.4	500	3.98
34	2	6261.3	2000	3.13
35	7	10195.1	2000	5.10
36	2	8445.8	2000	4.22
37	1	9649.5	2000	4.82
38	3	6692.4	2000	3.35
39	4	1942.7	500	3.89
40	17	13582.0	1800	7.55
41	1	3492.5	1200	2.91

Plan	# targets	MU	Dose (cGy)	Modulation factor (MU/cGy)
42	1	7732.9	2000	3.87
43	1	8238.9	1750	4.71
44	1	6144.2	1200	5.12
45	2	6211.3	2000	3.11
46	1	7783.9	2000	3.89
47	2	2008.0	600	3.35
48	1	2082.2	500	4.16
49	1	7796.3	1750	4.46
50	4	1935.6	600	3.23
51	4	2480.3	600	4.13
52	1	4517.9	1200	3.76
53	7	2844.9	600	4.74
54	2	7074.5	2000	3.54
55	1	6961.4	1800	3.87
56	1	5977.3	1200	4.98
57	1	1742.6	500	3.49
58	1	5503.3	1750	3.14
59	1	1588.3	500	3.18
60	25	4626.8	600	7.71

Figure S1. Histogram of modulation factors

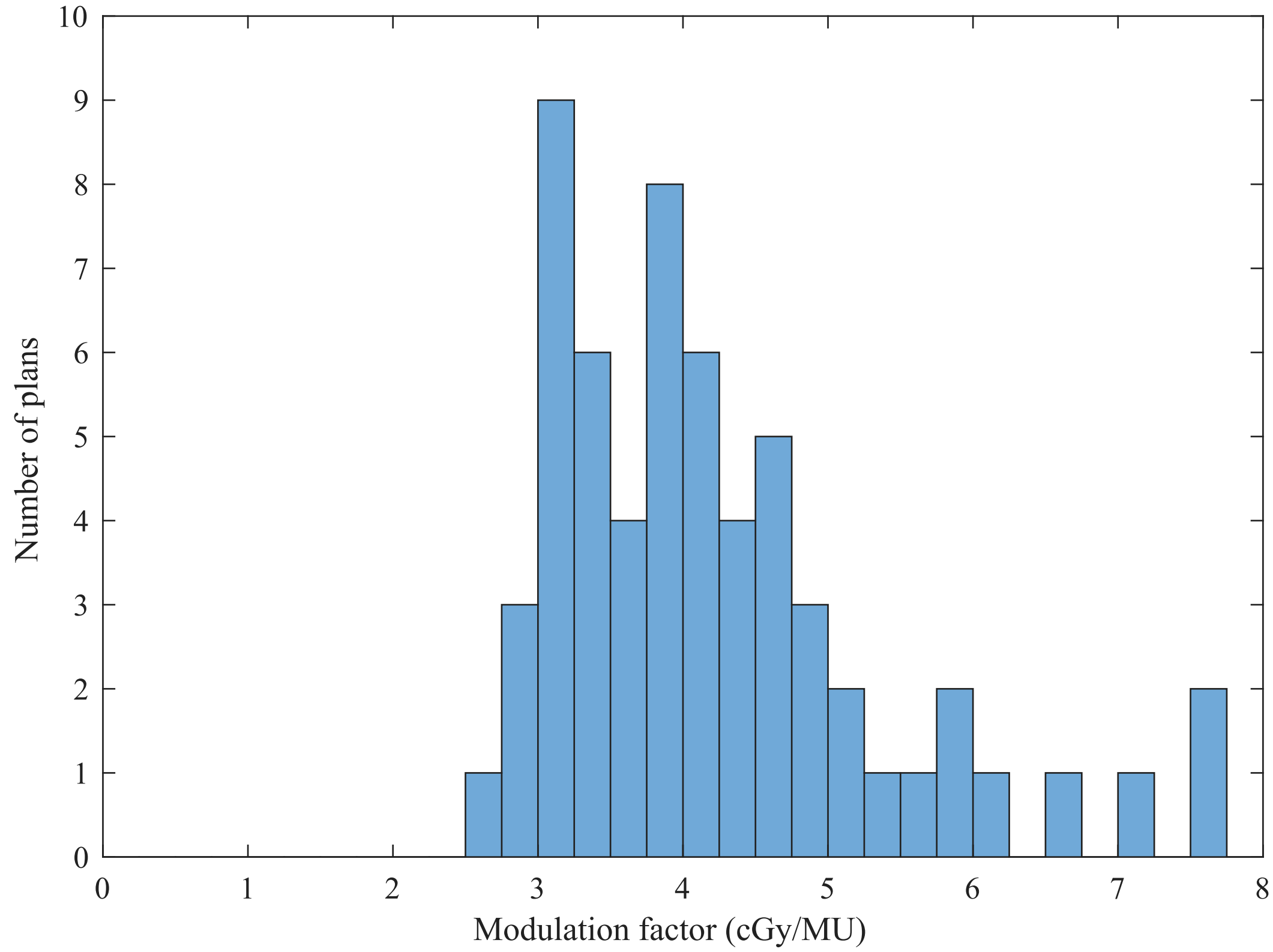

Figure S2a. Supplement to Figure 3

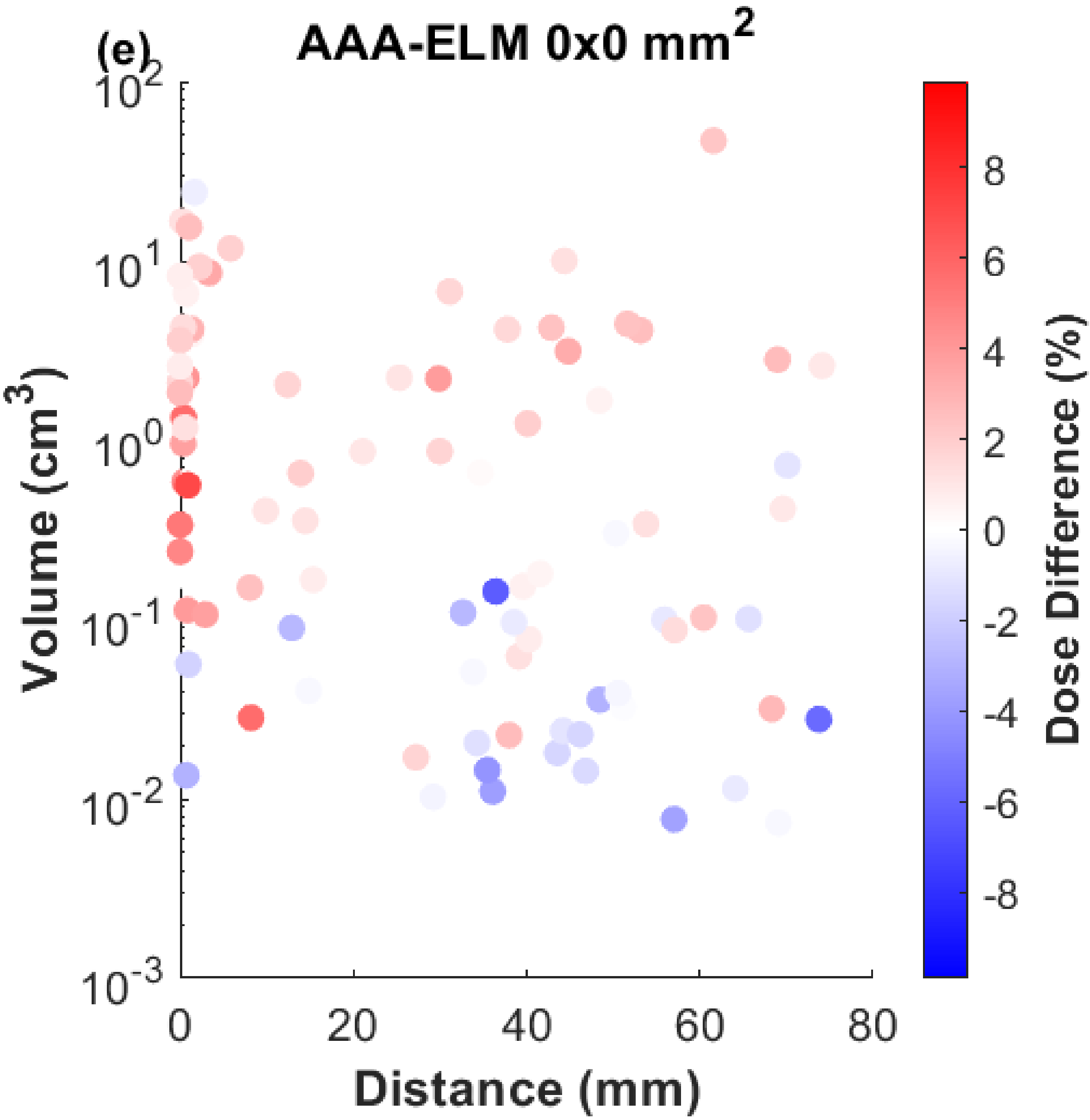

Figure S2b. Supplement to Figure 3

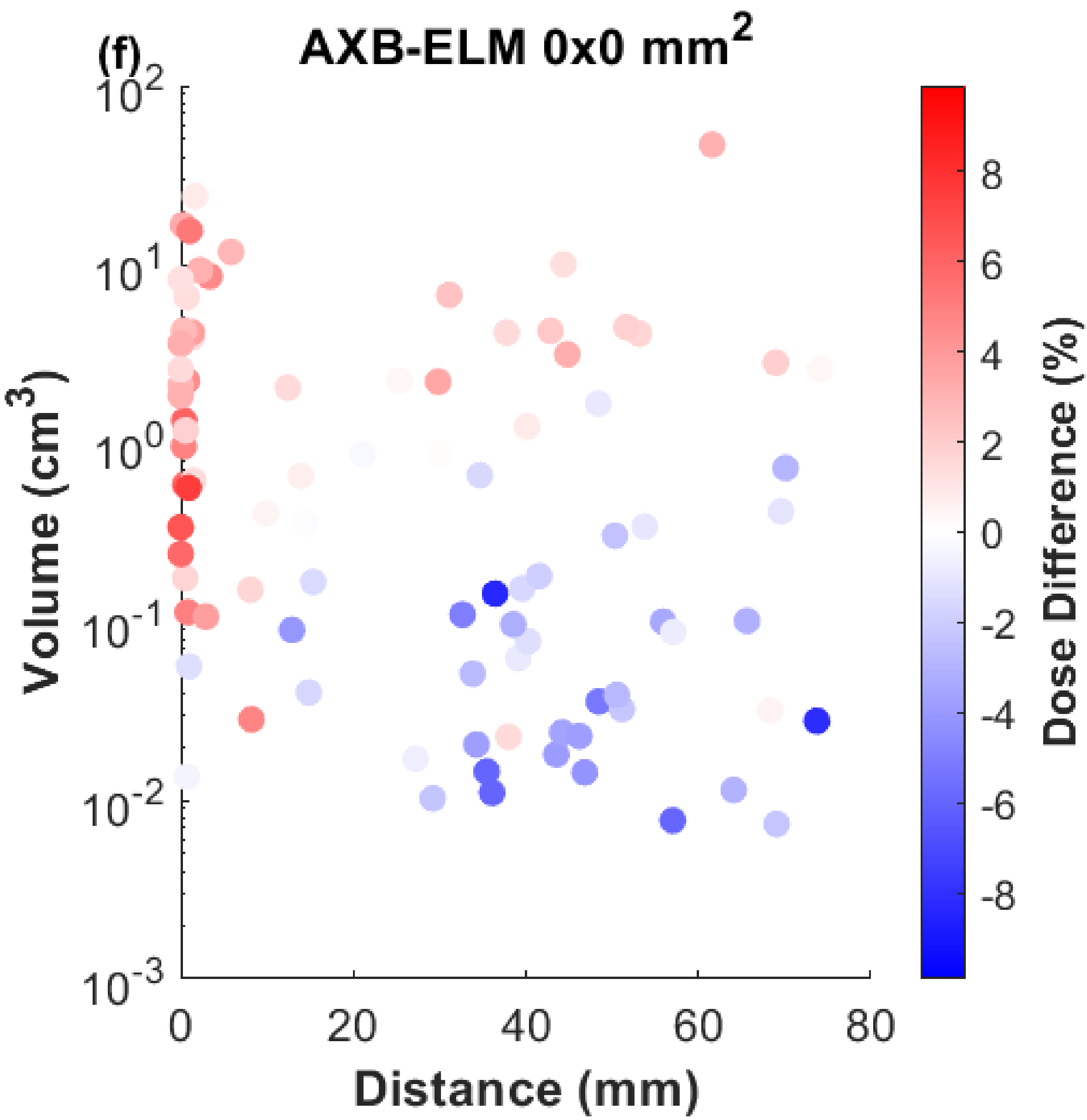

Figure S2c. Supplement to Figure 3

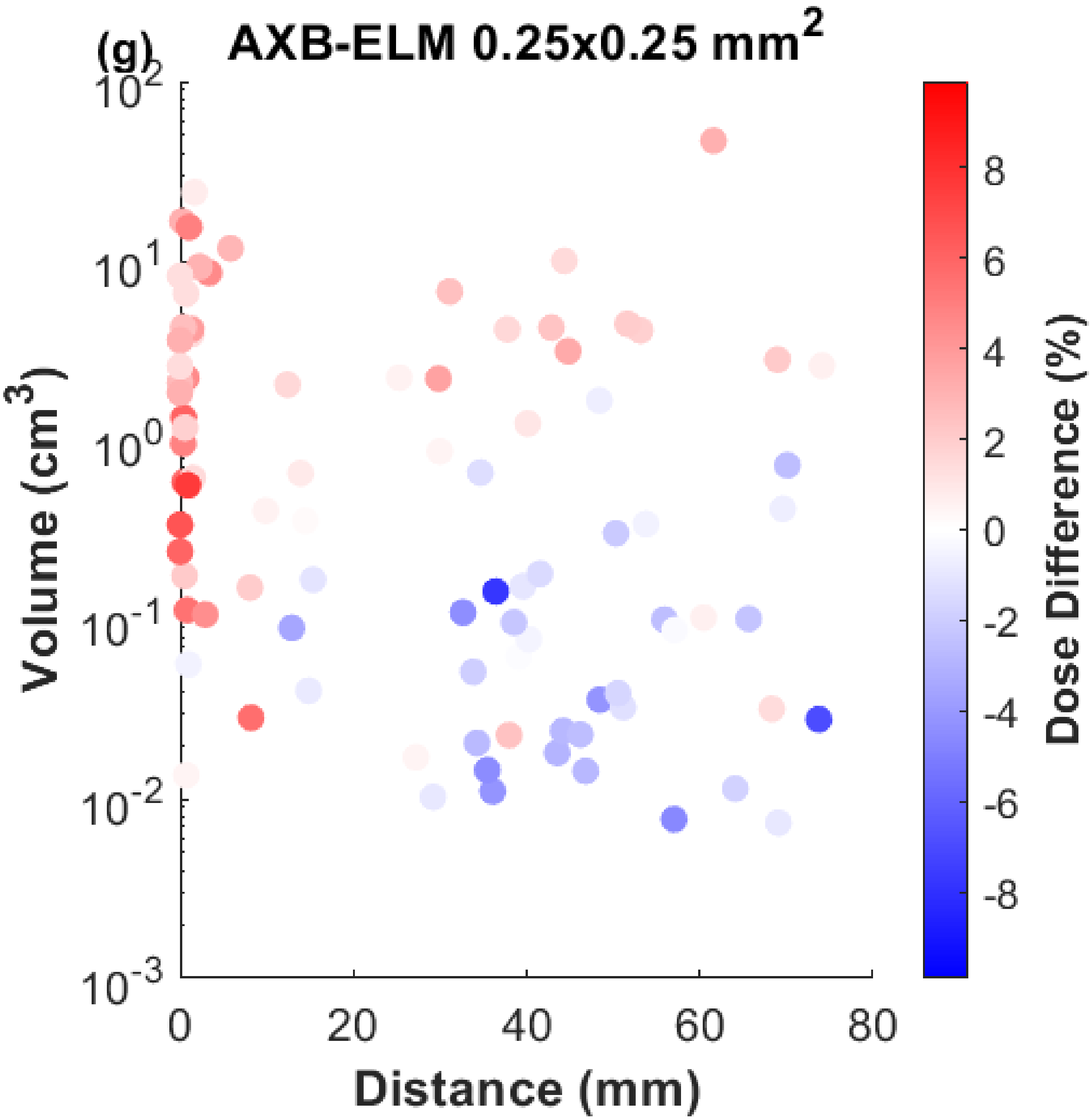

Figure S3. Measurement 1. Plan 1, 2 targets, equivalent diameter 8.8 mm. SRS MapCHECK fraction passing gamma 2%/1 mm = 100.0%

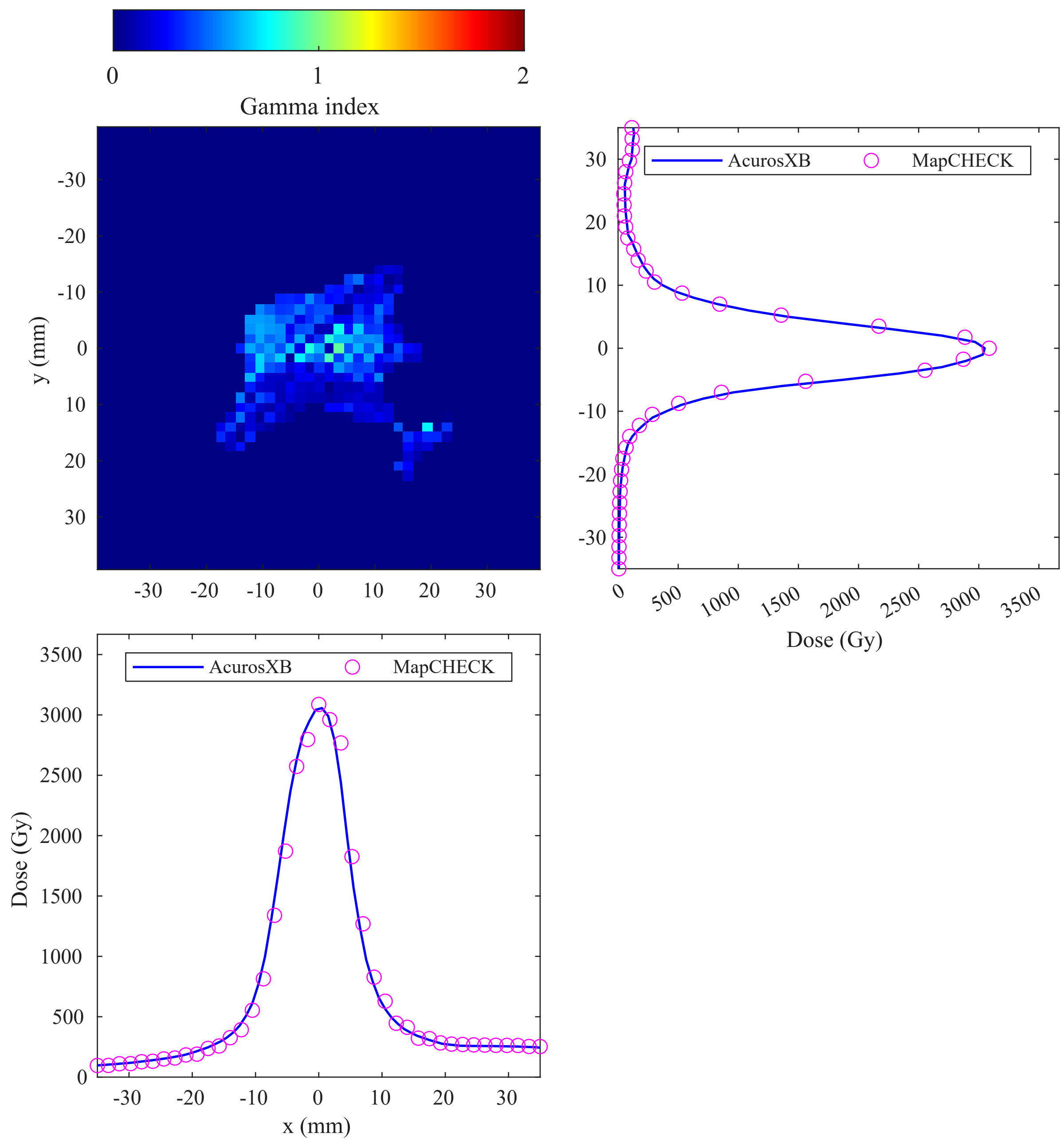

Figure S4. Measurement 2. Plan 1, 2 targets, equivalent diameter 4.2 mm. SRS MapCHECK fraction passing gamma 2%/1 mm = 94.8%

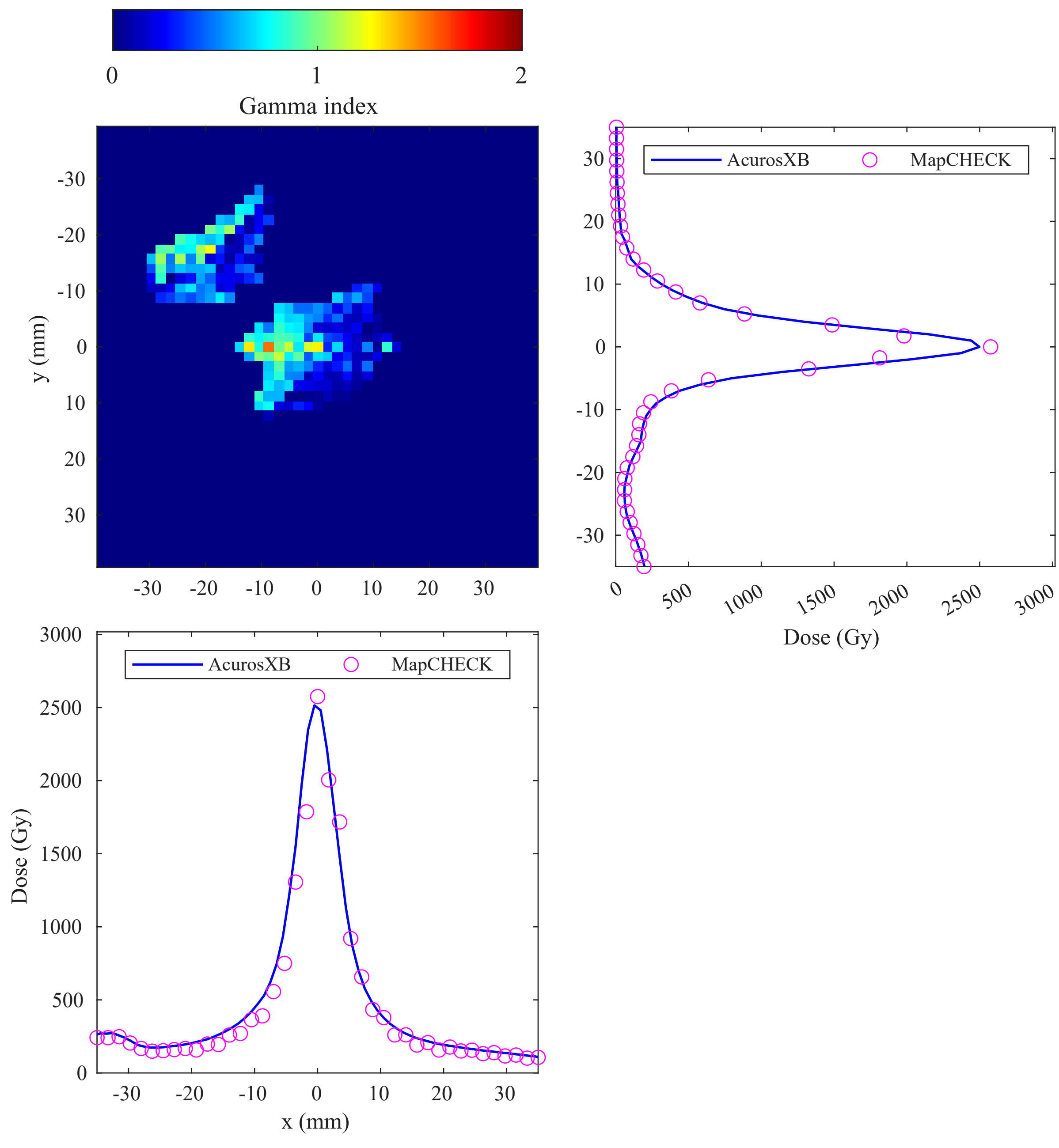

Figure S5. Measurement 3. Plan 2, 4 targets, equivalent diameter 19.9 mm. SRS MapCHECK fraction passing gamma 2%/1 mm = 99.0%

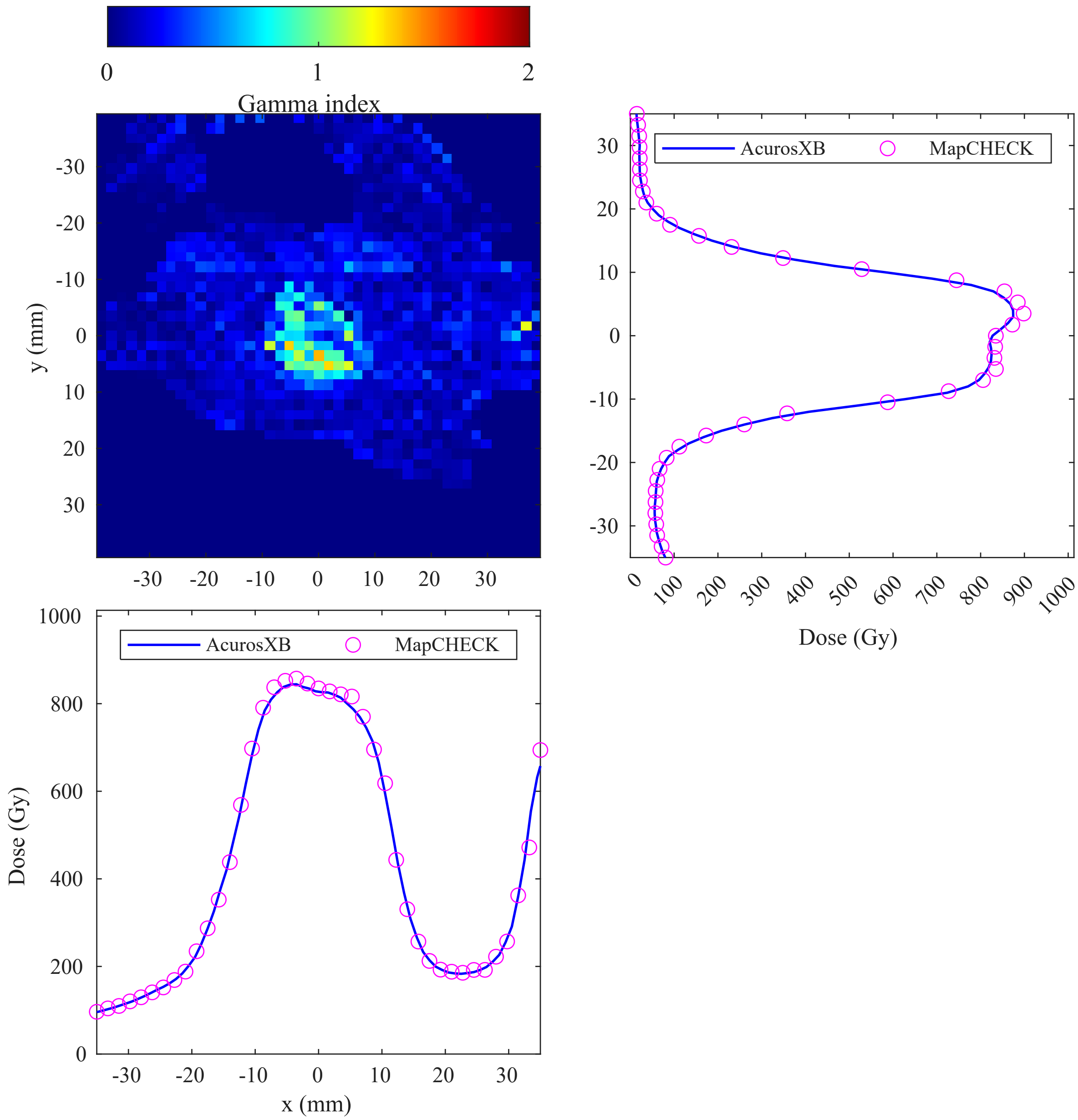

Figure S6. Measurement 4. Plan 2, 4 targets, equivalent diameter 4.9 mm. SRS MapCHECK fraction passing gamma 2%/1 mm = 99.5%

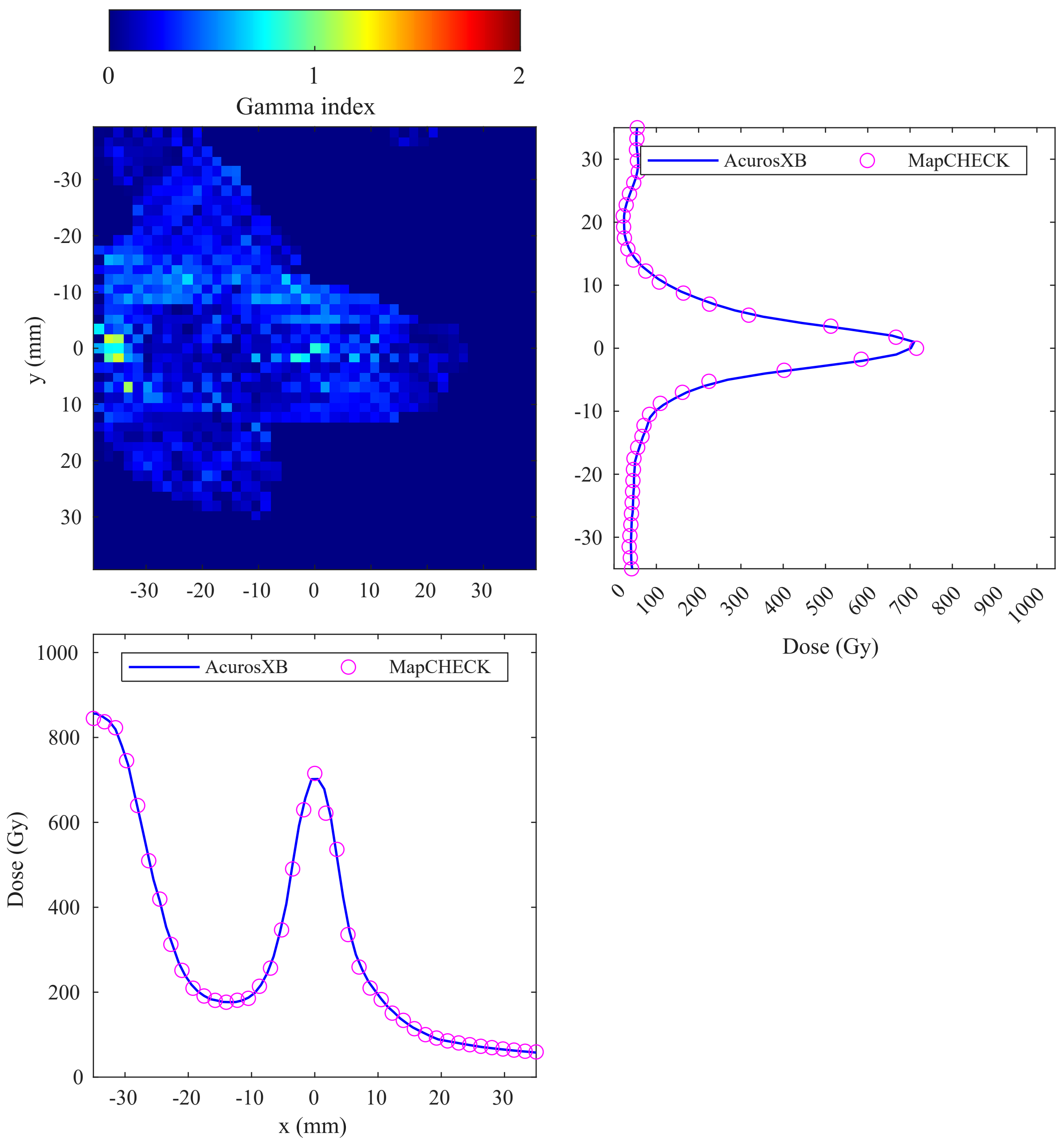

Figure S7. Measurement 5. Plan 3, single target, equivalent diameter 6.9 mm. SRS MapCHECK fraction passing gamma 2%/1 mm = 96.8%

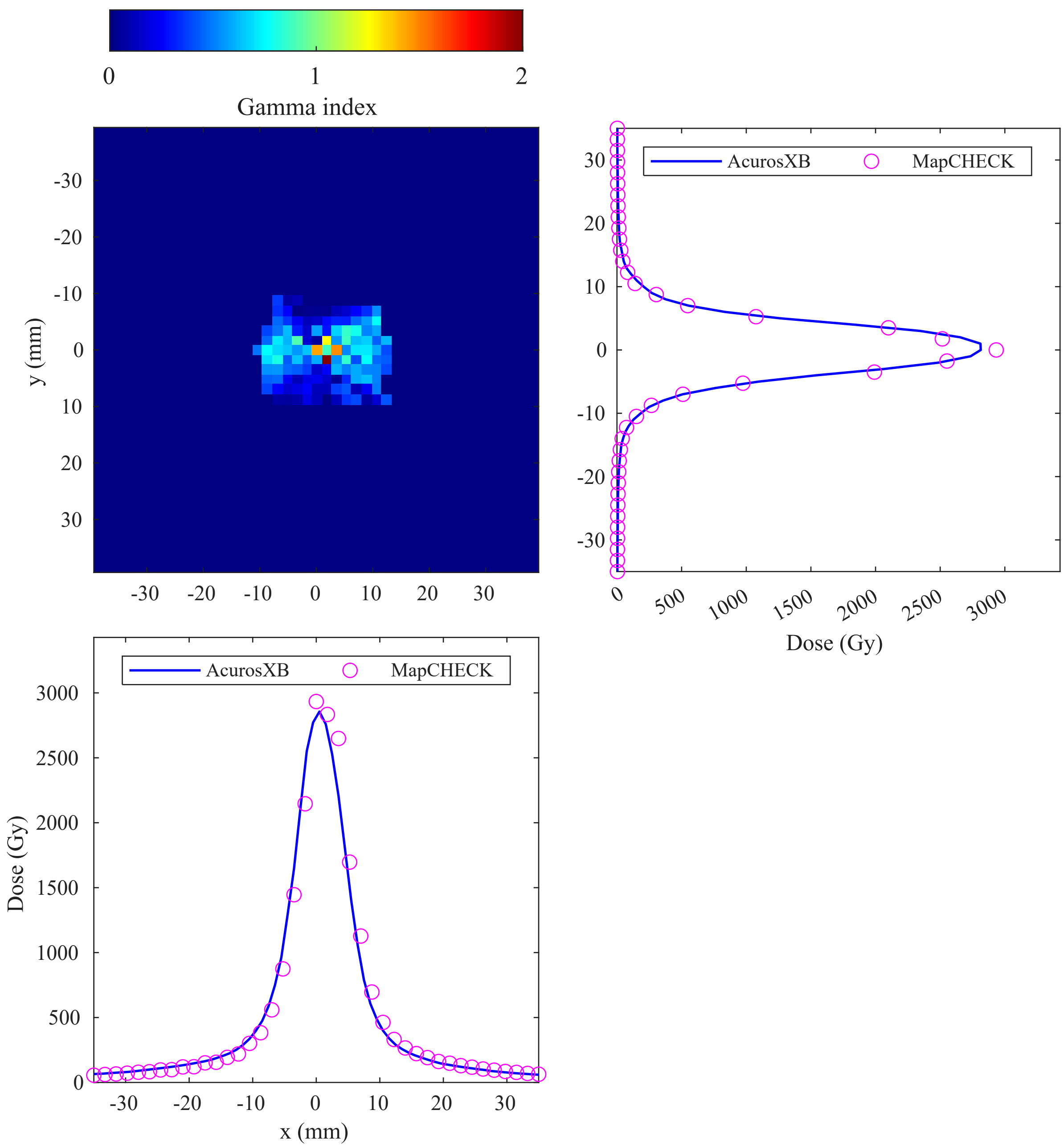

Figure S8. Measurement 6. Plan 4, single target, equivalent diameter 4.8 mm. SRS MapCHECK fraction passing gamma 2%/1 mm = 98.9%

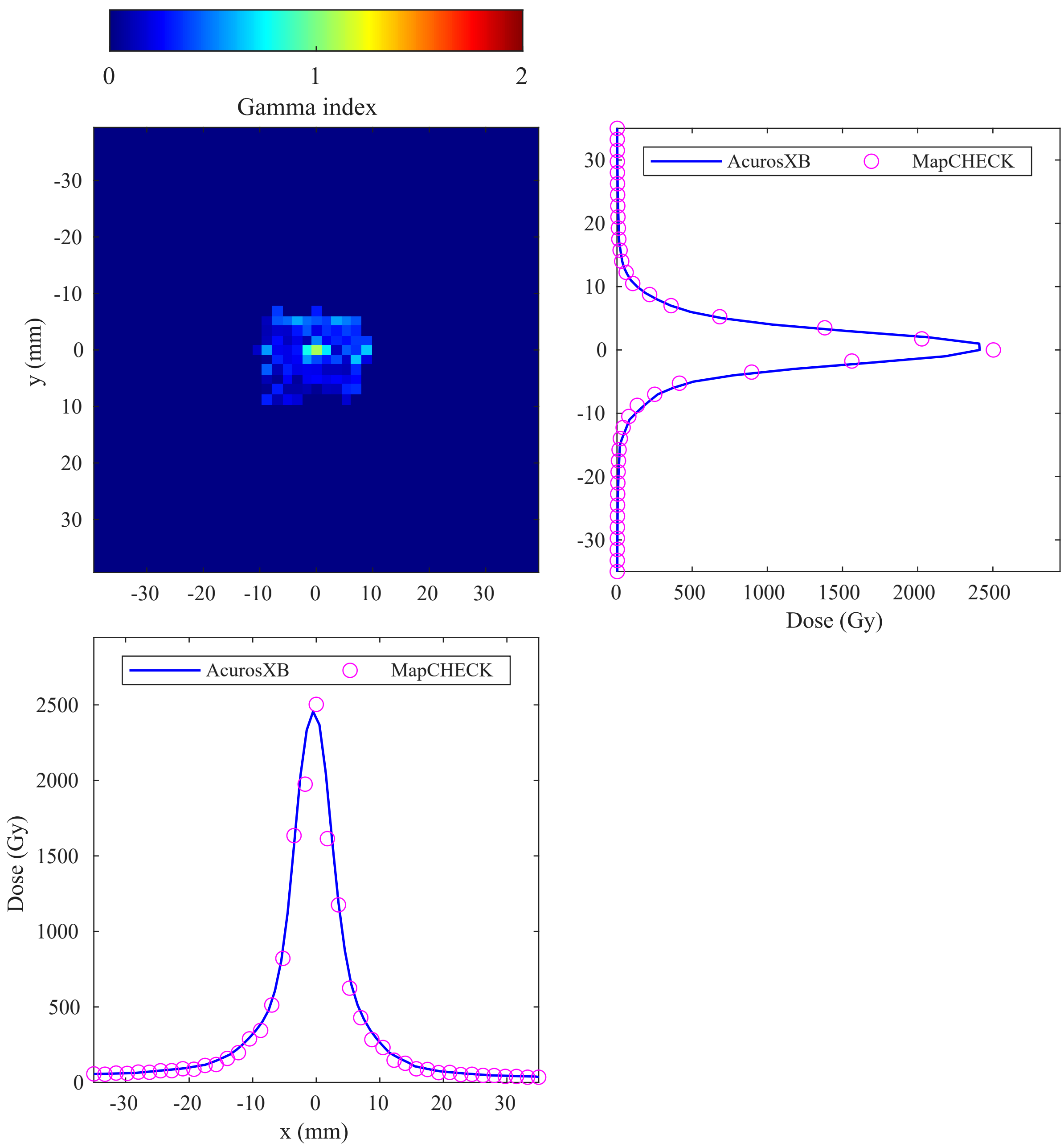

Figure S9. Measurement 7. Plan 5, 5 targets, equivalent diameter 11.8 mm. SRS MapCHECK fraction passing gamma 2%/1 mm = 98.2%

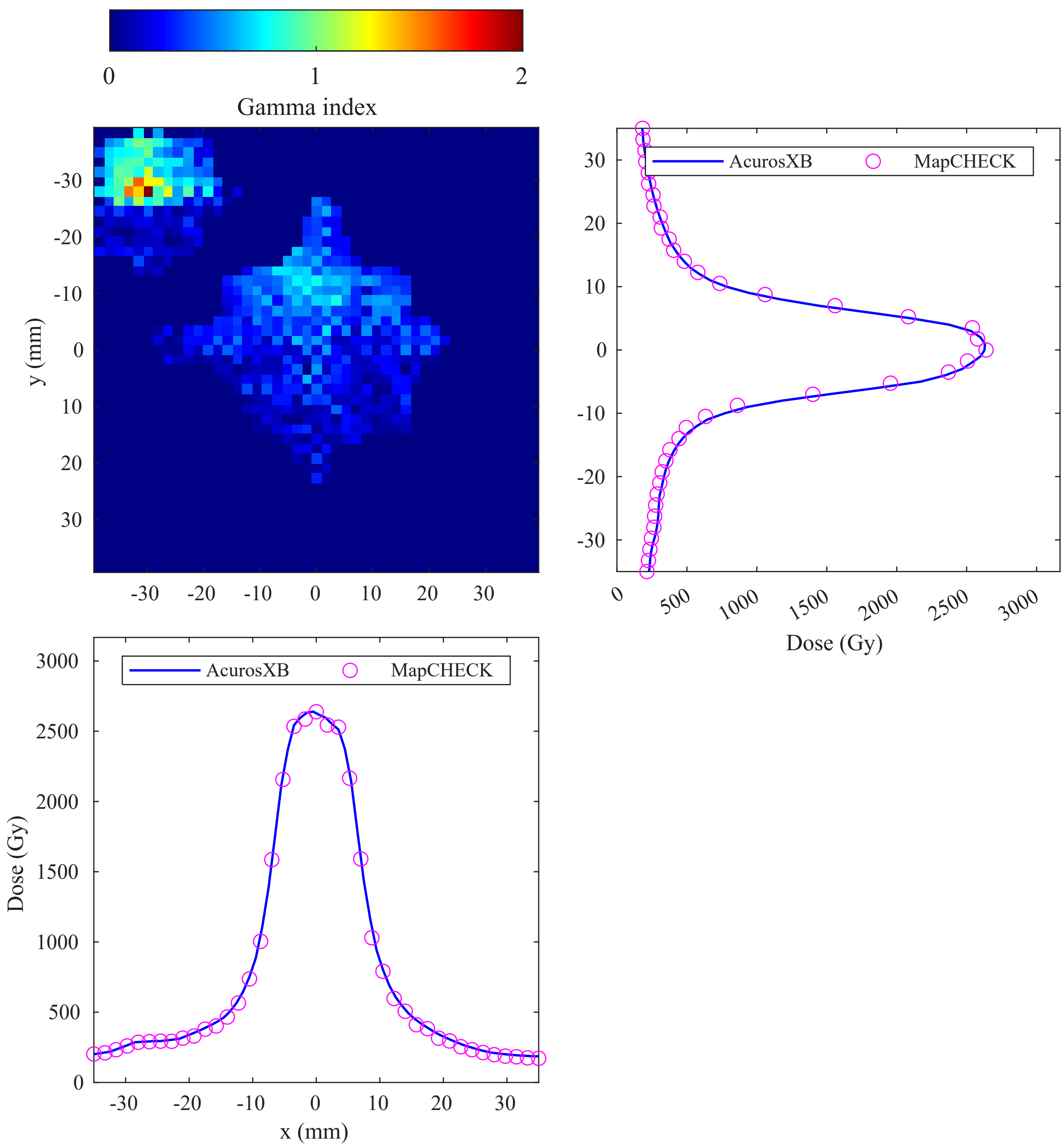

Figure S10. Measurement 8. Plan 5, 5 targets, equivalent diameter 3.0 mm. SRS MapCHECK fraction passing gamma 2%/1 mm = 99.2%

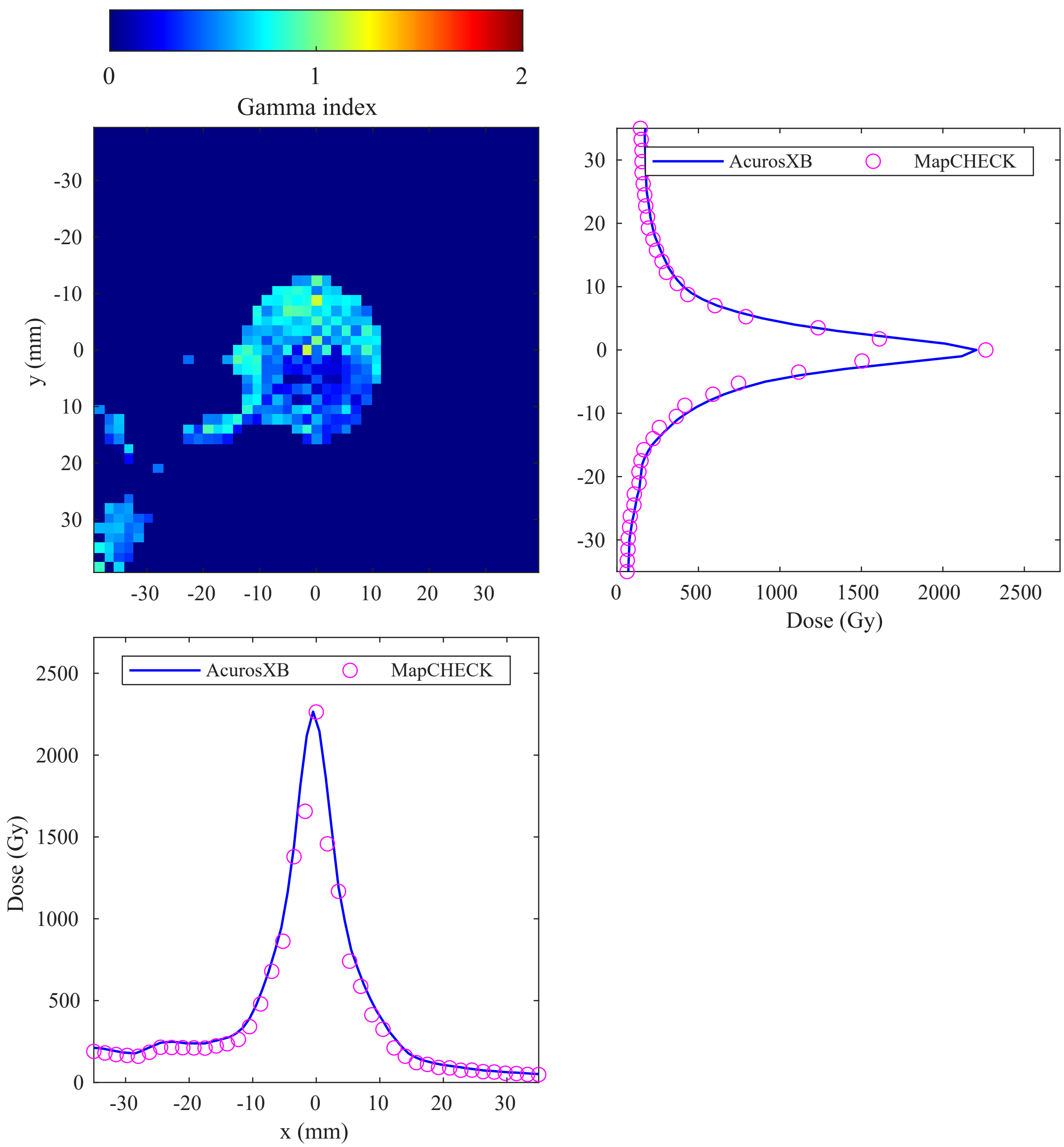

Figure S11. Measurement 9. Plan 6, single target, equivalent diameter 25.4 mm. SRS MapCHECK fraction passing gamma 2%/1 mm = 89.2%

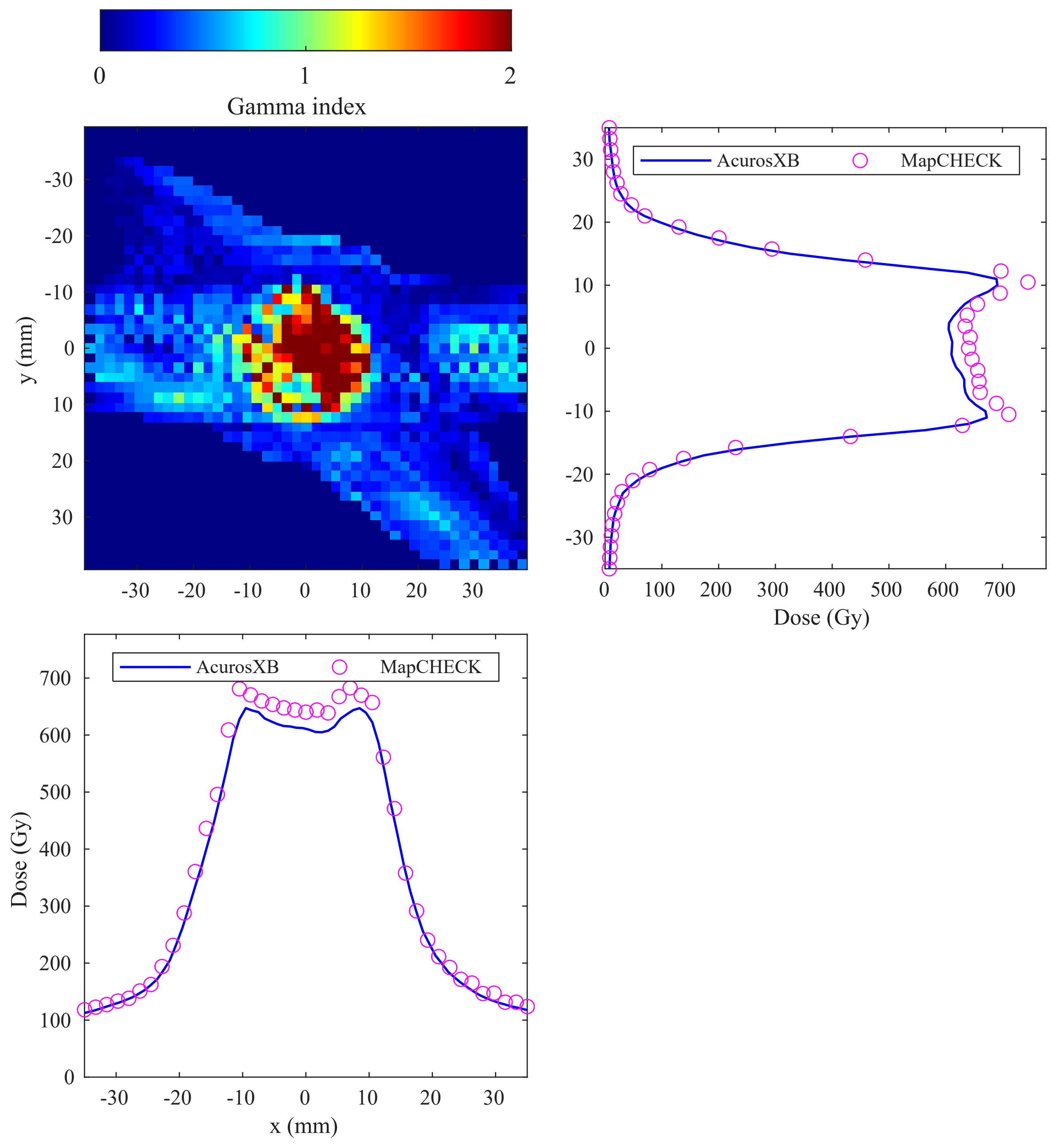

Figure S12. Measurement 10. Plan 7, 9 targets, equivalent diameter 2.8 mm. SRS MapCHECK fraction passing gamma 2%/1 mm = 83.2%

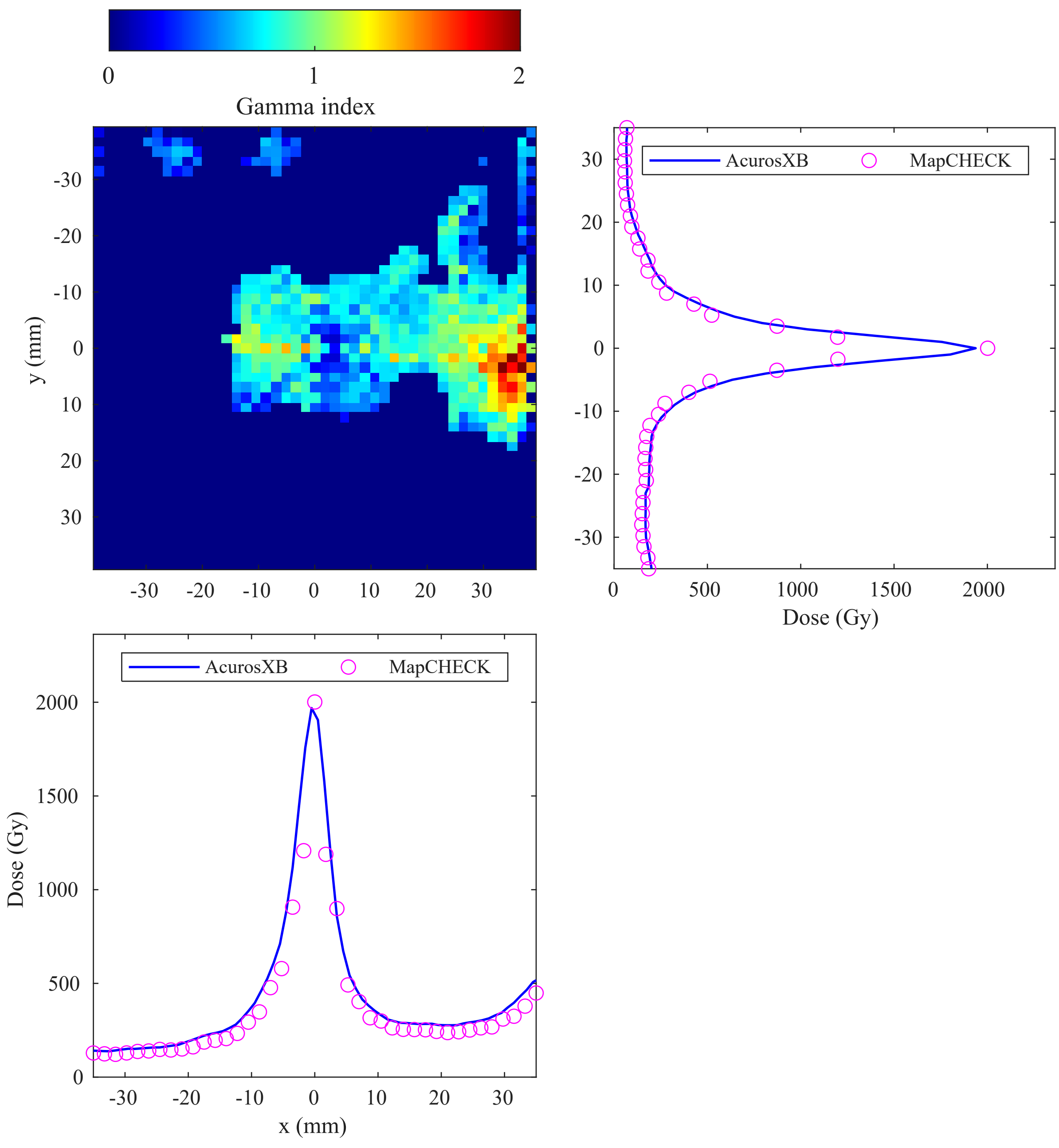

Figure S13. Measurement 11. Plan 7, 9 targets, equivalent diameter 5.8 mm. SRS MapCHECK fraction passing gamma 2%/1 mm = 93.9%

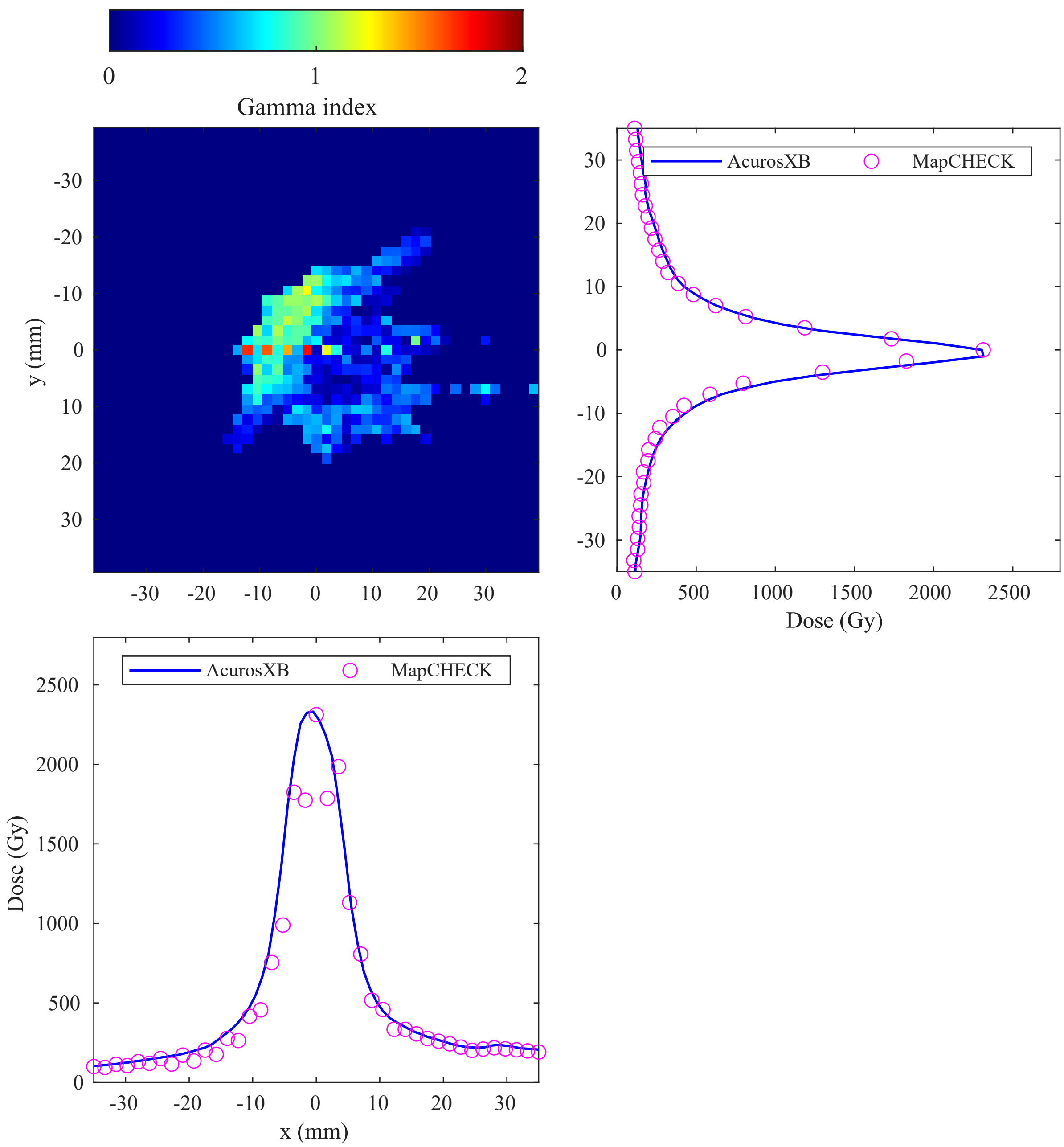

Figure S14. Measurement 12. Plan 8, single target, equivalent diameter 19.5 mm. SRS MapCHECK fraction passing gamma 2%/1 mm = 97.6%

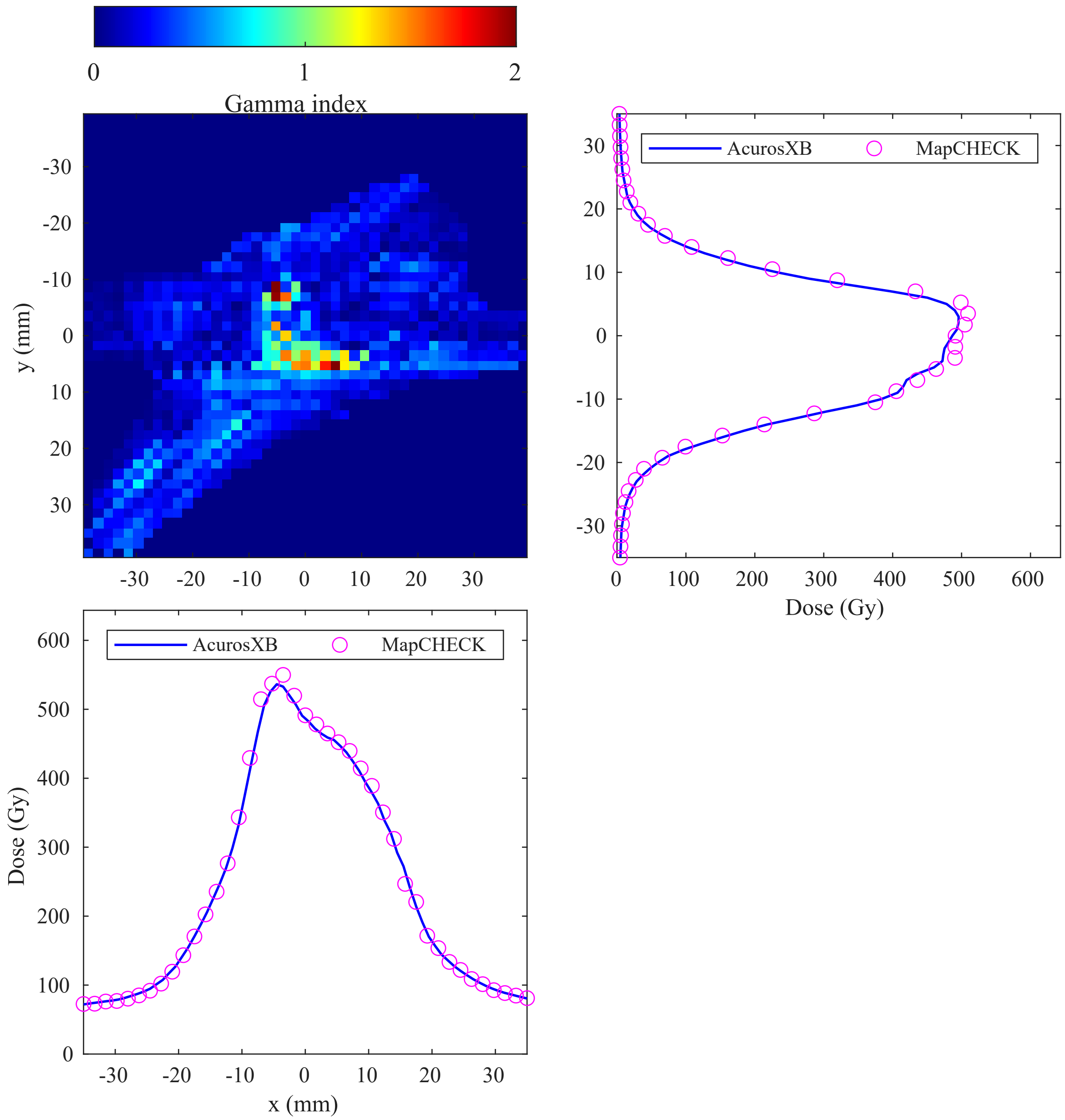

Figure S15. Measurement 13. Plan 9, 9 targets, equivalent diameter 20.1 mm. SRS MapCHECK fraction passing gamma 2%/1 mm = 96.9%

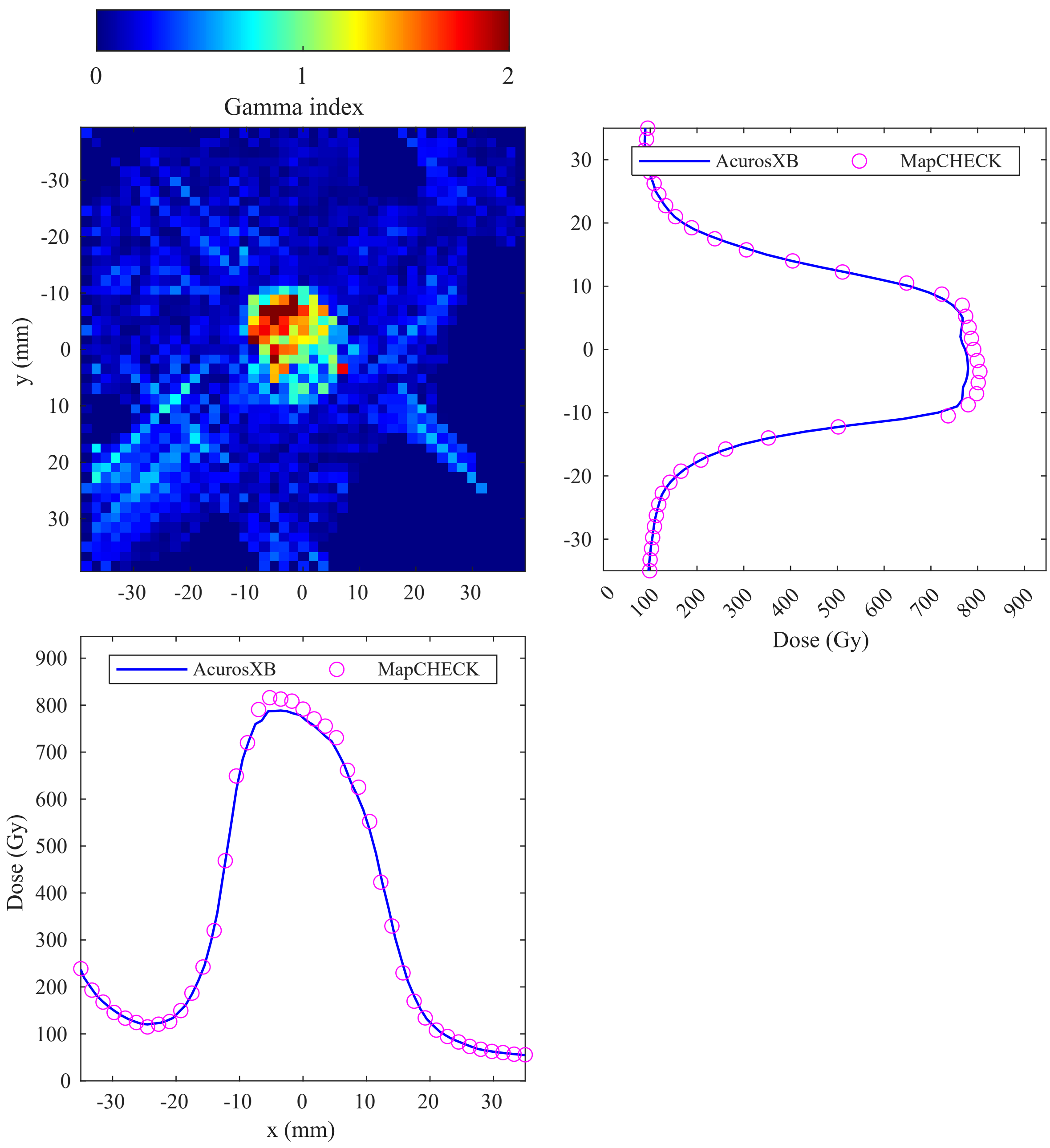

Figure S16. Measurement 14. Plan 9, 9 targets, equivalent diameter 5.3 mm. SRS MapCHECK fraction passing gamma 2%/1 mm = 99.7%

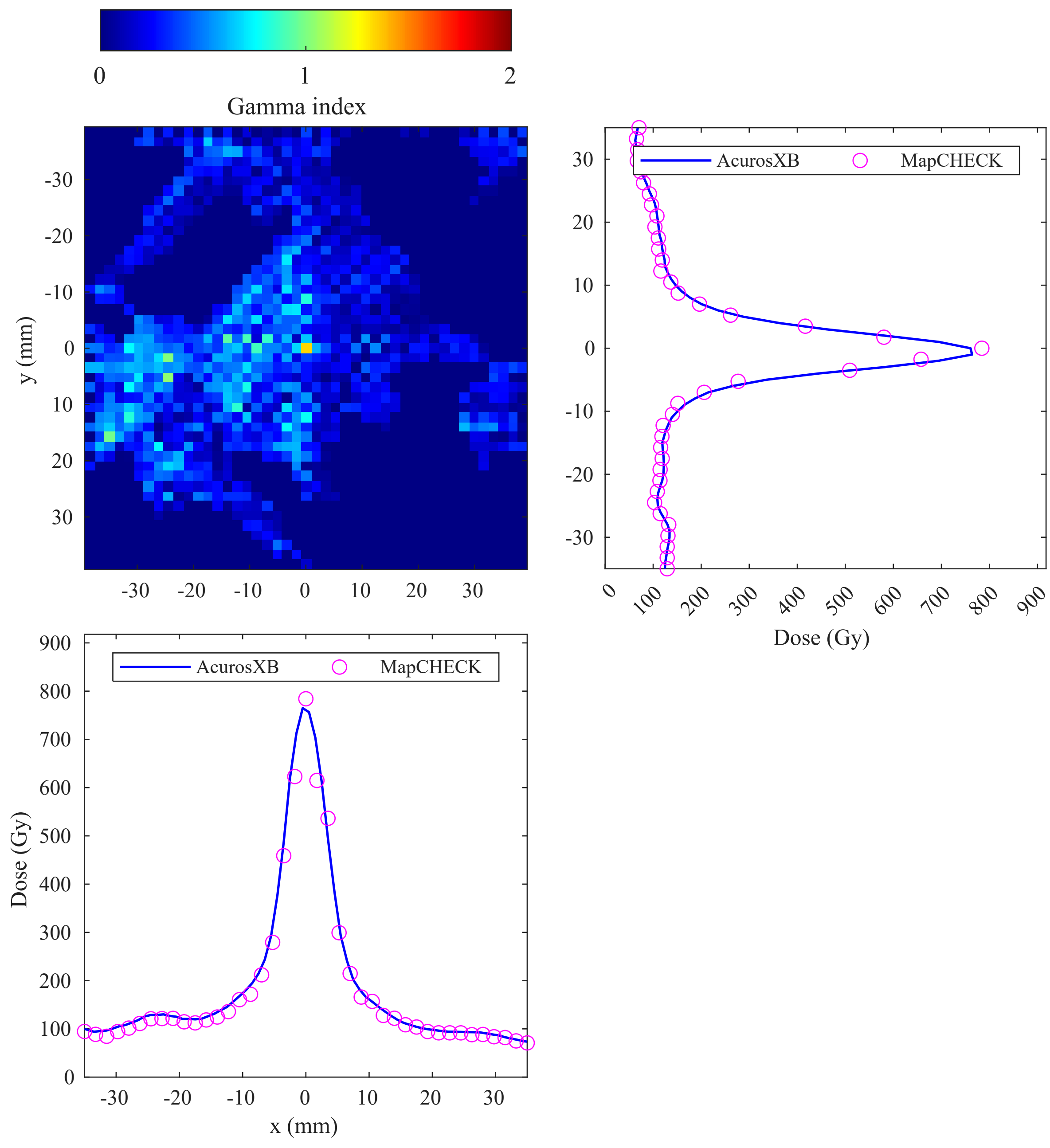

Figure S17. Measurement 15. Plan 10, single target, equivalent diameter 13.6 mm. SRS MapCHECK fraction passing gamma 2%/1 mm = 90.9%

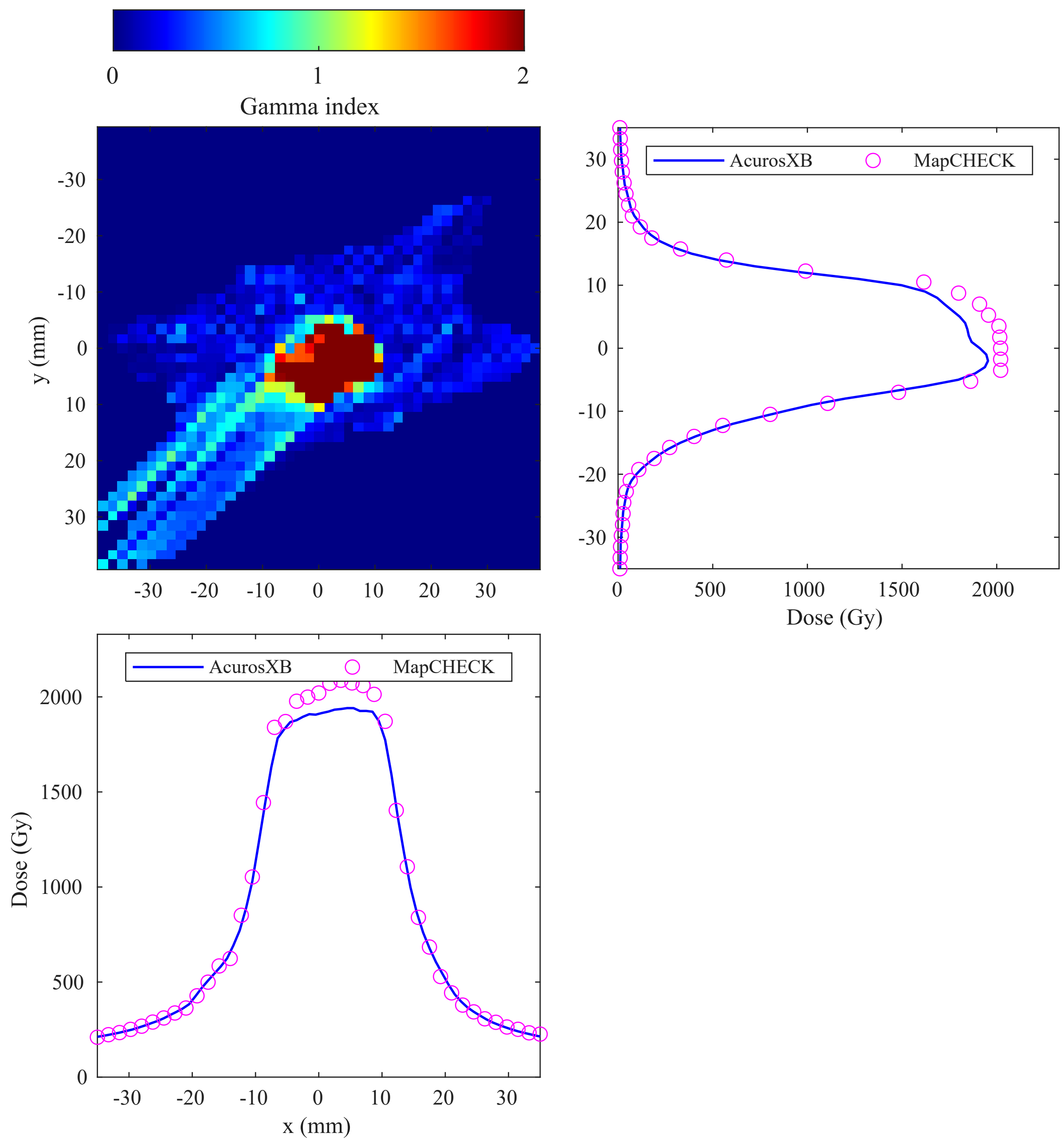

Figure S18. Measurement 16. Plan 11, single target, equivalent diameter 26.1 mm. SRS MapCHECK fraction passing gamma 2%/1 mm = 90.5%

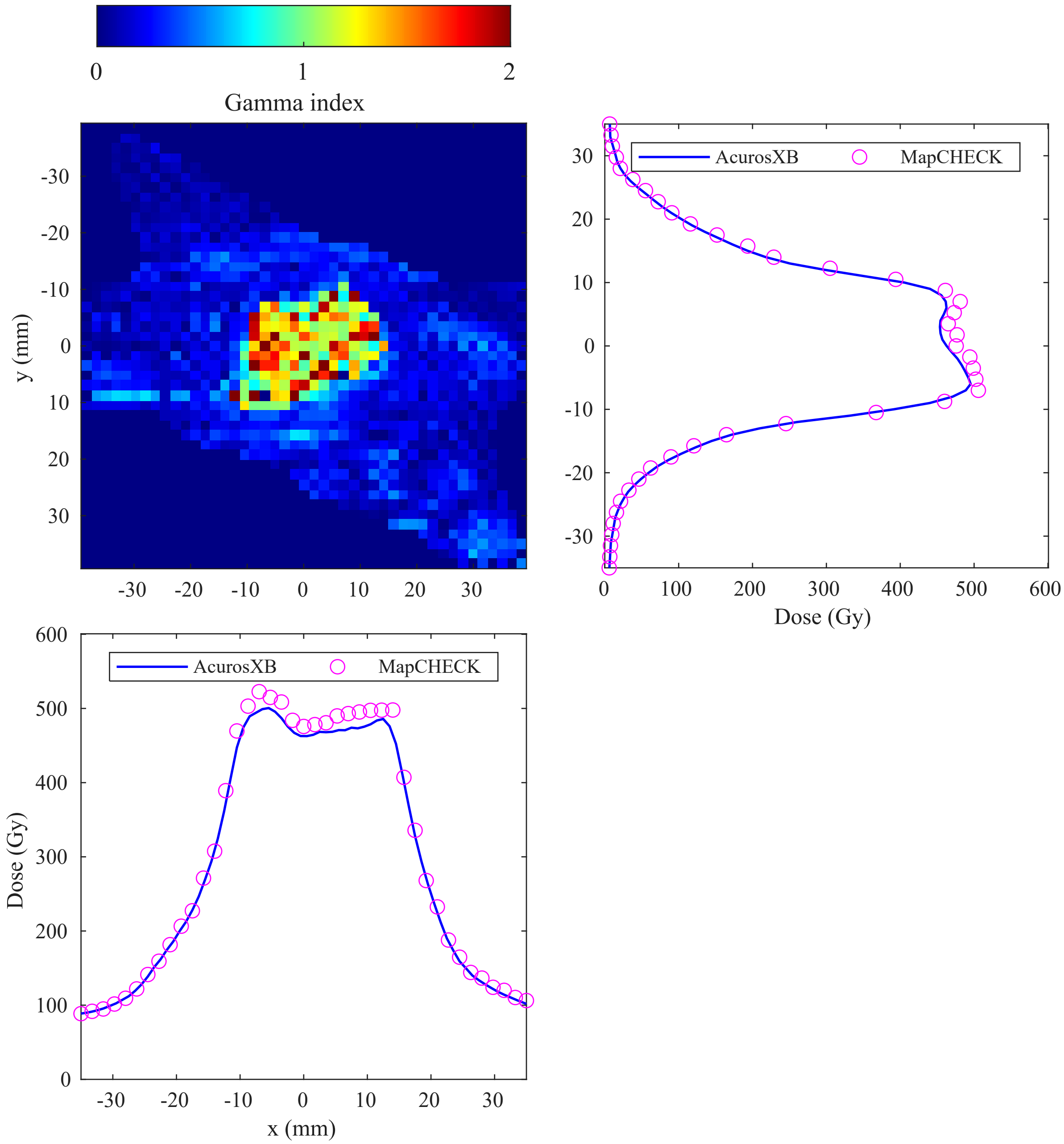

Figure S19. Measurement 17. Plan 12, 9 targets, equivalent diameter 3.0 mm. SRS MapCHECK fraction passing gamma 2%/1 mm = 70.2%

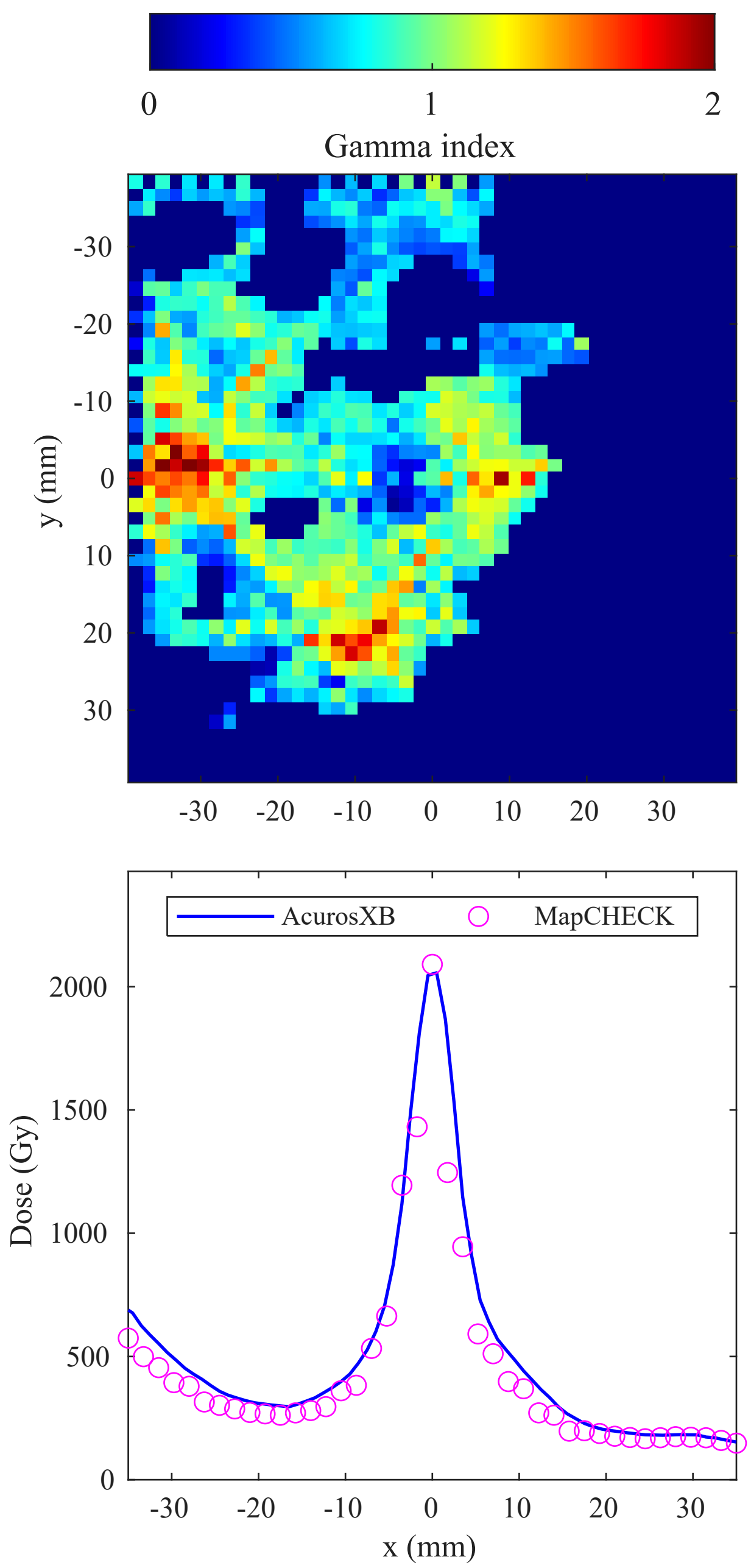

Figure S20. Measurement 18. Plan 12, 9 targets, equivalent diameter 6.5 mm. SRS MapCHECK fraction passing gamma 2%/1 mm = 80.2%

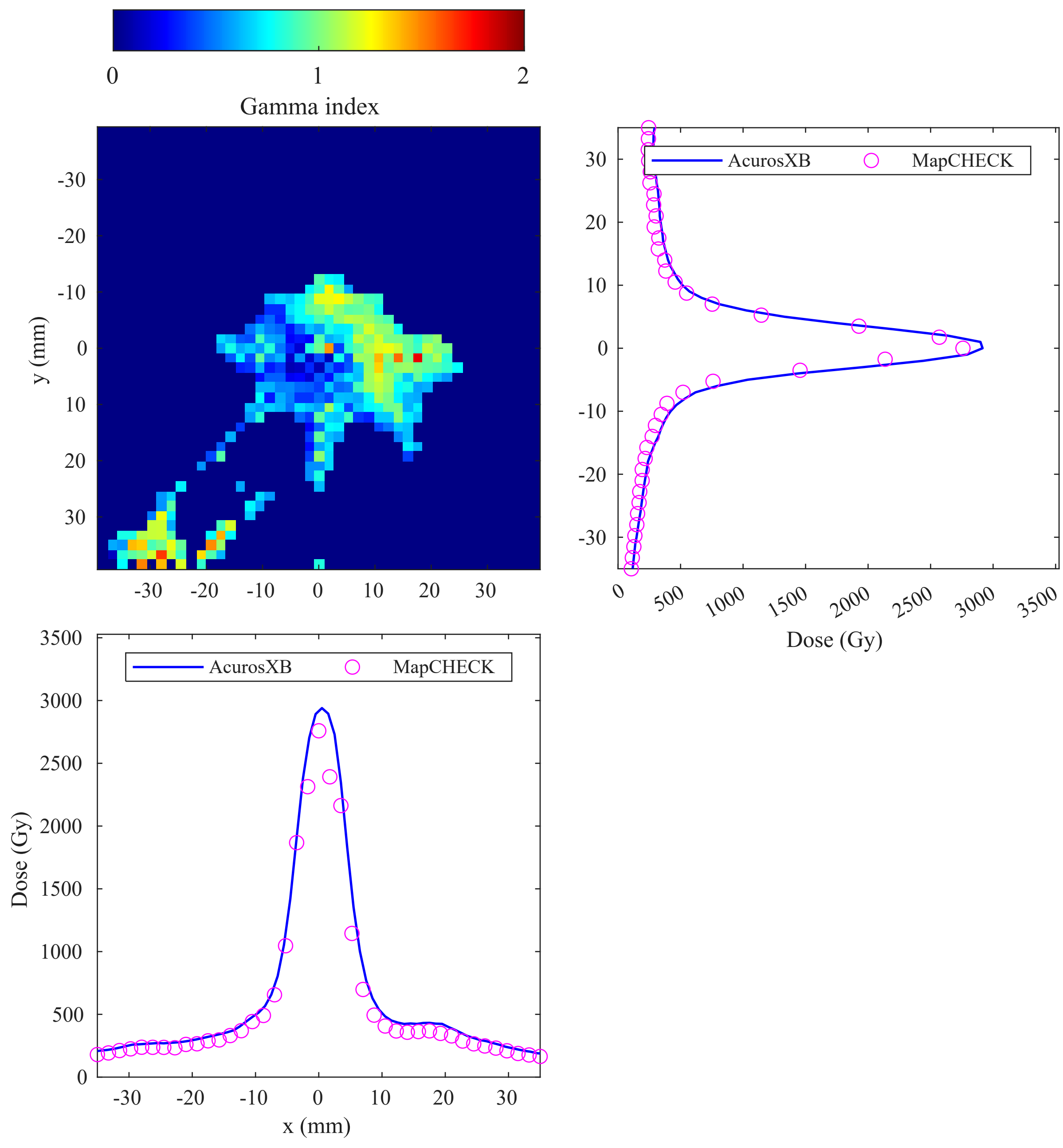

Figure S21. Measurement 19. Plan 13, single target, equivalent diameter 35.8 mm. SRS MapCHECK fraction passing gamma 2%/1 mm = 96.4%

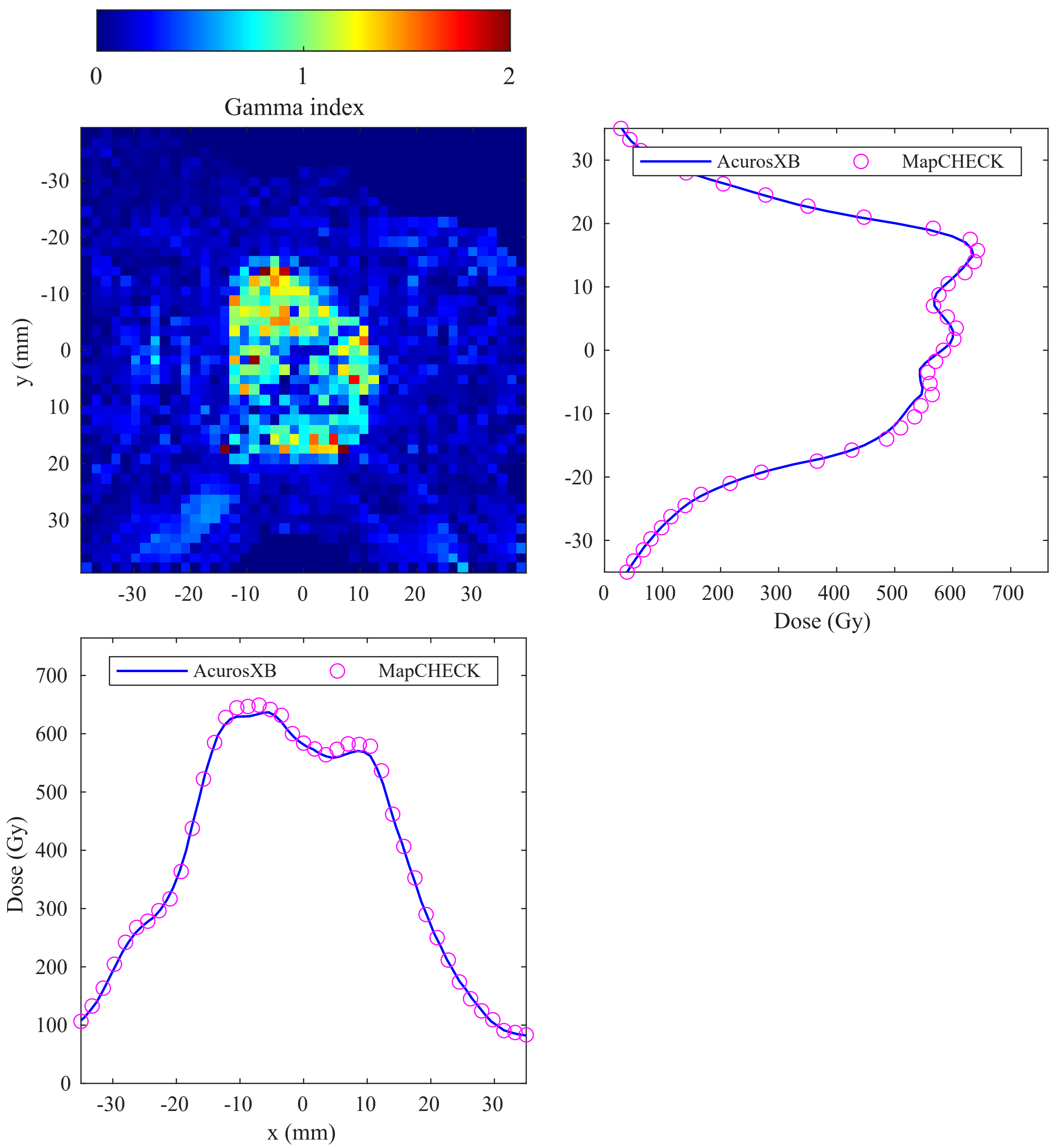

Figure S22. Measurement 20. Plan 14, 7 targets, equivalent diameter 17.5 mm. SRS MapCHECK fraction passing gamma 2%/1 mm = 96.7%

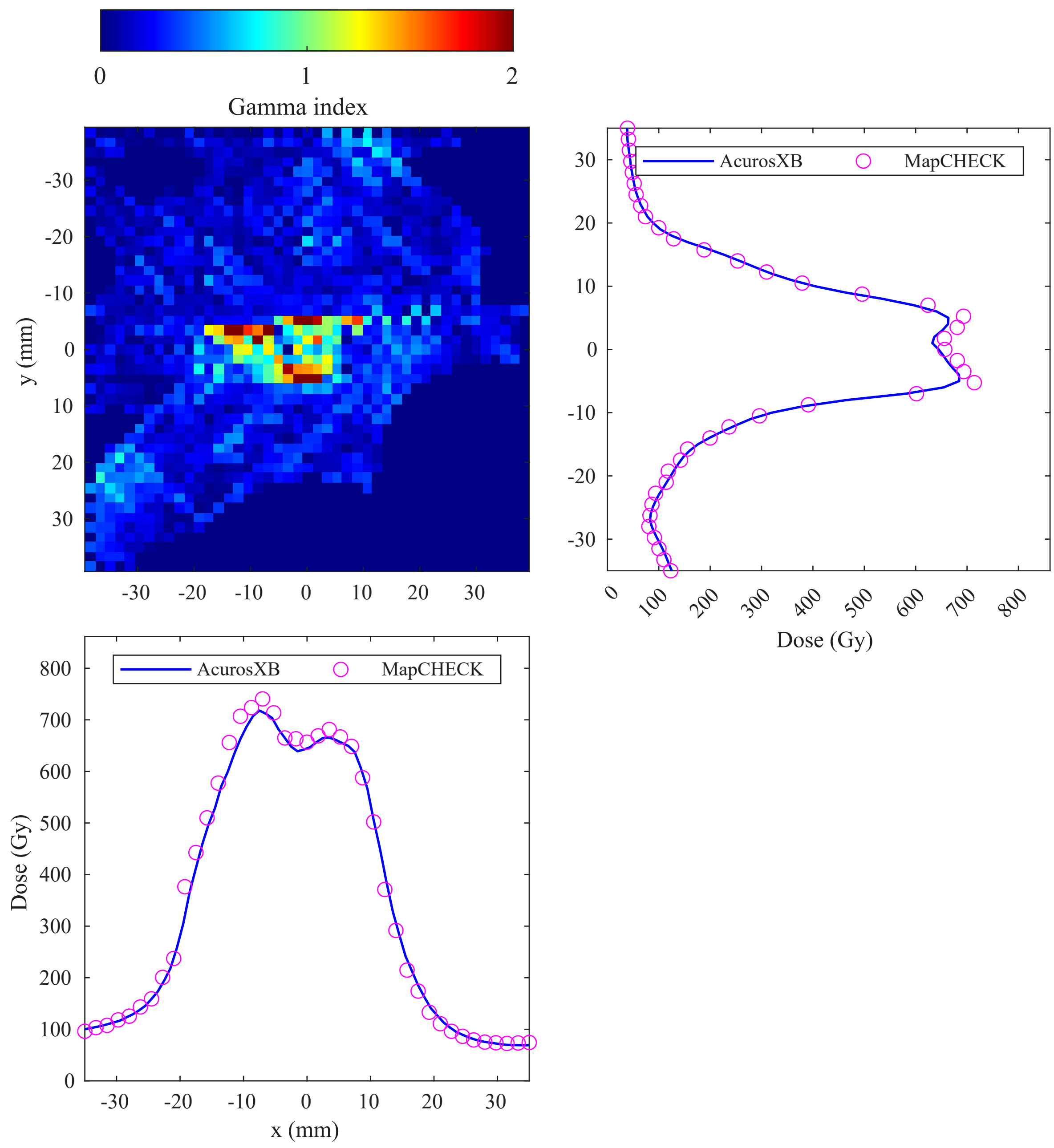

Figure S23. Measurement 21. Plan 14, 7 targets, equivalent diameter 4.1 mm. SRS MapCHECK fraction passing gamma 2%/1 mm = 99.2%

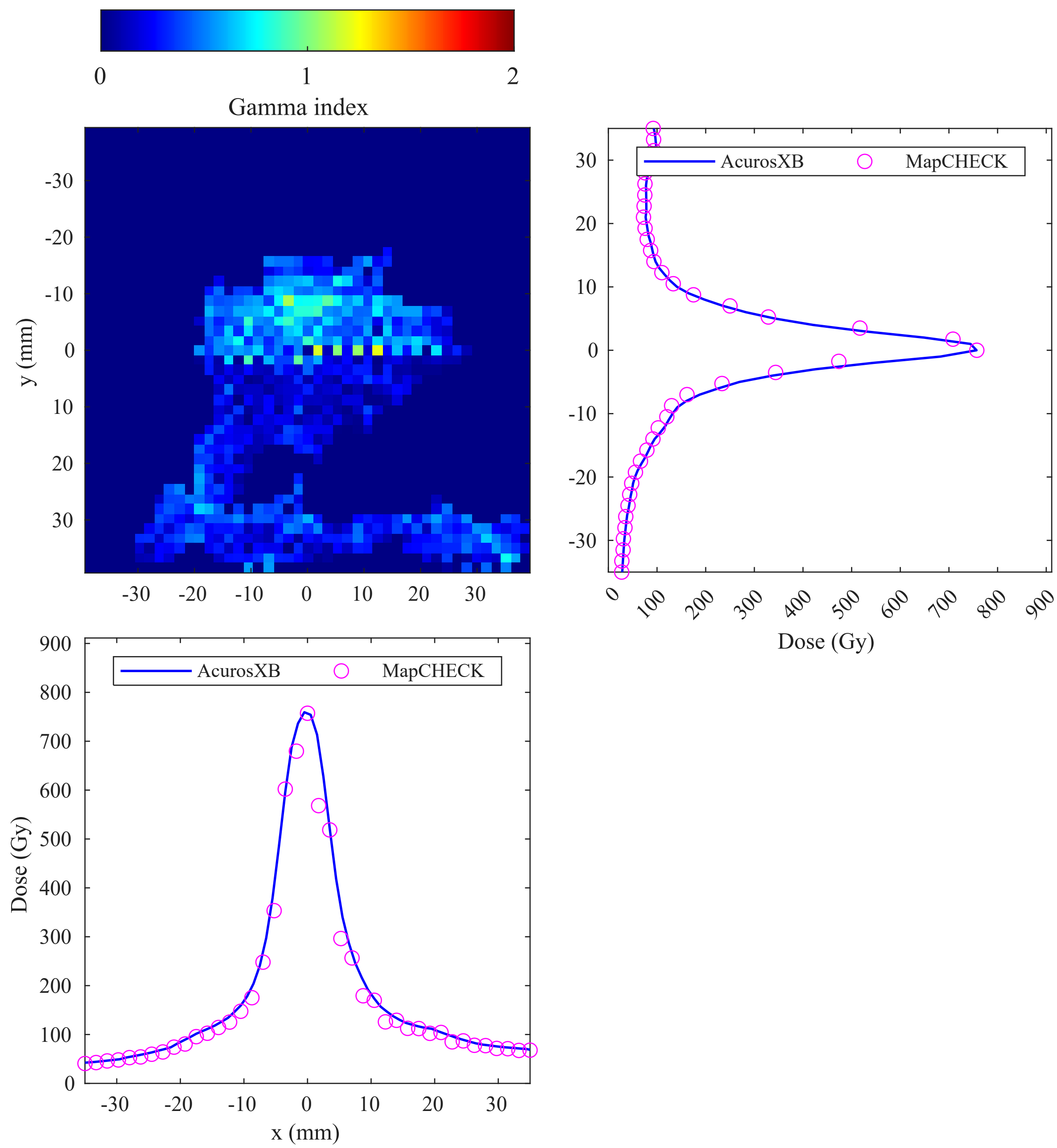

Figure S24. Measurement 22. Plan 15, 8 targets, equivalent diameter 16.1 mm. SRS MapCHECK fraction passing gamma 2%/1 mm = 96.6%

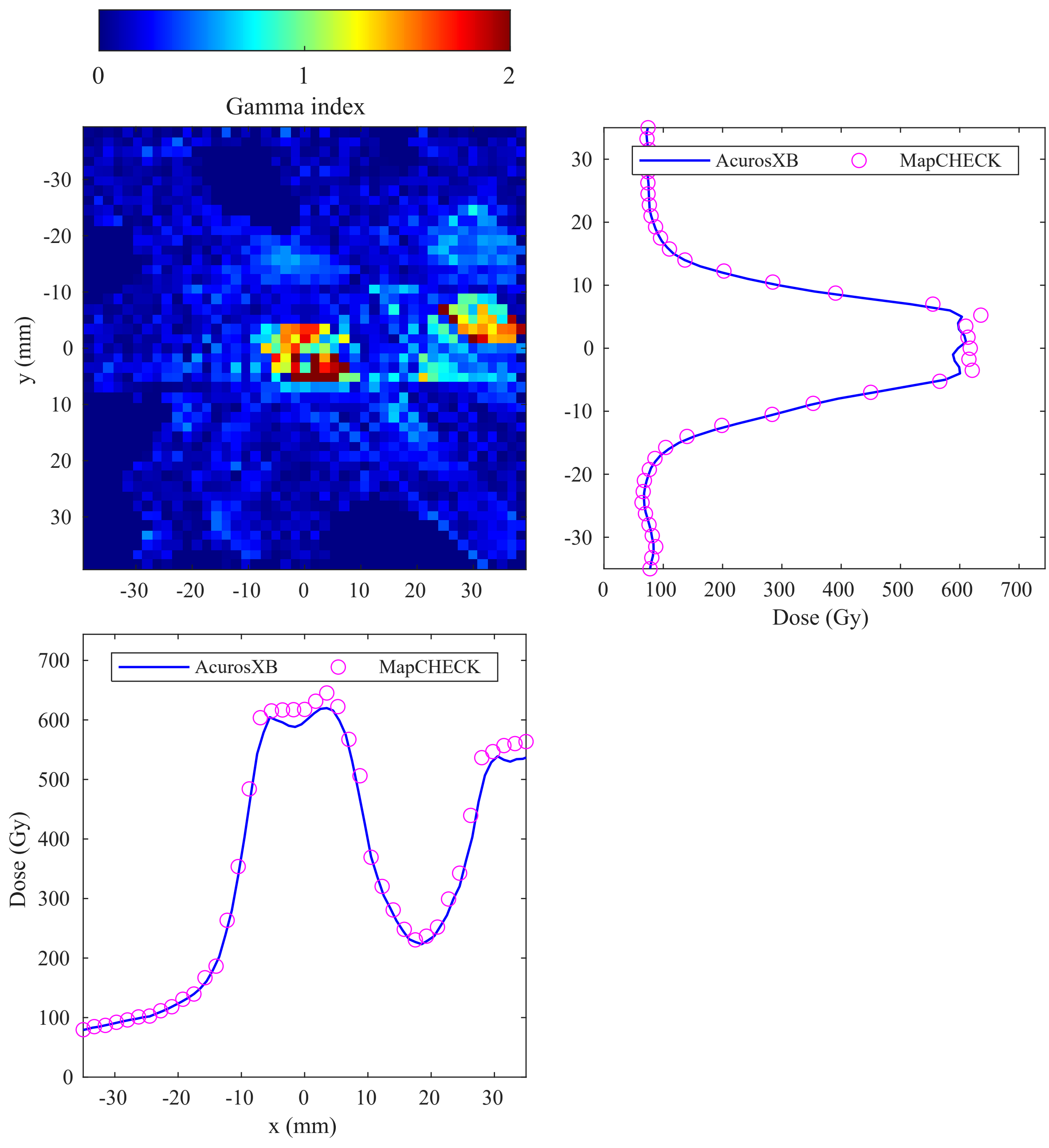

Figure S25. Measurement 23. Plan 15, 8 targets, equivalent diameter 9.2 mm. SRS MapCHECK fraction passing gamma 2%/1 mm = 100.0%

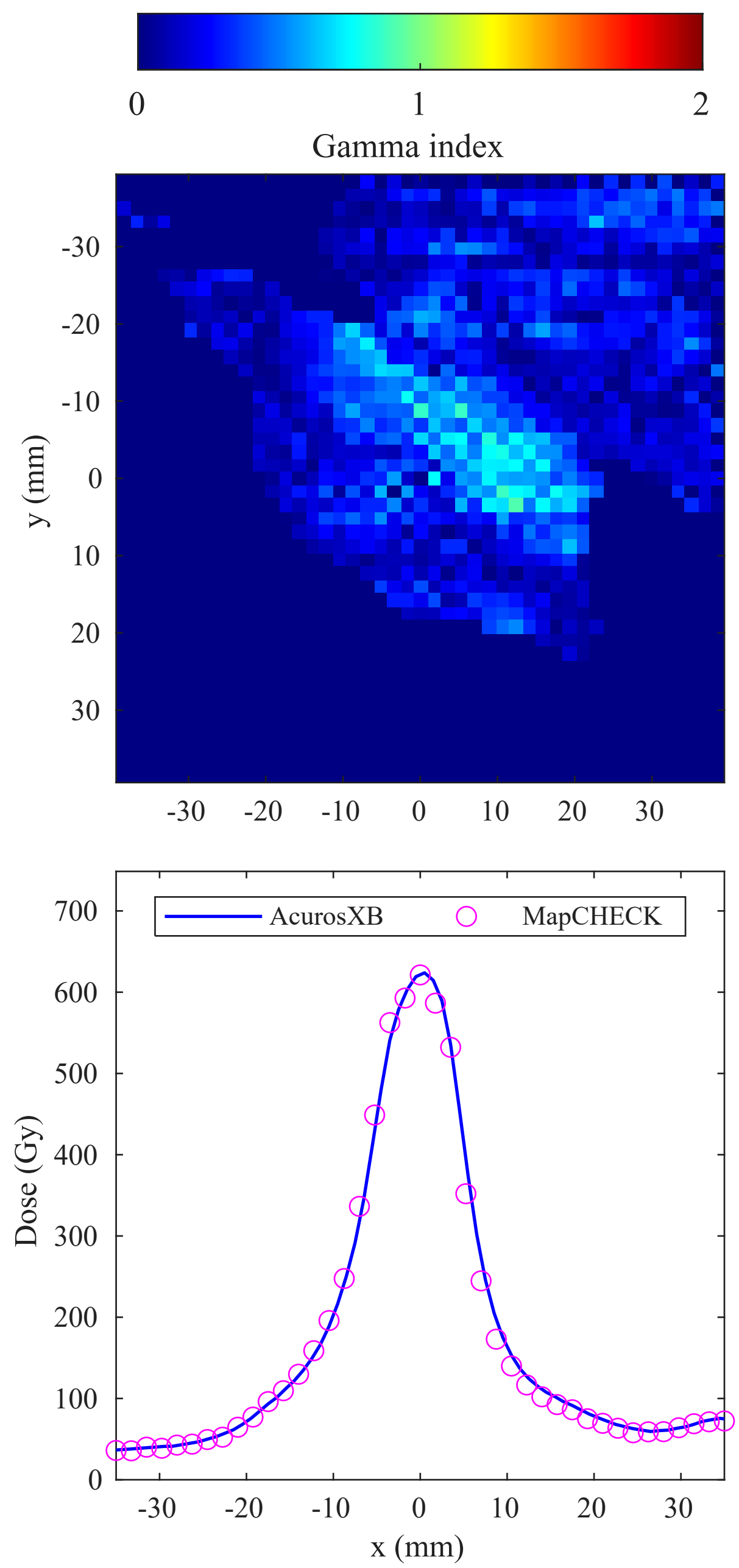

Figure S26. Measurement 24. Plan 16, 2 targets, equivalent diameter 26.7 mm. SRS MapCHECK fraction passing gamma 2%/1 mm = 96.7%

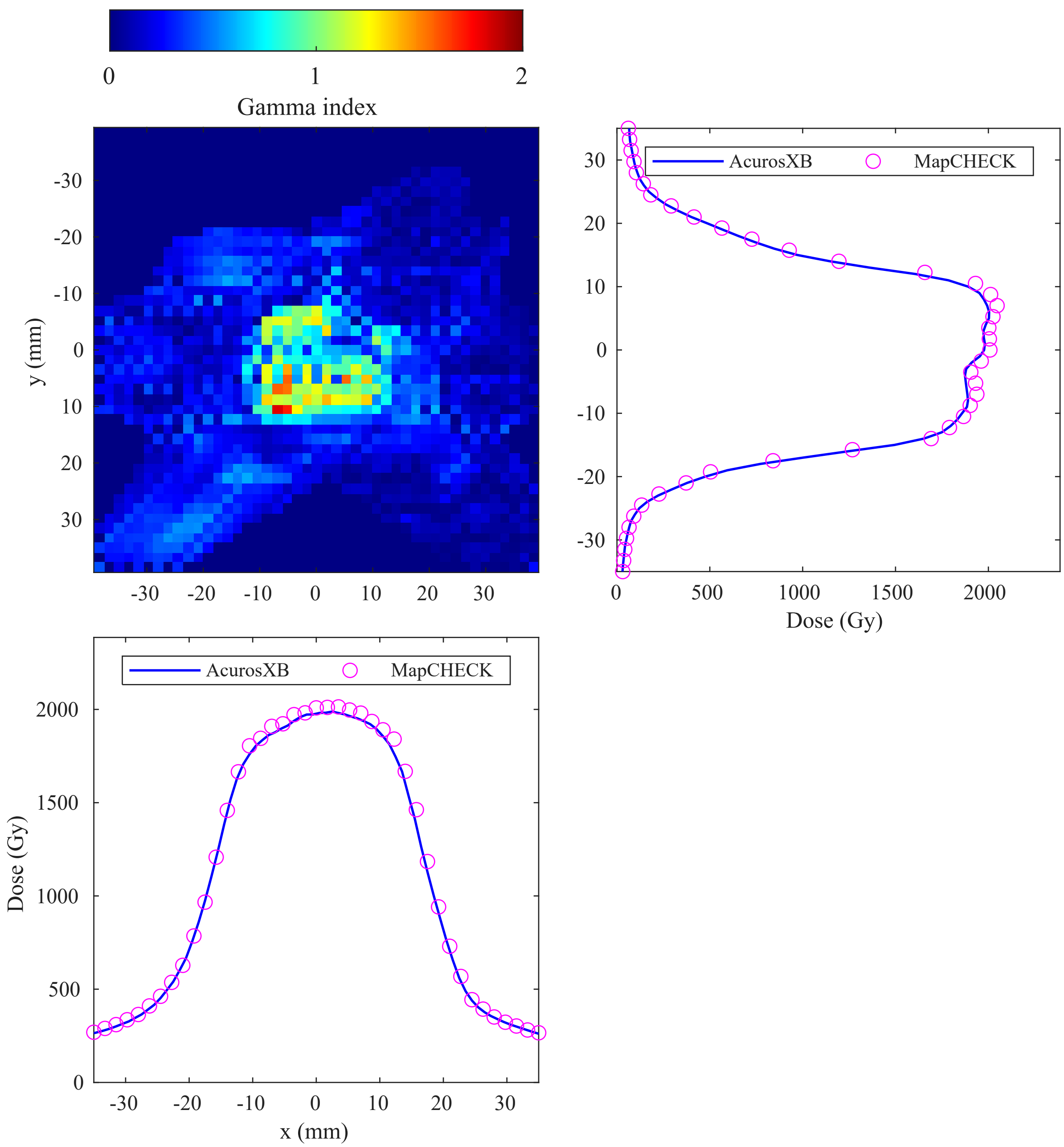

Figure S27. Measurement 25. Plan 16, 2 targets, equivalent diameter 3.3 mm. SRS MapCHECK fraction passing gamma 2%/1 mm = 100.0%

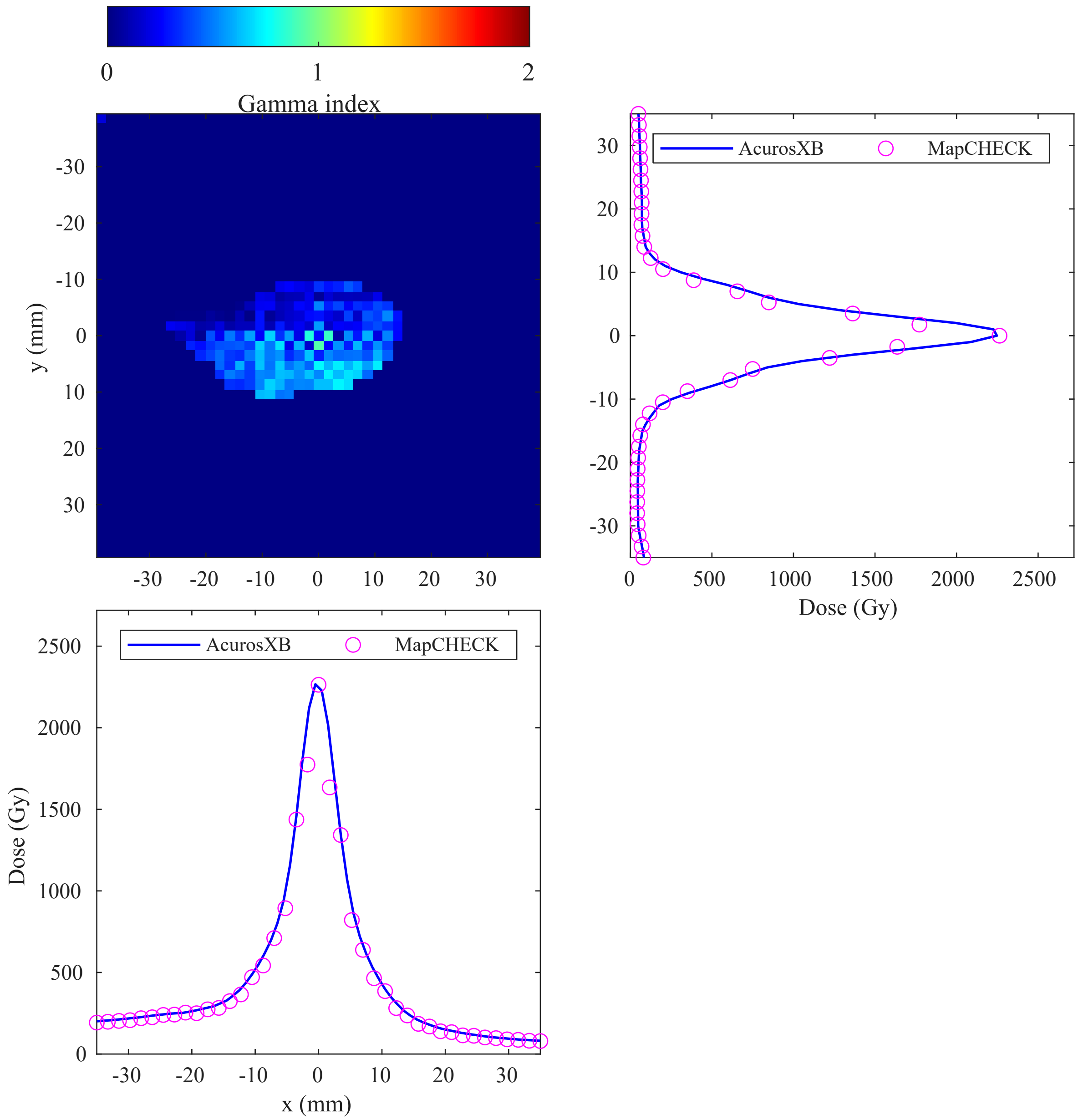

Figure S28. Measurement 26. Plan 17, single target, equivalent diameter 20.0 mm. SRS MapCHECK fraction passing gamma 2%/1 mm = 93.0%

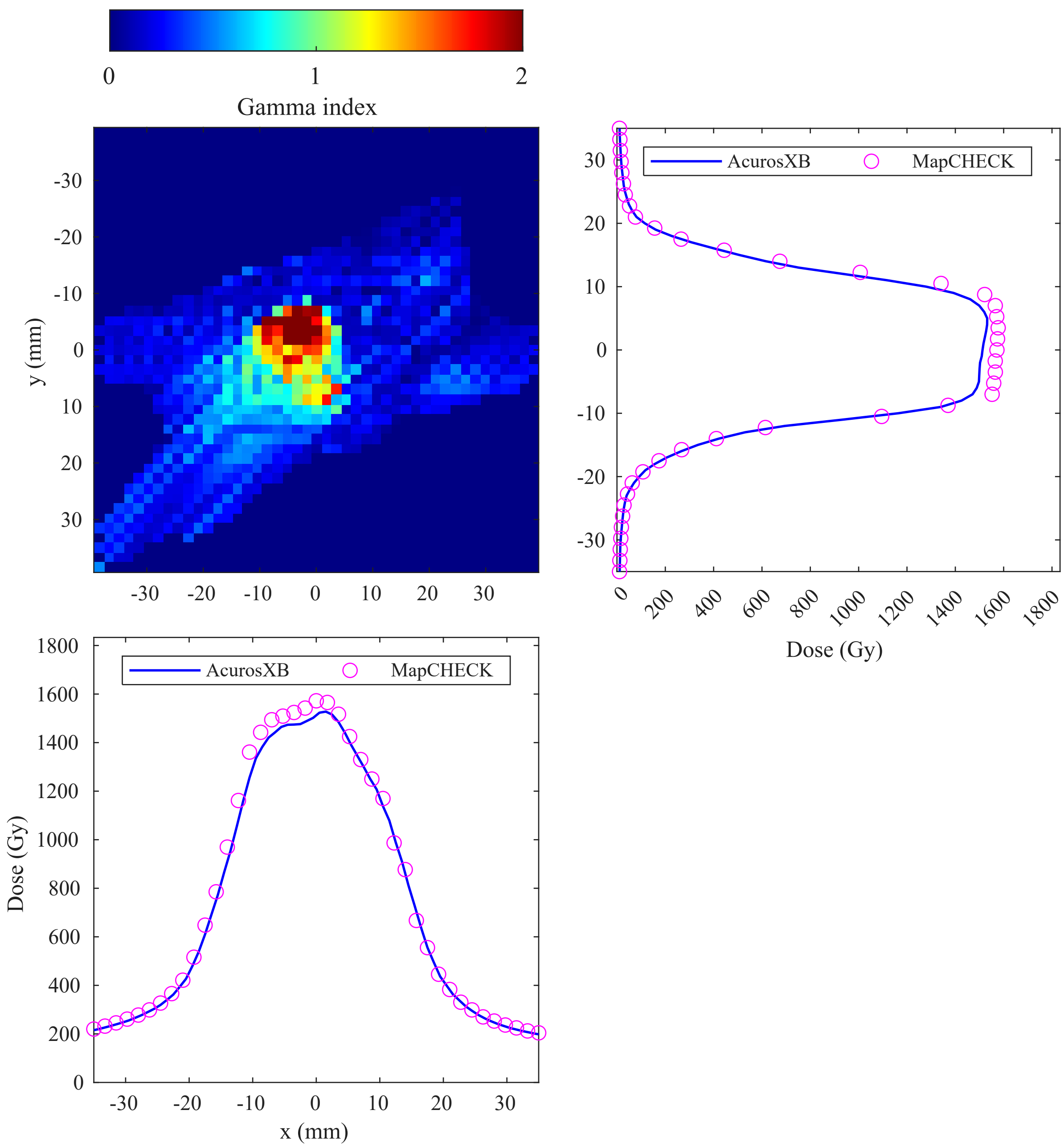

Figure S29. Measurement 27. Plan 18, single target, equivalent diameter 16.2 mm. SRS MapCHECK fraction passing gamma 2%/1 mm = 94.3%

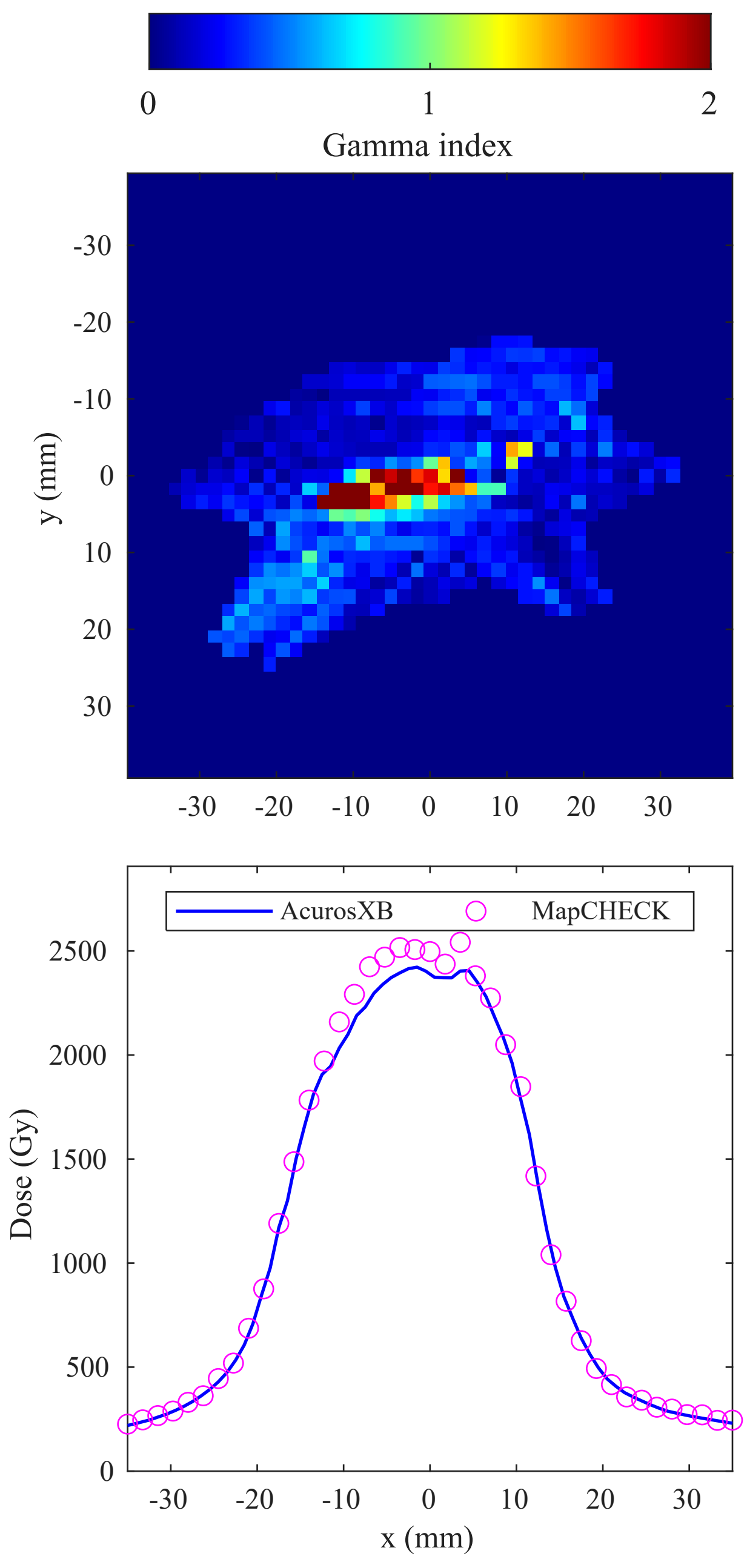

Figure S30. Measurement 28. Plan 19, single target, equivalent diameter 31.7 mm. SRS MapCHECK fraction passing gamma 2%/1 mm = 87.6%

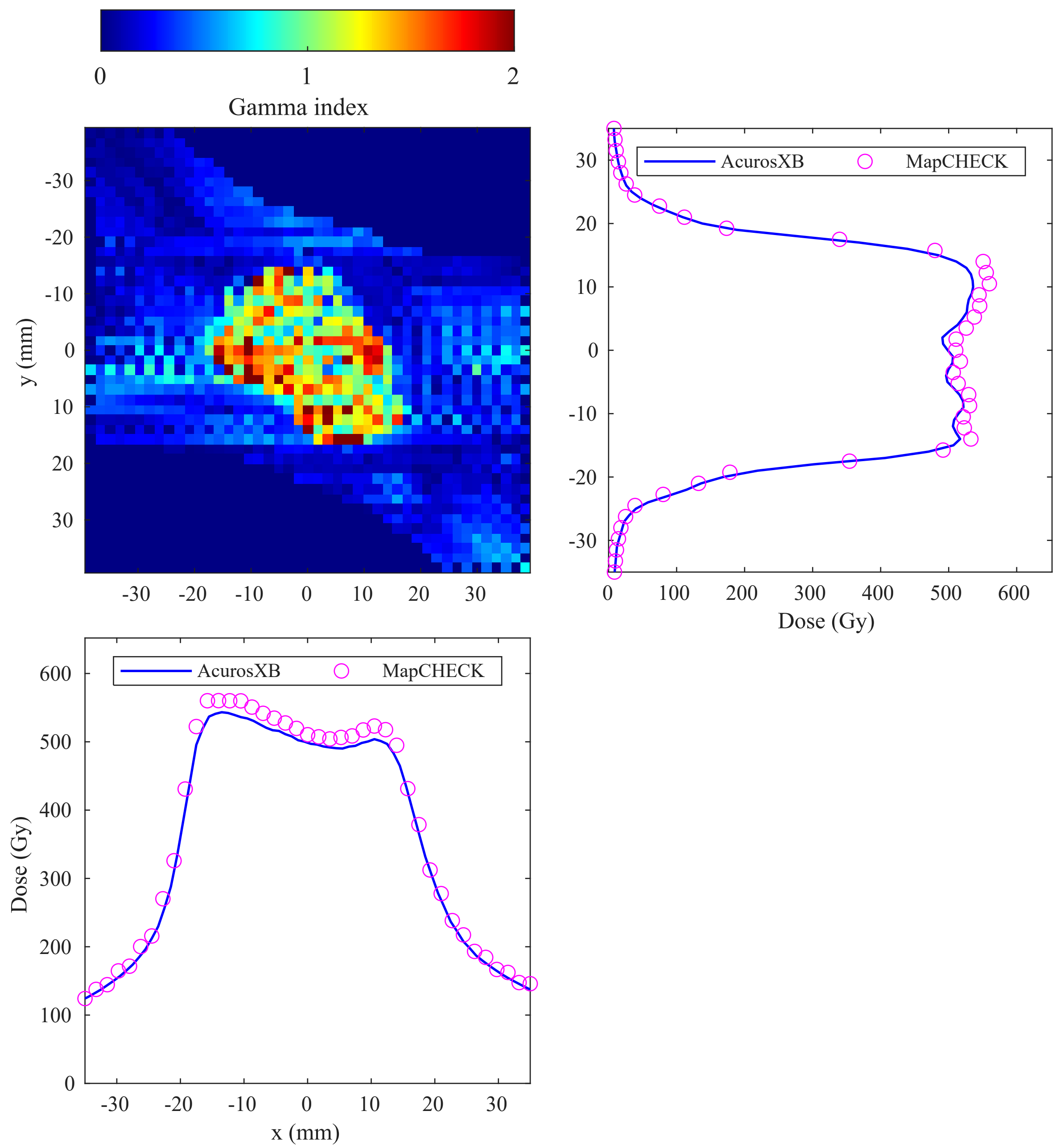

Figure S31. Measurement 29. Plan 20, single target, equivalent diameter 19.9 mm. SRS MapCHECK fraction passing gamma 2%/1 mm = 94.8%

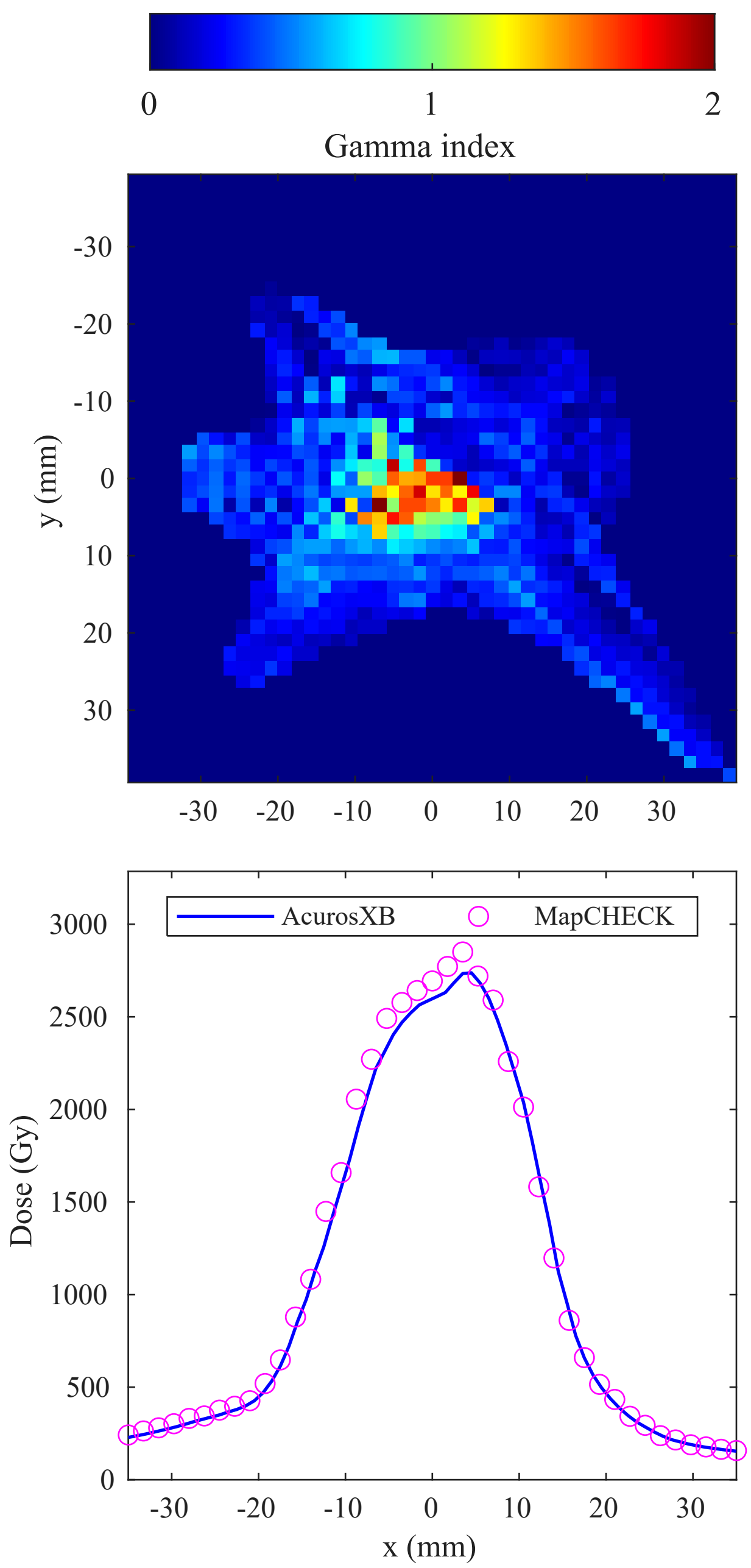

Figure S32. Measurement 30. Plan 21, 6 targets, equivalent diameter 8.7 mm. SRS MapCHECK fraction passing gamma 2%/1 mm = 99.5%

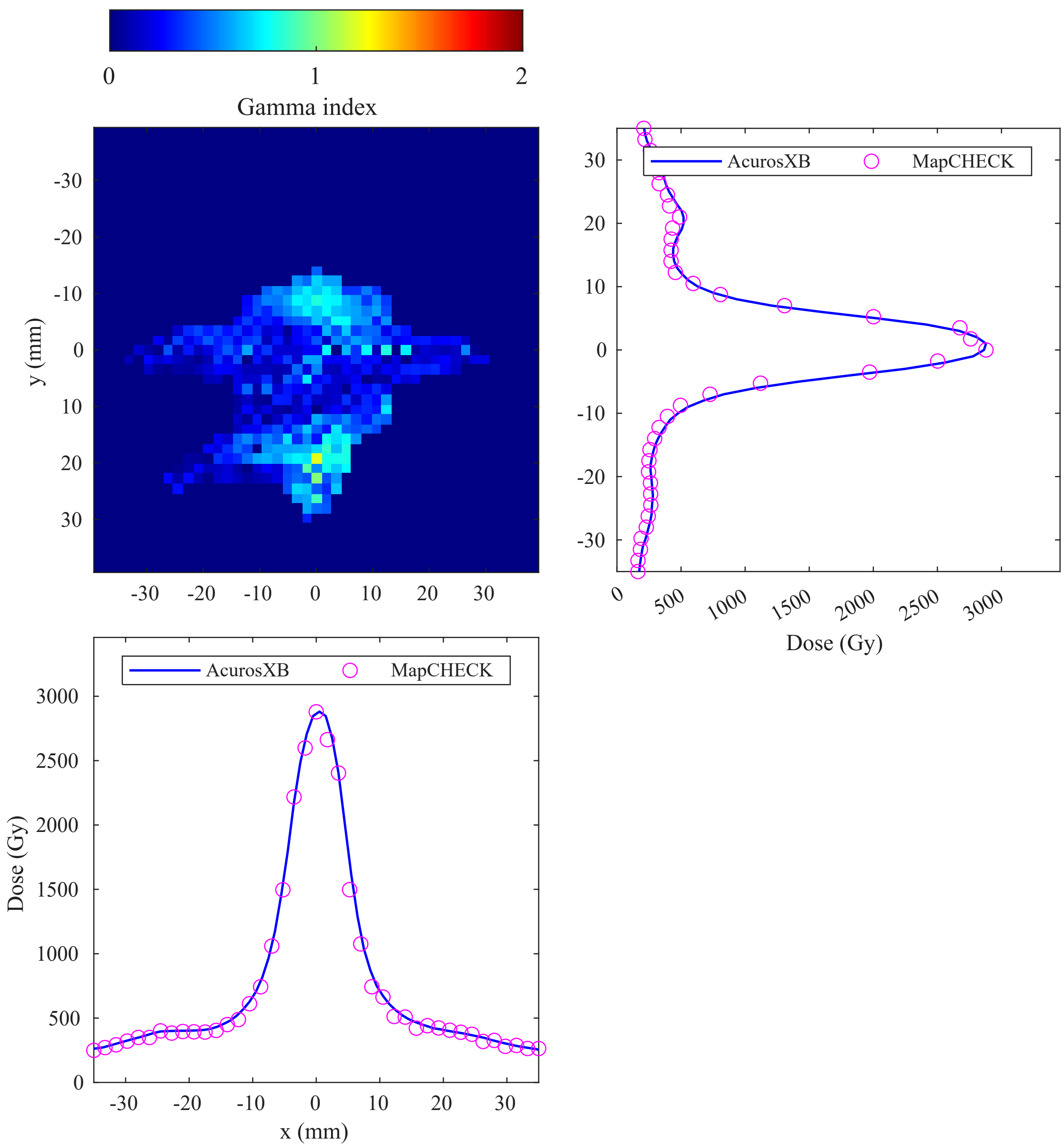

Figure S33. Measurement 31. Plan 21, 6 targets, equivalent diameter 4.0 mm. SRS MapCHECK fraction passing gamma 2%/1 mm = 99.5%

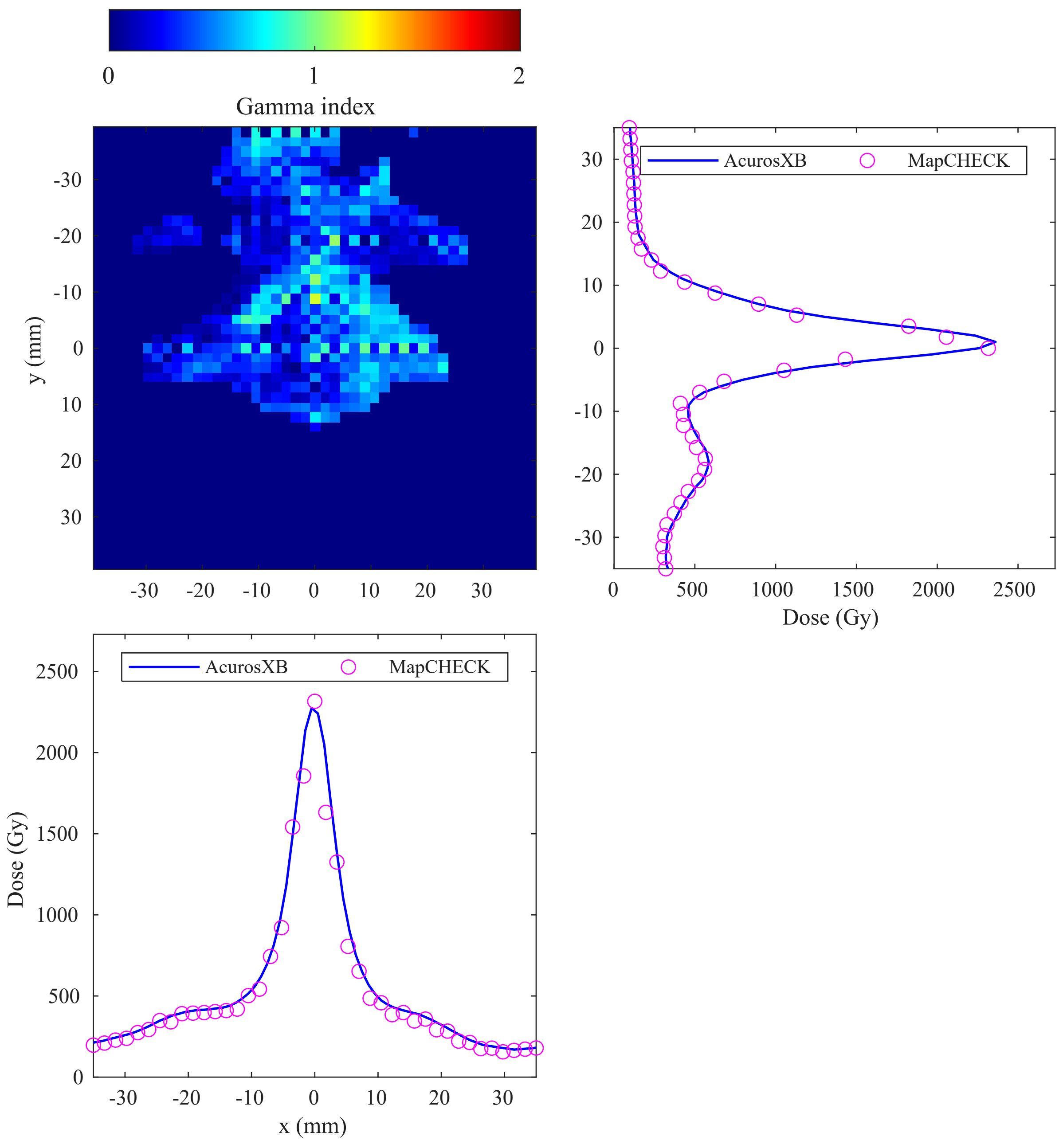

Figure S34. Measurement 32. Plan 22, 6 targets, equivalent diameter 6.6 mm. SRS MapCHECK fraction passing gamma 2%/1 mm = 98.0%

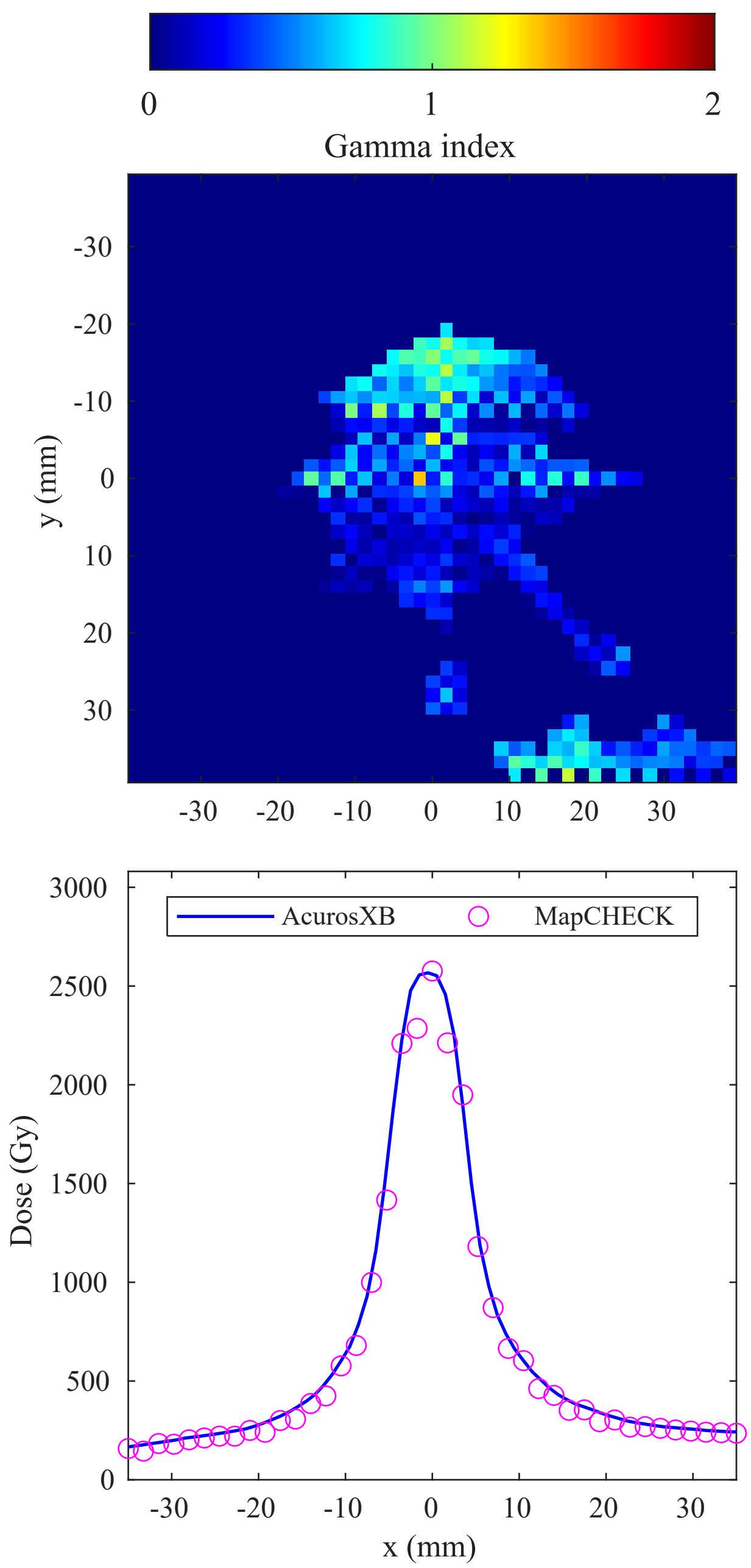

Figure S35. Measurement 33. Plan 22, 6 targets, equivalent diameter 2.7 mm. SRS MapCHECK fraction passing gamma 2%/1 mm = 97.0%

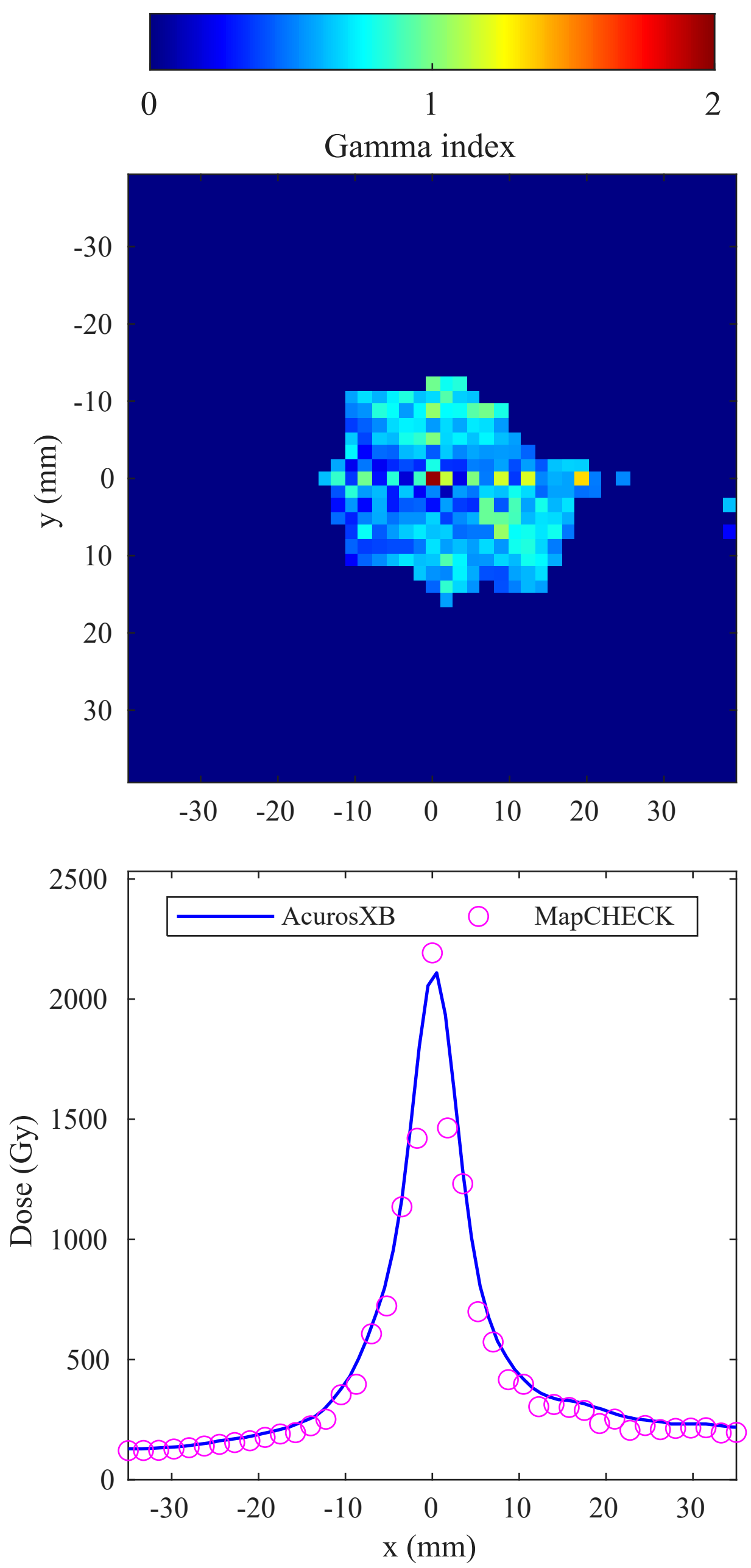

Figure S36. Measurement 34. Plan 23, single target, equivalent diameter 3.0 mm. SRS MapCHECK fraction passing gamma 2%/1 mm = 98.0%

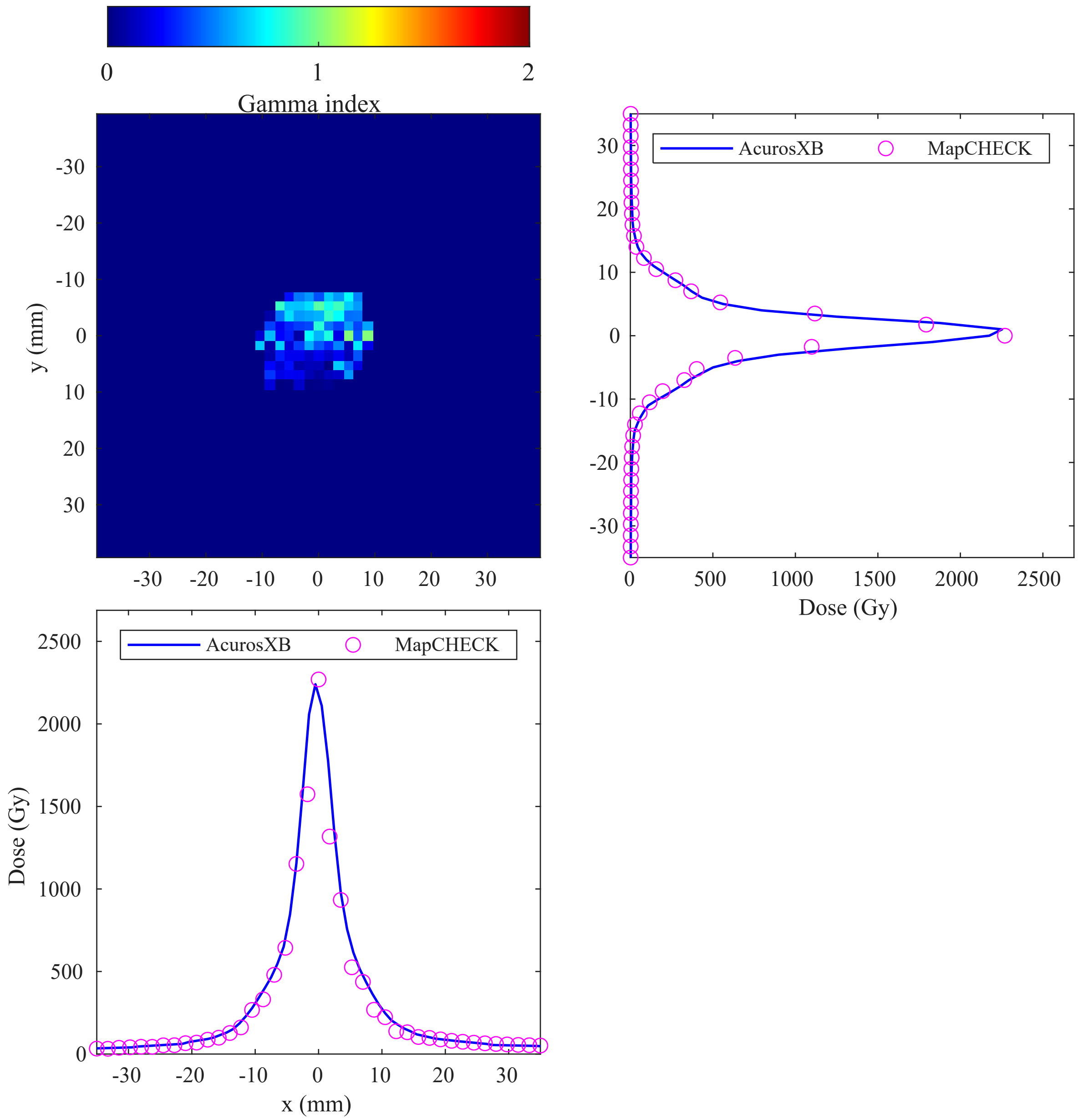

Figure S37. Measurement 35. Plan 24, 10 targets, equivalent diameter 3.8 mm. SRS MapCHECK fraction passing gamma 2%/1 mm = 88.3%

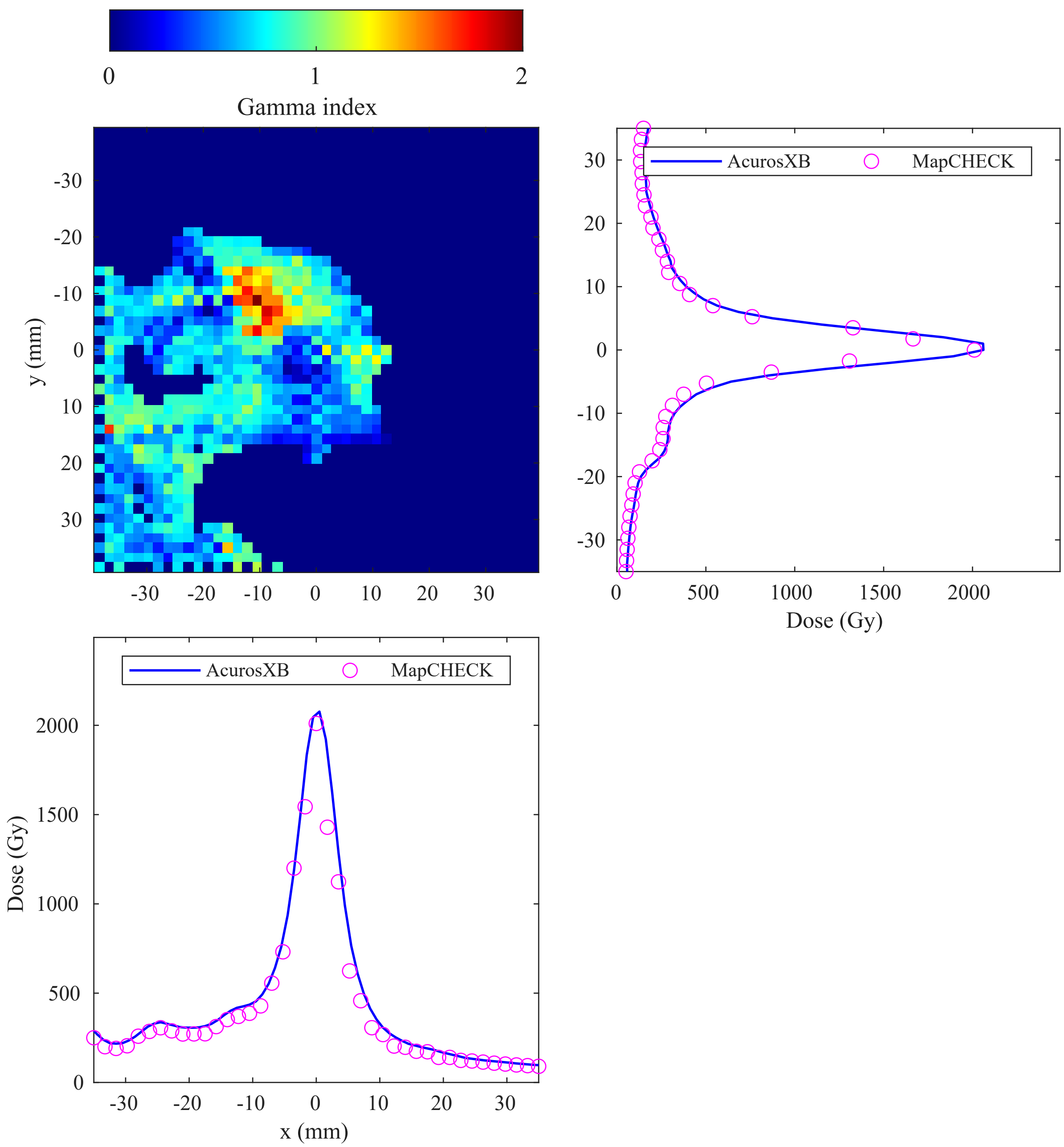

Figure S38. Measurement 36. Plan 24, 10 targets, equivalent diameter 2.4 mm. SRS MapCHECK fraction passing gamma 2%/1 mm = 97.3%

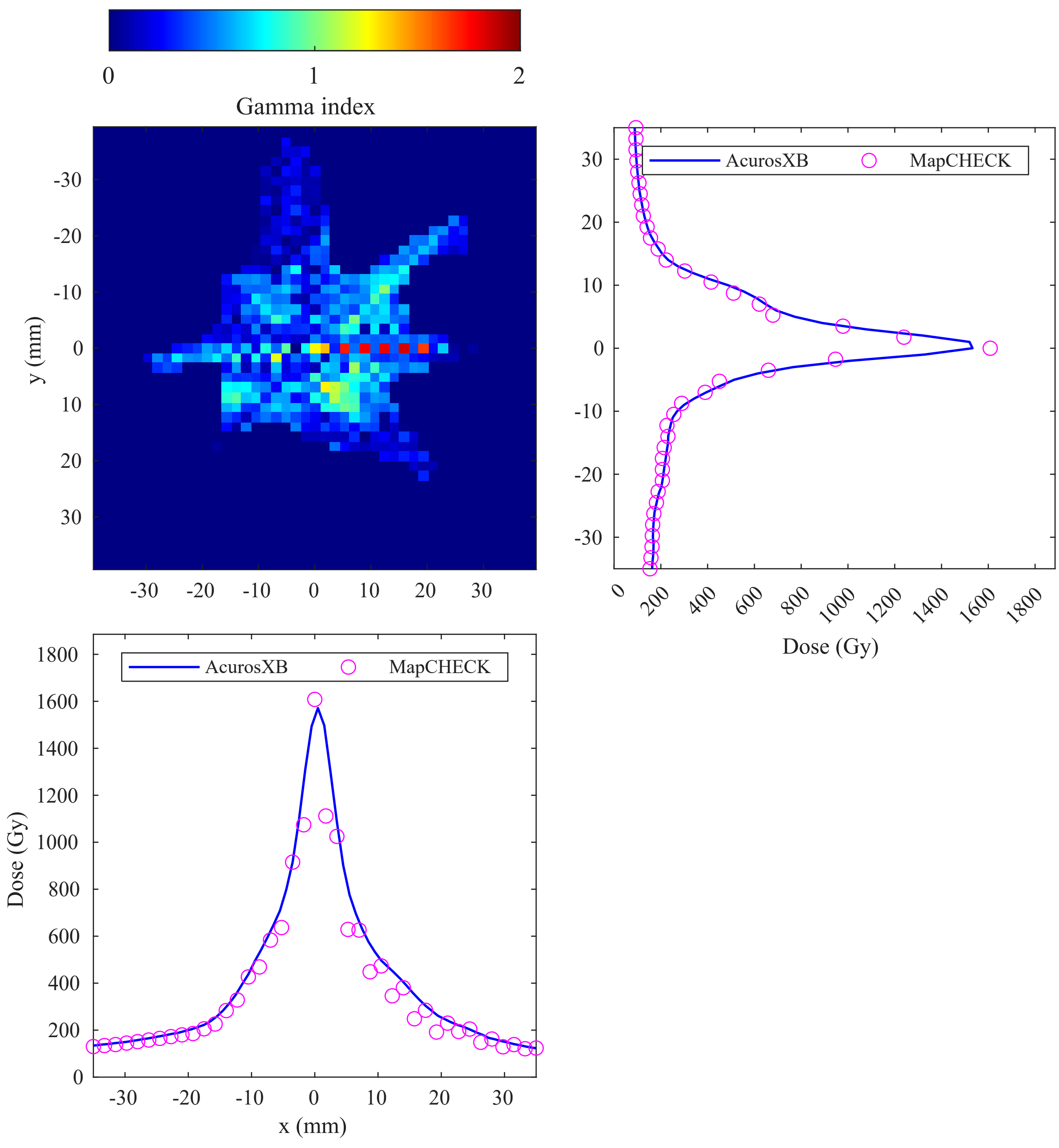

Figure S39. Measurement 37. Plan 25, 9 targets, equivalent diameter 4.2 mm. SRS MapCHECK fraction passing gamma 2%/1 mm = 98.2%

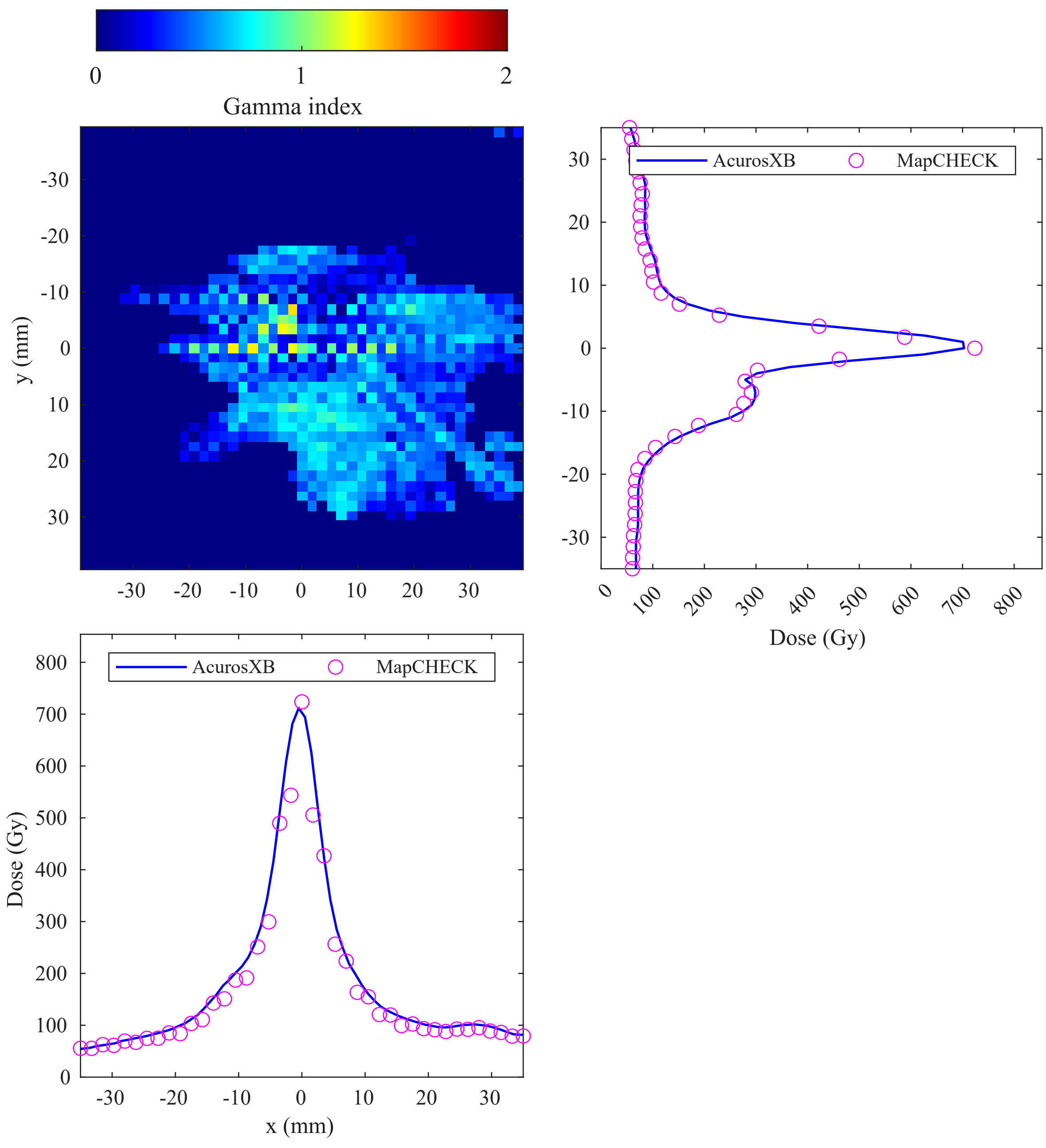

Figure S40. Measurement 38. Plan 25, 9 targets, equivalent diameter 17.1 mm. SRS MapCHECK fraction passing gamma 2%/1 mm = 99.9%

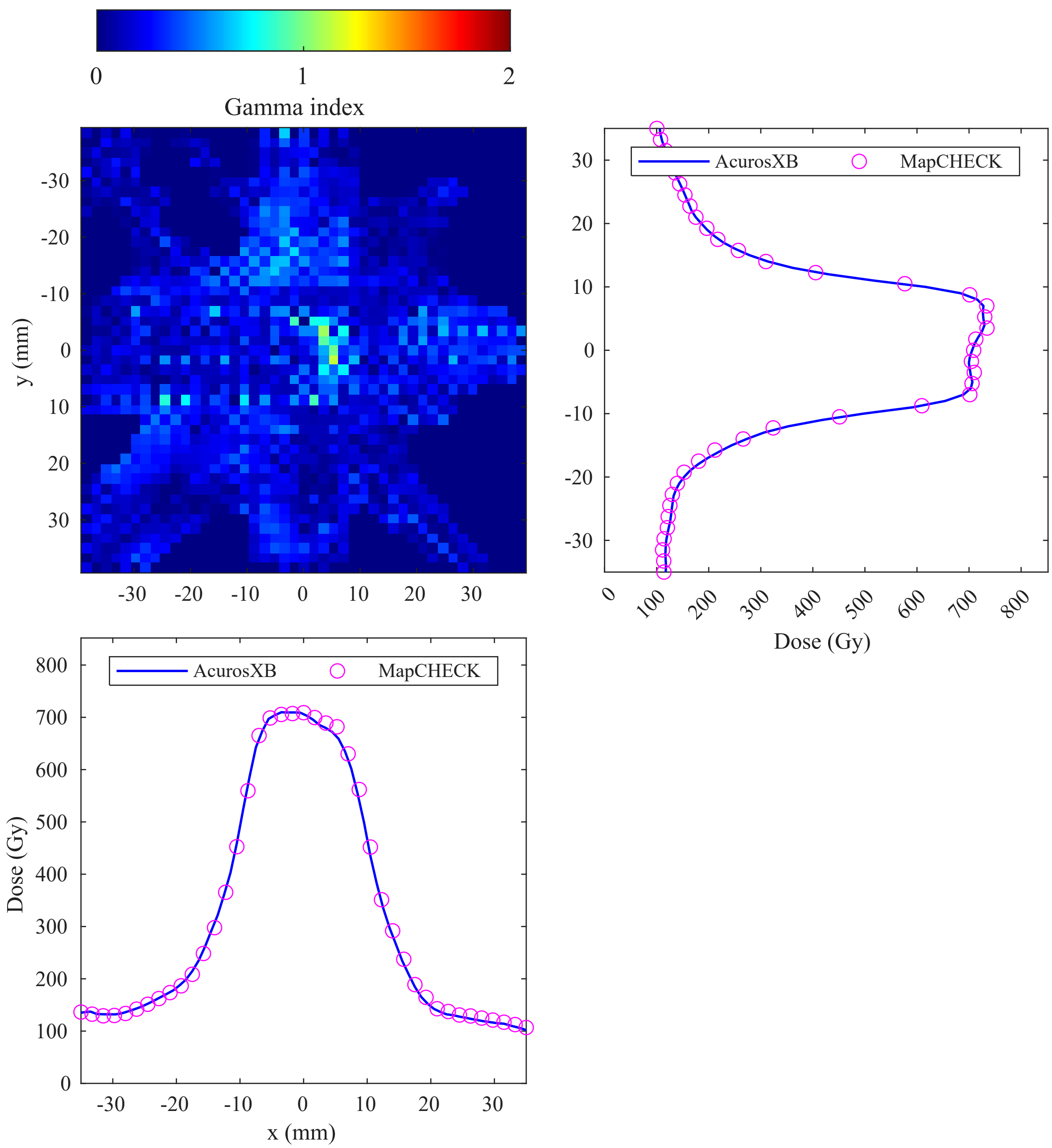

Figure S41. Measurement 39. Plan 26, single target, equivalent diameter 30.9 mm. SRS MapCHECK fraction passing gamma 2%/1 mm = 82.5%

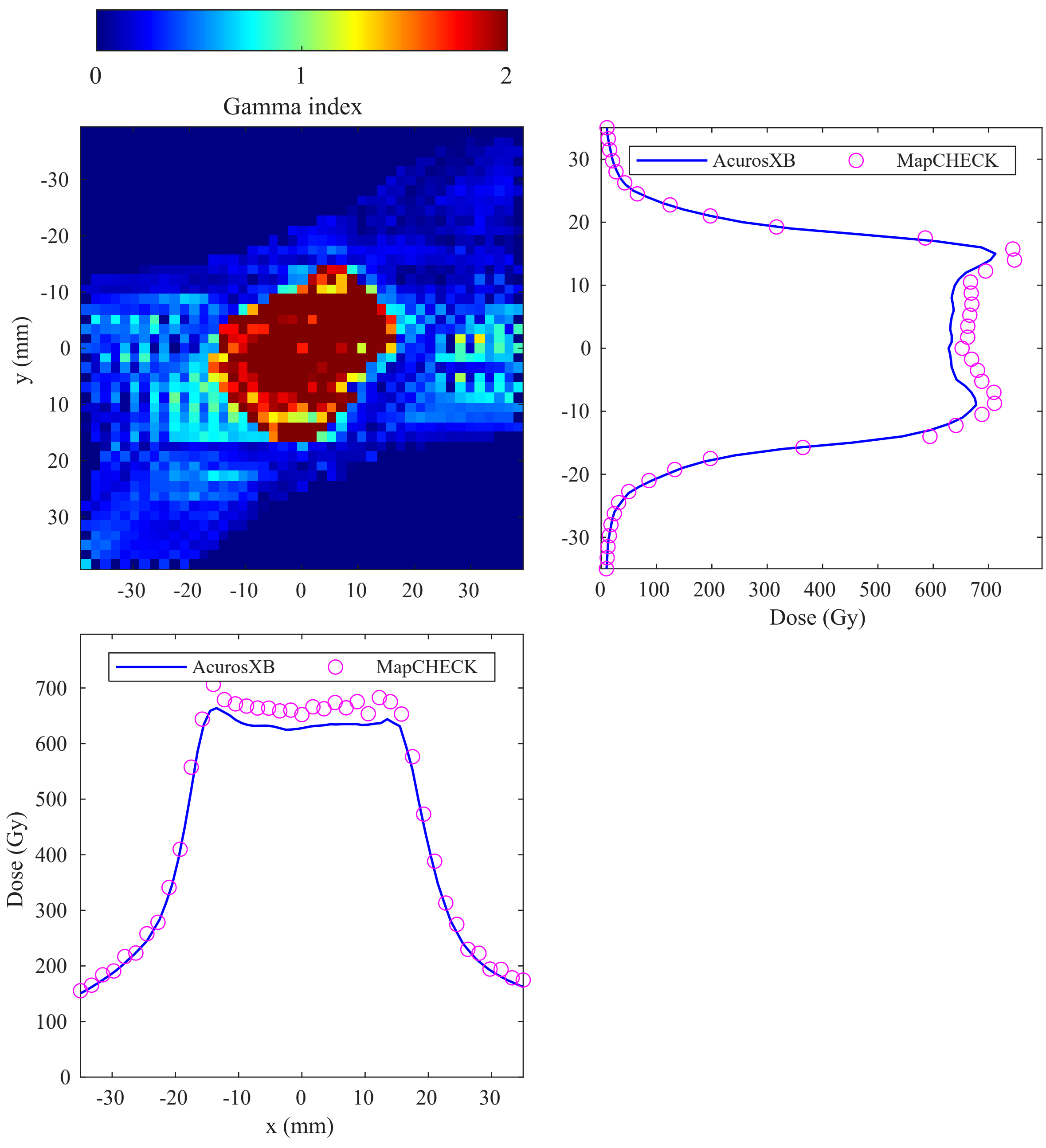

Figure S42. Measurement 40. Plan 27, 4 targets, equivalent diameter 23.4 mm. SRS MapCHECK fraction passing gamma 2%/1 mm = 98.0%

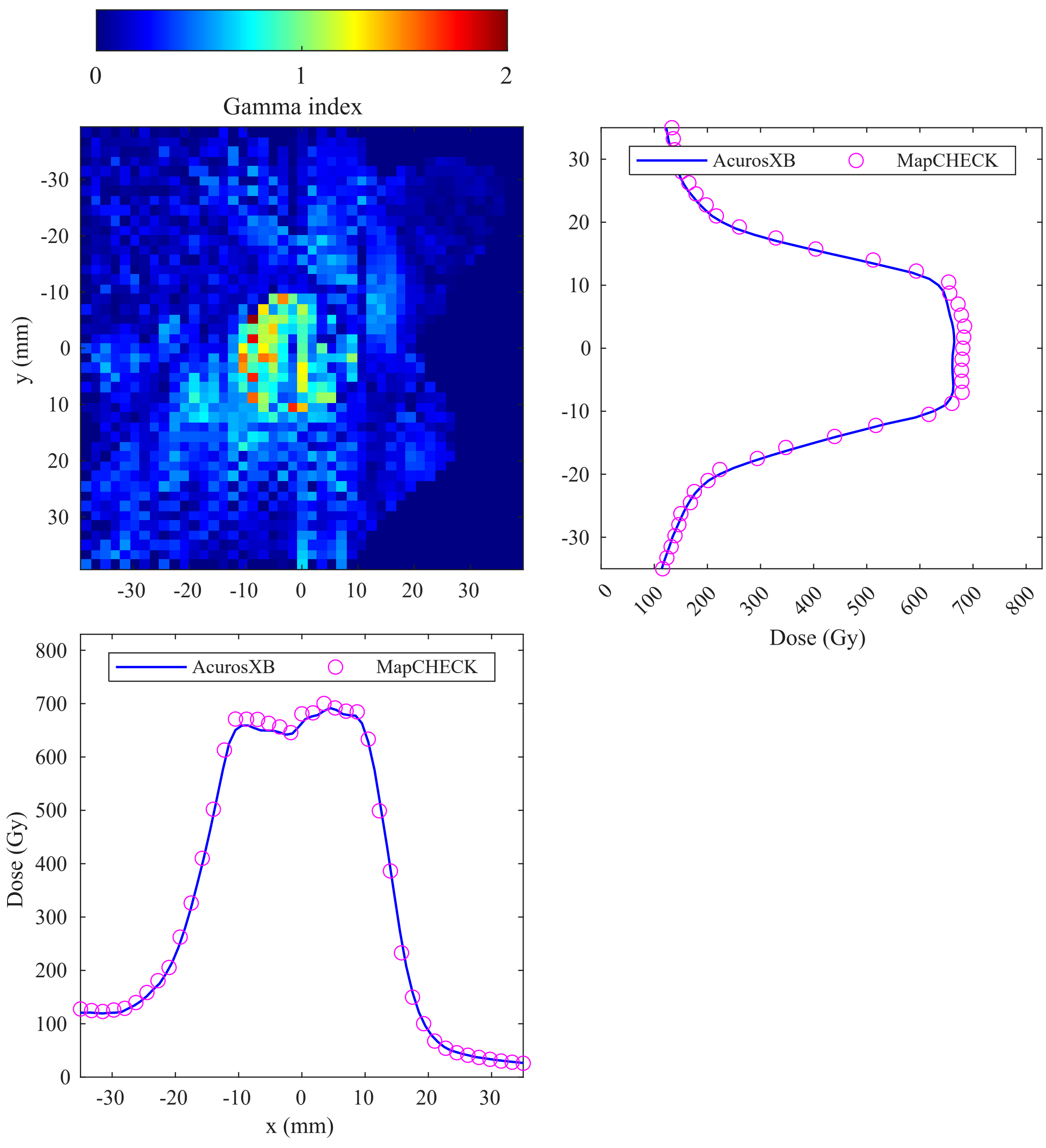

Figure S43. Measurement 41. Plan 27, 4 targets, equivalent diameter 13.3 mm. SRS MapCHECK fraction passing gamma 2%/1 mm = 99.7%

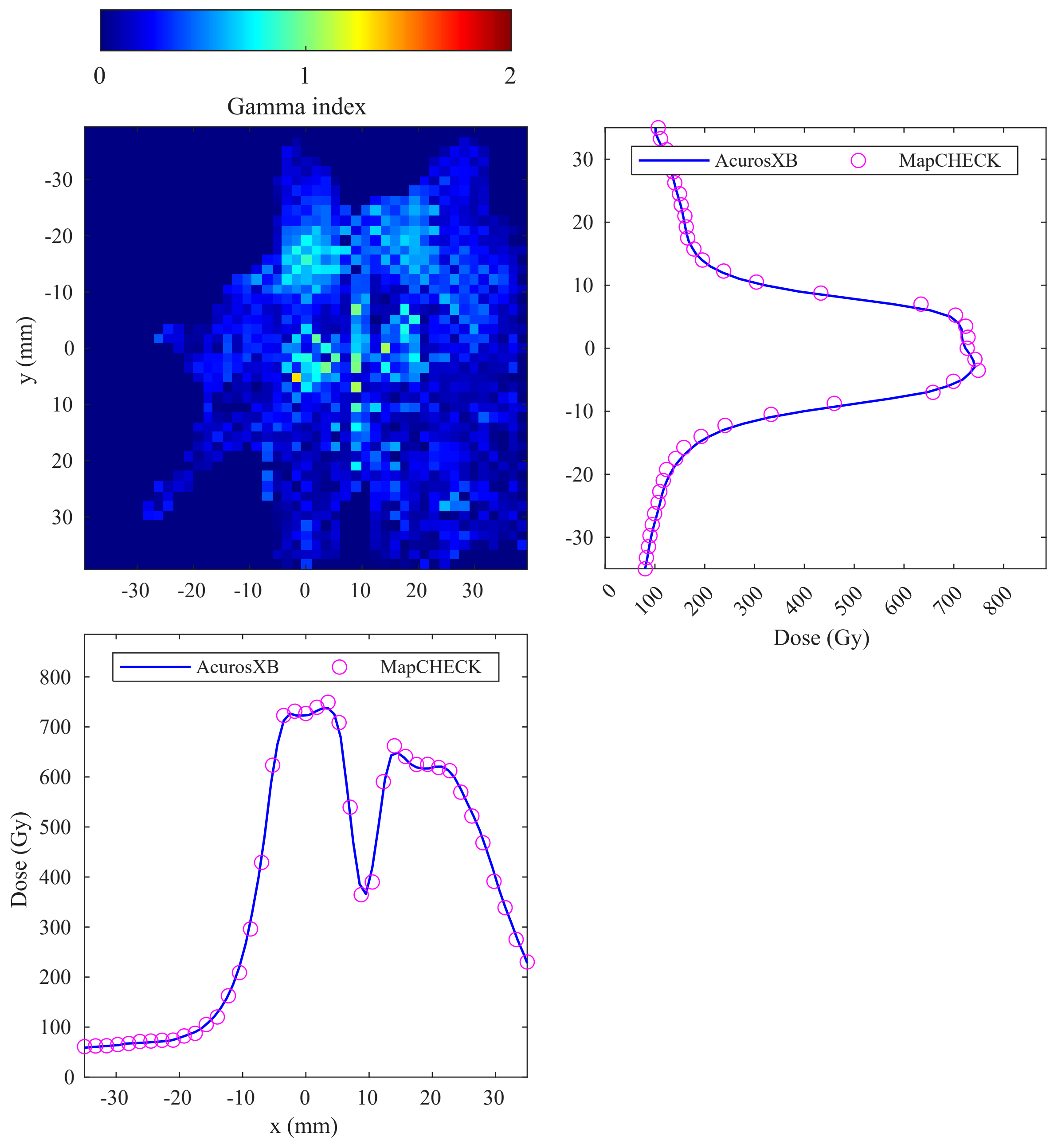

Figure S44. Measurement 42. Plan 28, single target, equivalent diameter 12.2 mm. SRS MapCHECK fraction passing gamma 2%/1 mm = 95.5%

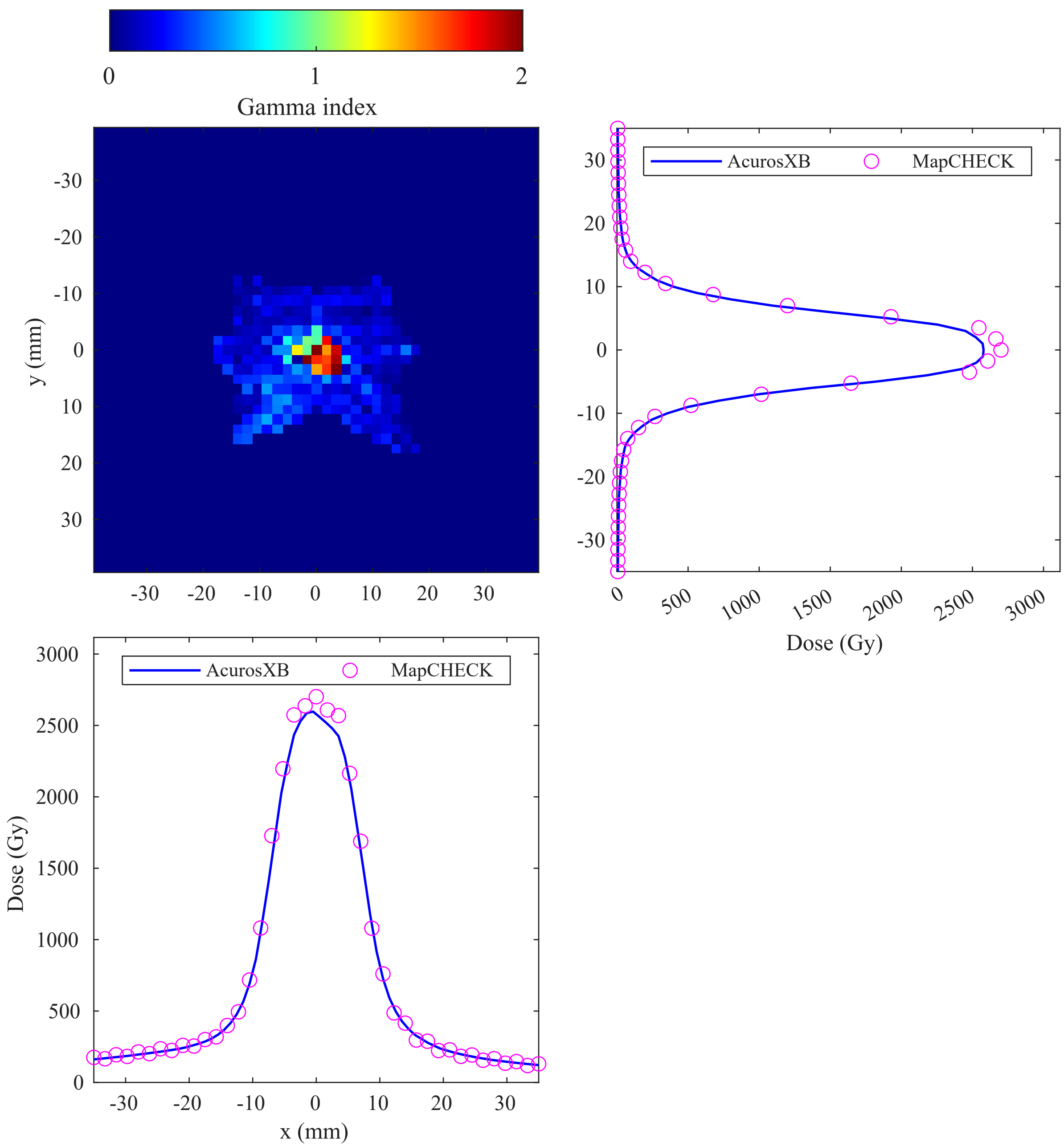

Figure S45. Measurement 43. Plan 29, 7 targets, equivalent diameter 11.1 mm. SRS MapCHECK fraction passing gamma 2%/1 mm = 99.4%

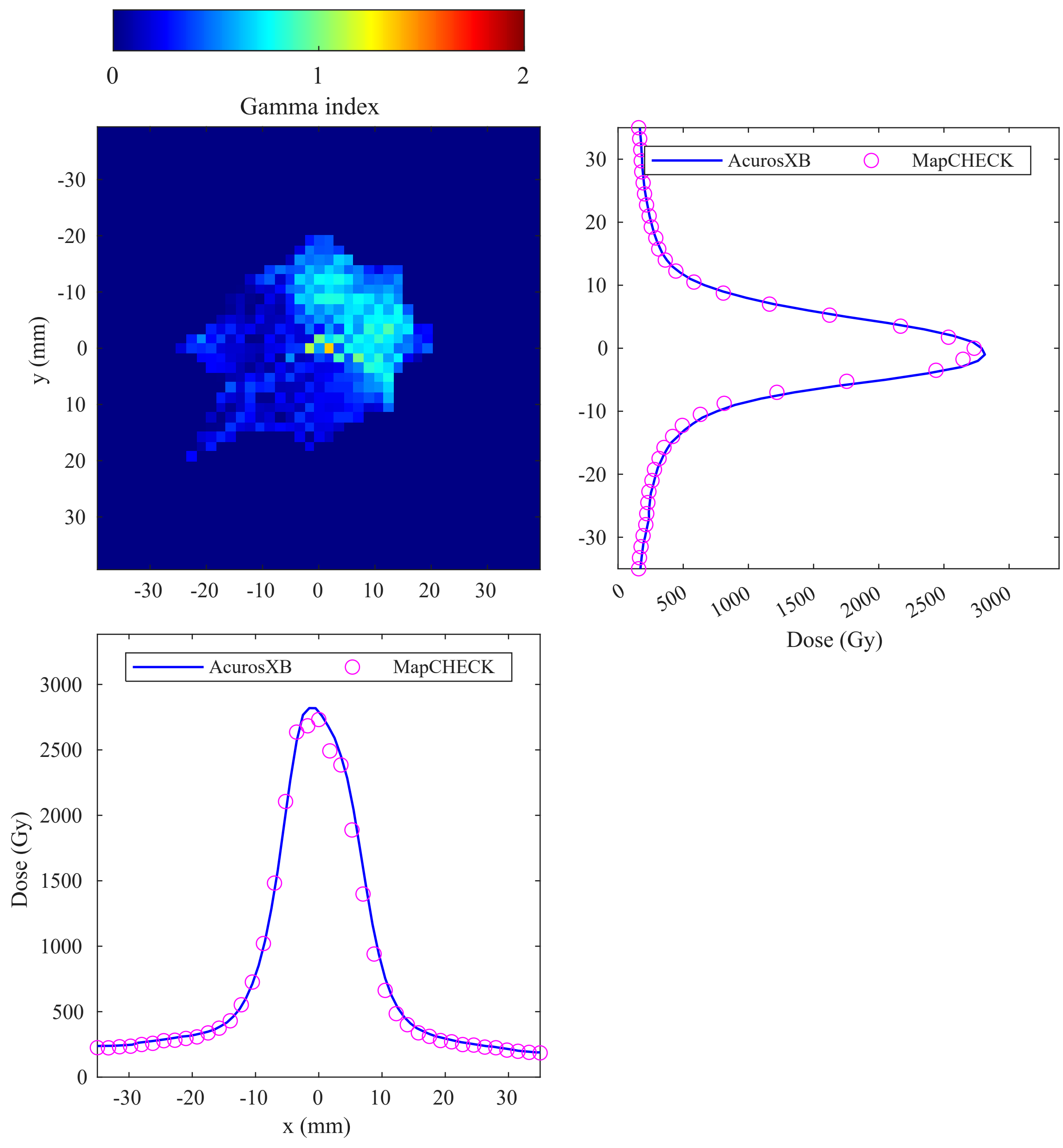

Figure S46. Measurement 44. Plan 29, 7 targets, equivalent diameter 3.9 mm. SRS MapCHECK fraction passing gamma 2%/1 mm = 99.6%

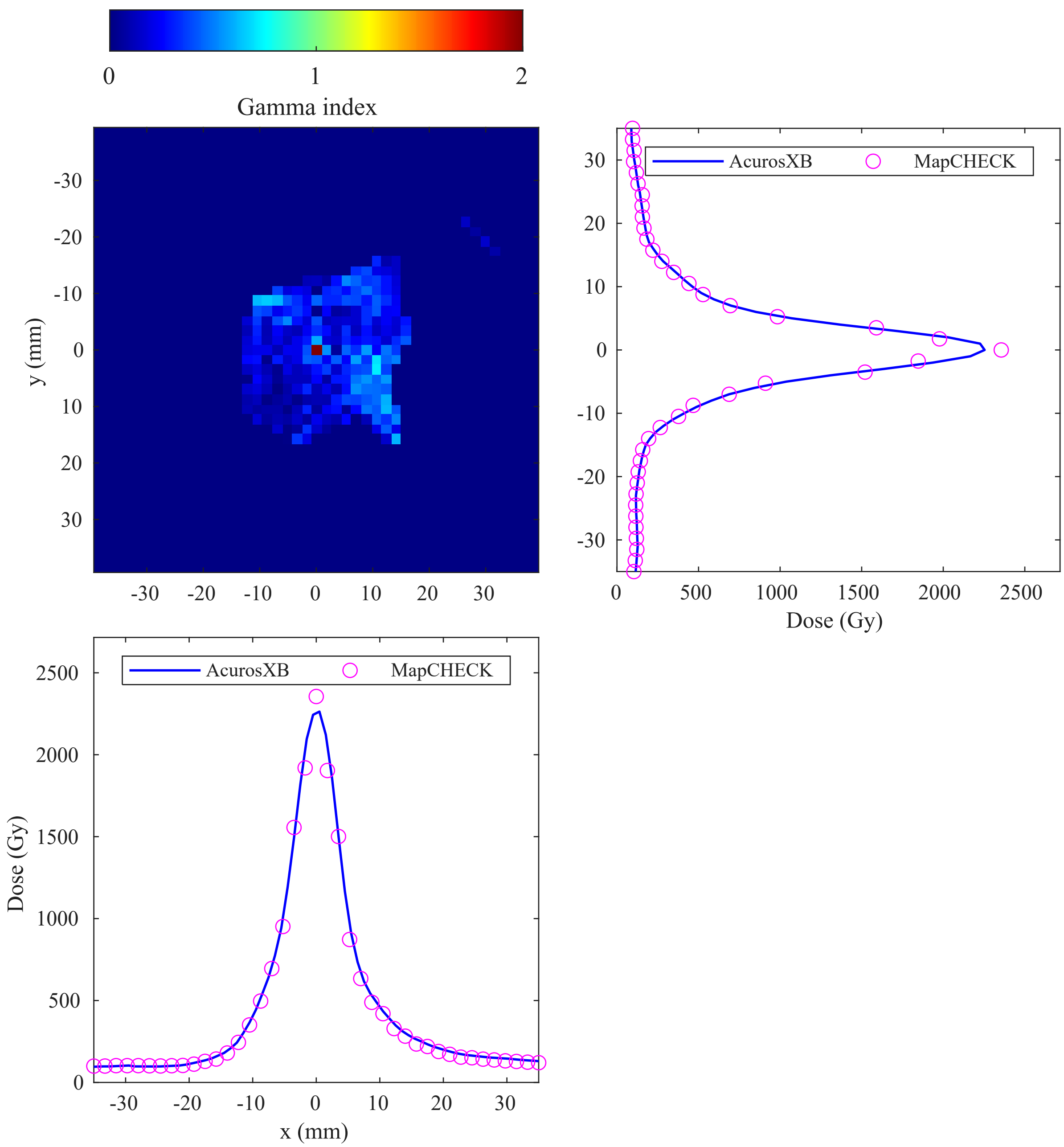

Figure S47. Measurement 45. Plan 30, 2 targets, equivalent diameter 4.6 mm. SRS MapCHECK fraction passing gamma 2%/1 mm = 99.3%

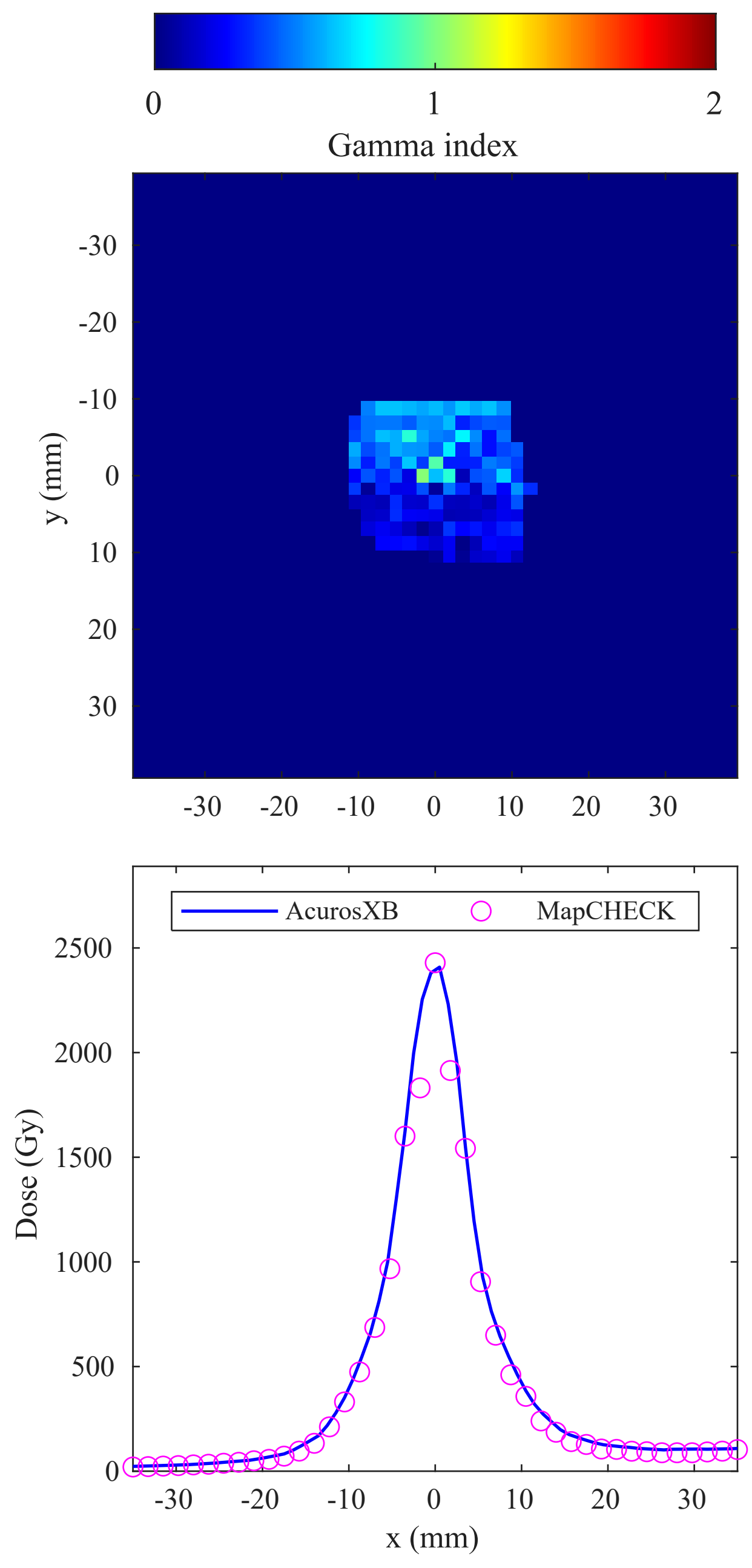

Figure S48. Measurement 46. Plan 30, 2 targets, equivalent diameter 5.9 mm. SRS MapCHECK fraction passing gamma 2%/1 mm = 99.4%

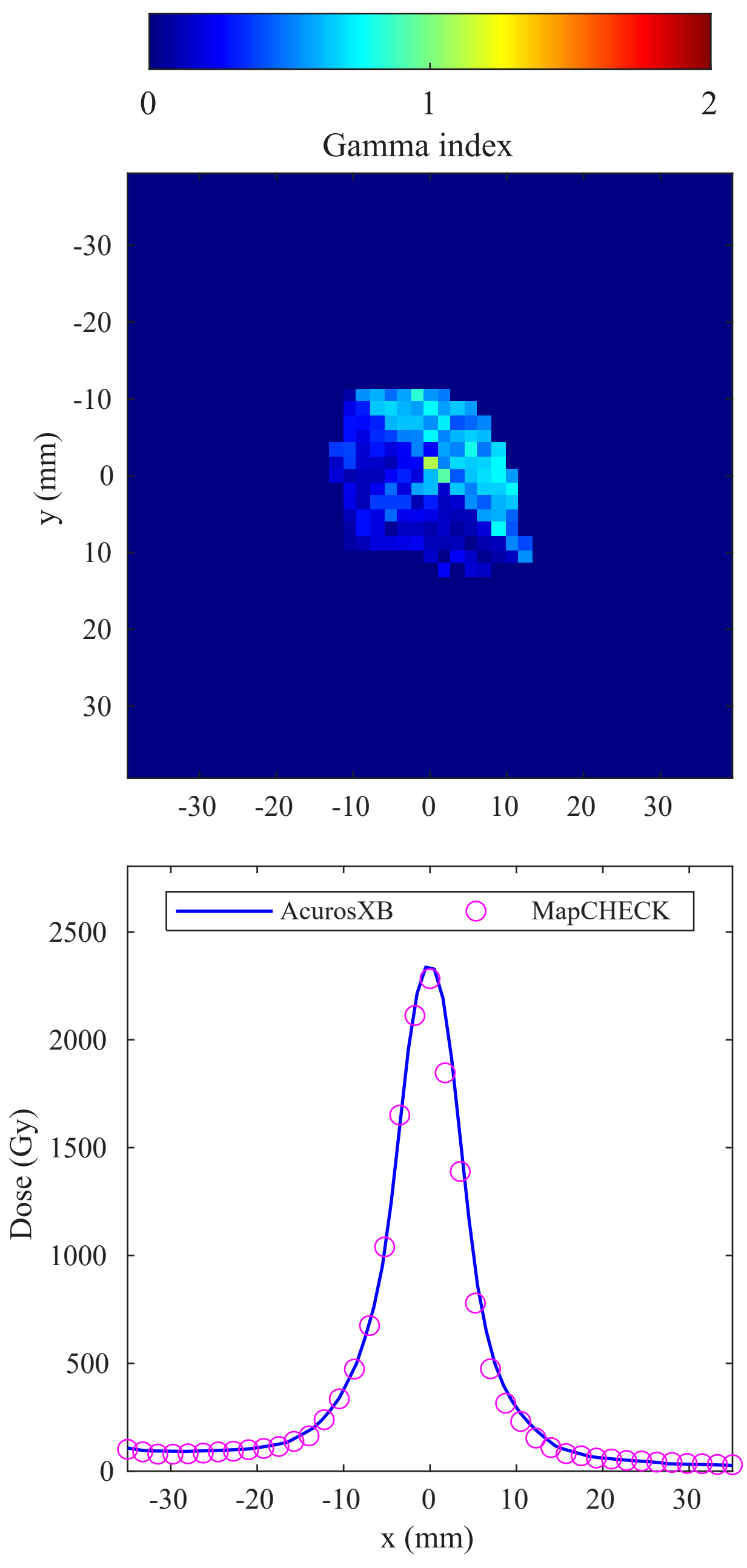

Figure S49. Measurement 47. Plan 31, 2 targets, equivalent diameter 5.5 mm. SRS MapCHECK fraction passing gamma 2%/1 mm = 100.0%

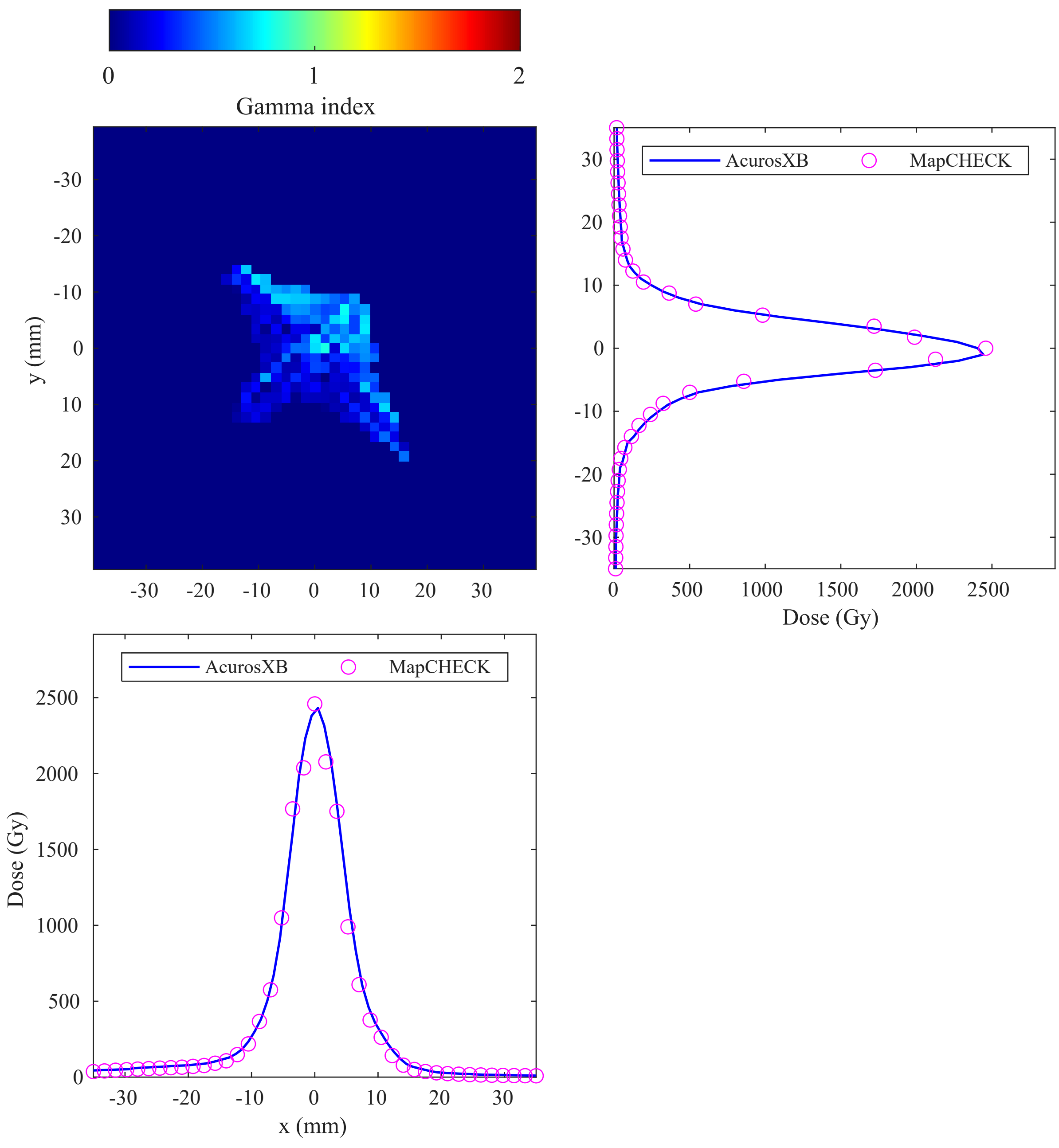

Figure S50. Measurement 48. Plan 31, 2 targets, equivalent diameter 2.4 mm. SRS MapCHECK fraction passing gamma 2%/1 mm = 98.2%

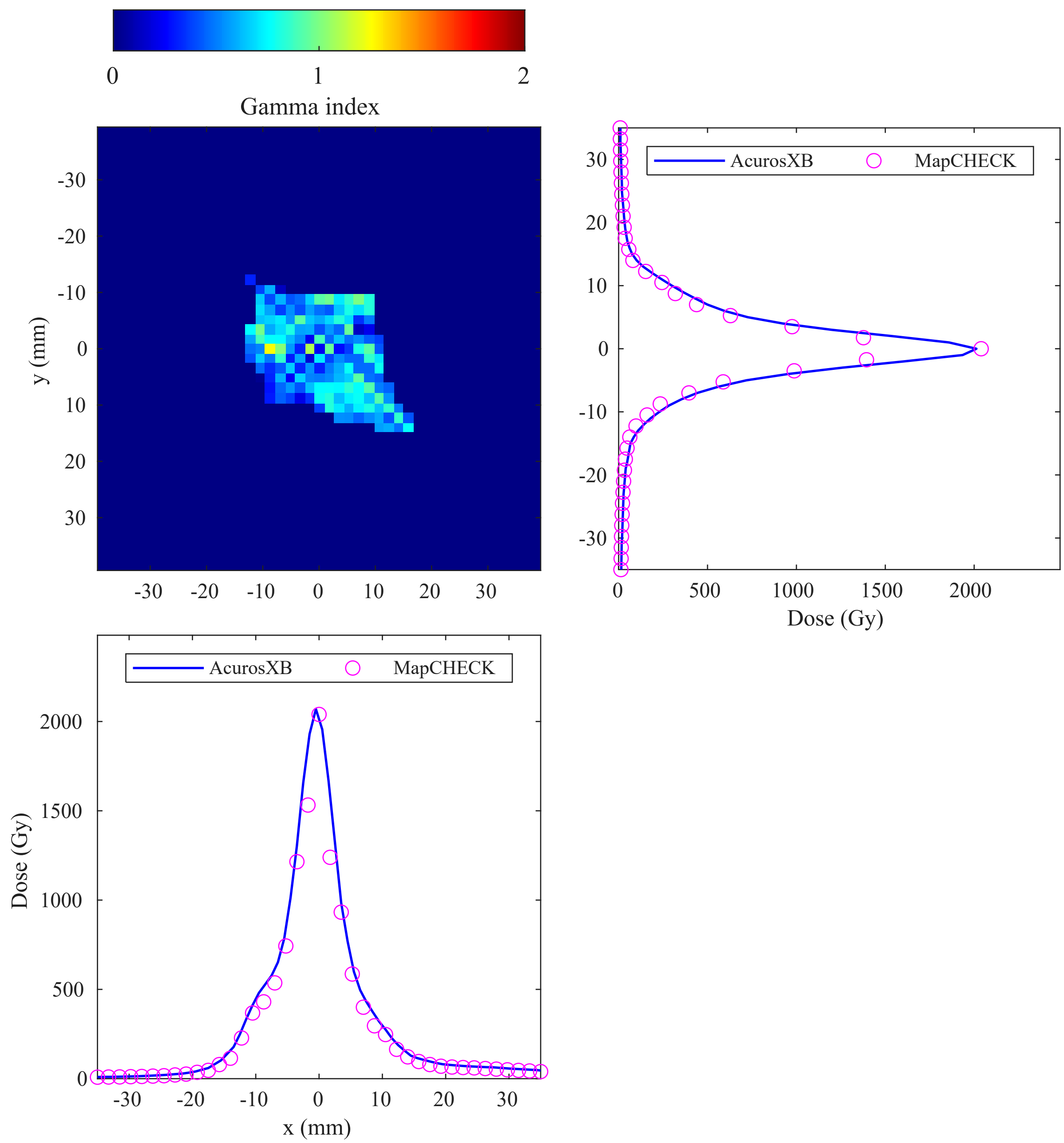

Figure S51. Measurement 49. Plan 32, single target, equivalent diameter 10.4 mm. SRS MapCHECK fraction passing gamma 2%/1 mm = 94.9%

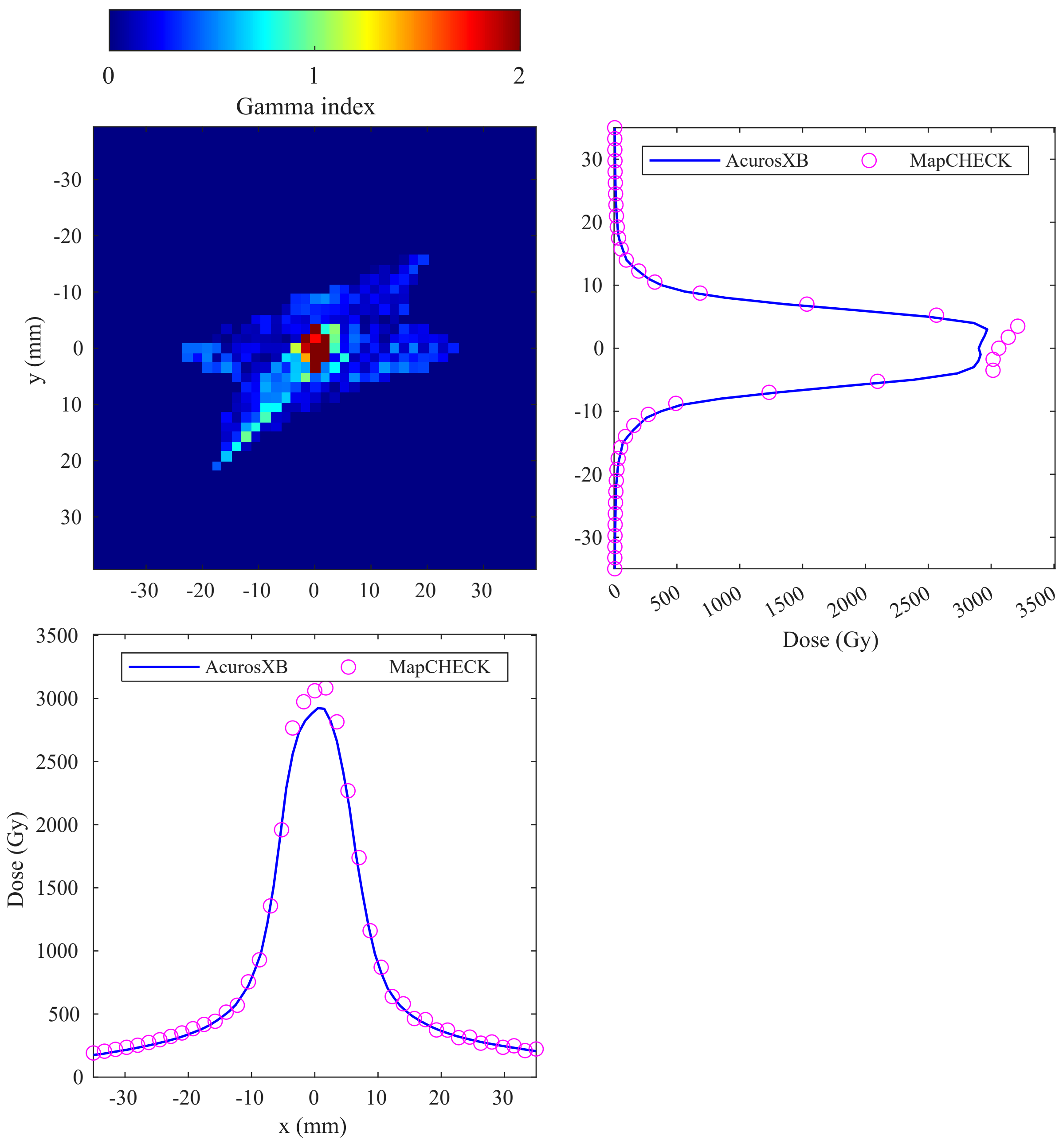

Figure S52. Measurement 50. Plan 33, single target, equivalent diameter 23.2 mm. SRS MapCHECK fraction passing gamma 2%/1 mm = 97.6%

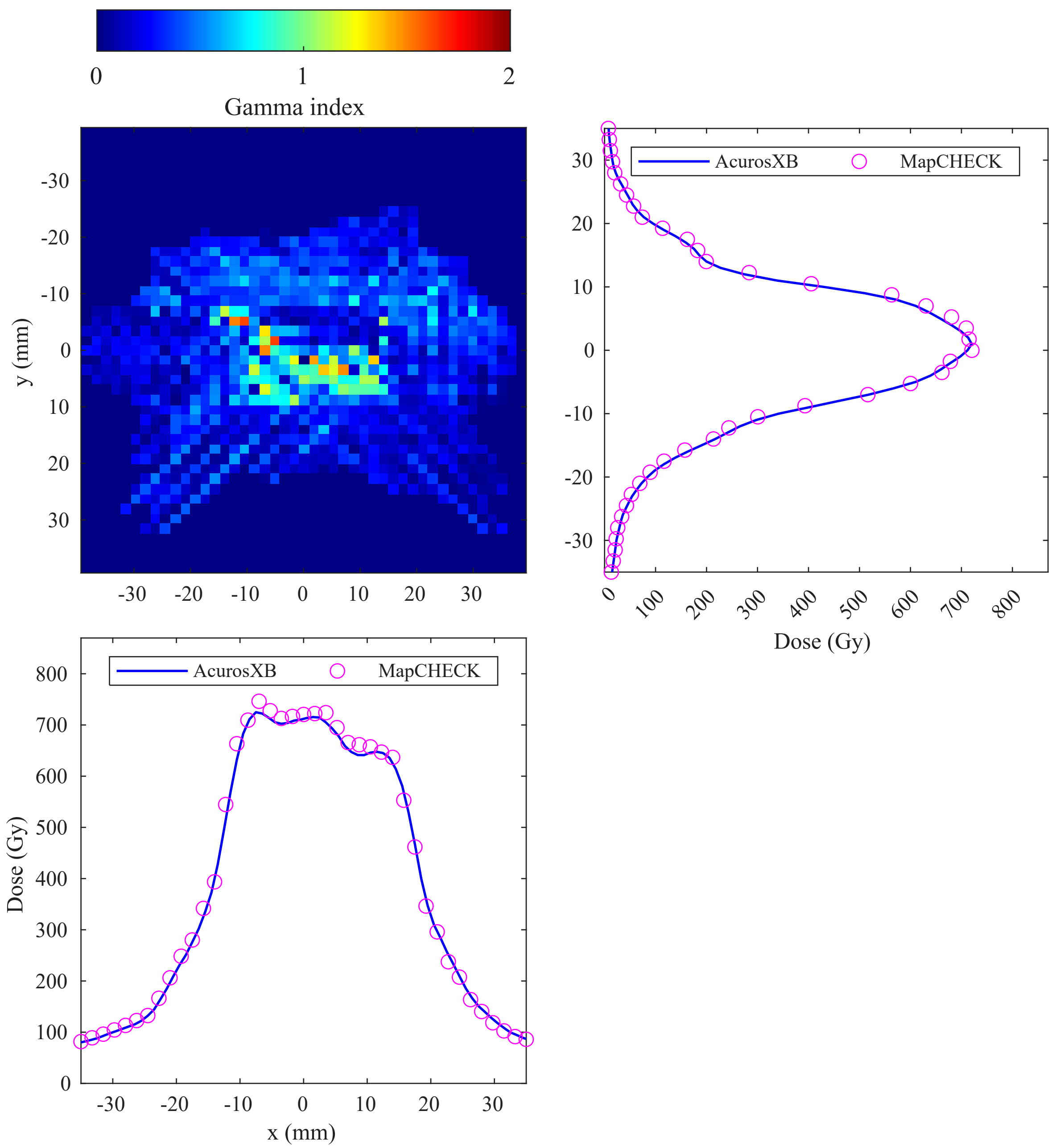

Figure S53. Measurement 51. Plan 34, 2 targets, equivalent diameter 3.6 mm. SRS MapCHECK fraction passing gamma 2%/1 mm = 98.3%

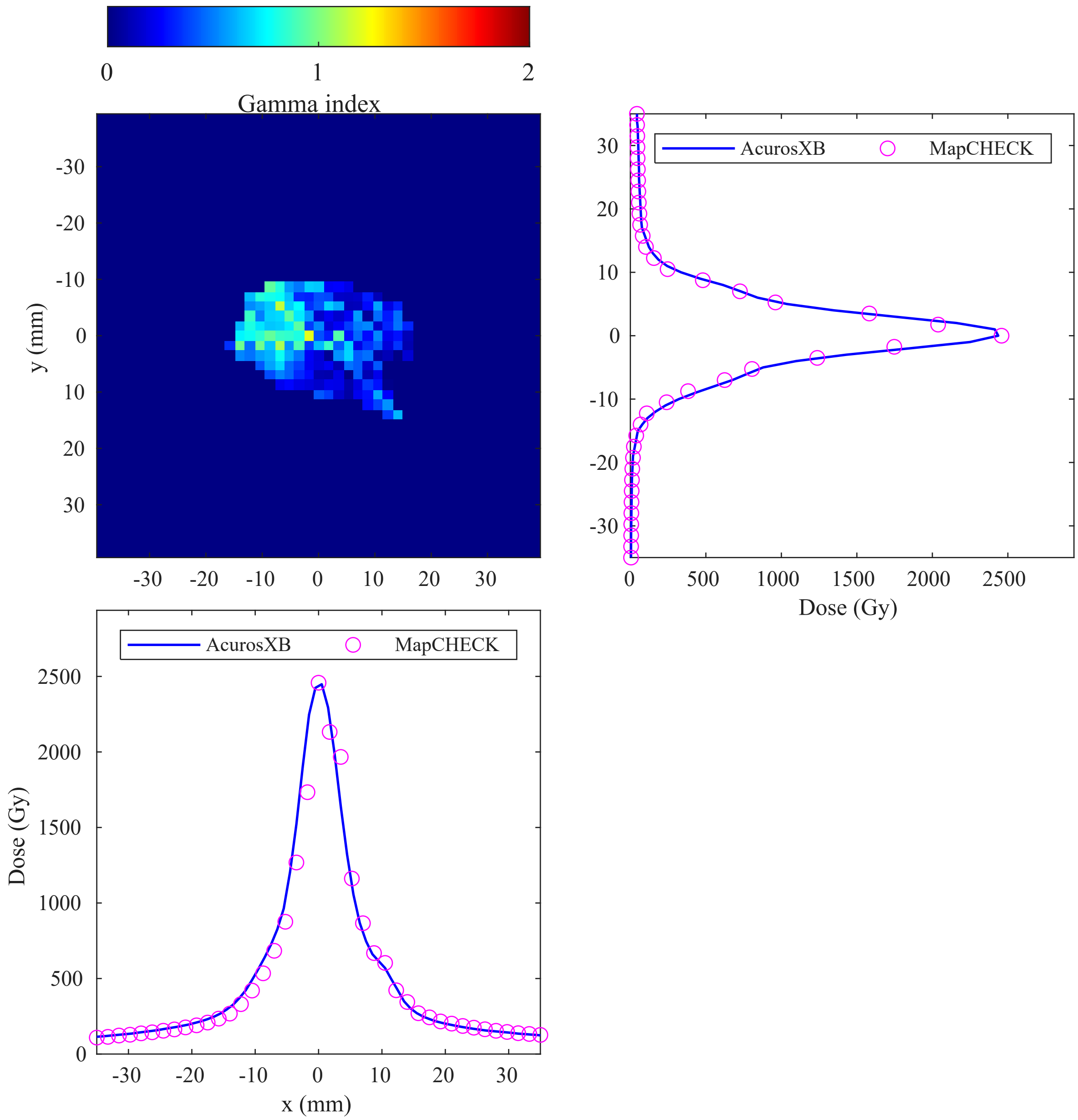

Figure S54. Measurement 52. Plan 34, 2 targets, equivalent diameter 7.0 mm. SRS MapCHECK fraction passing gamma 2%/1 mm = 99.4%

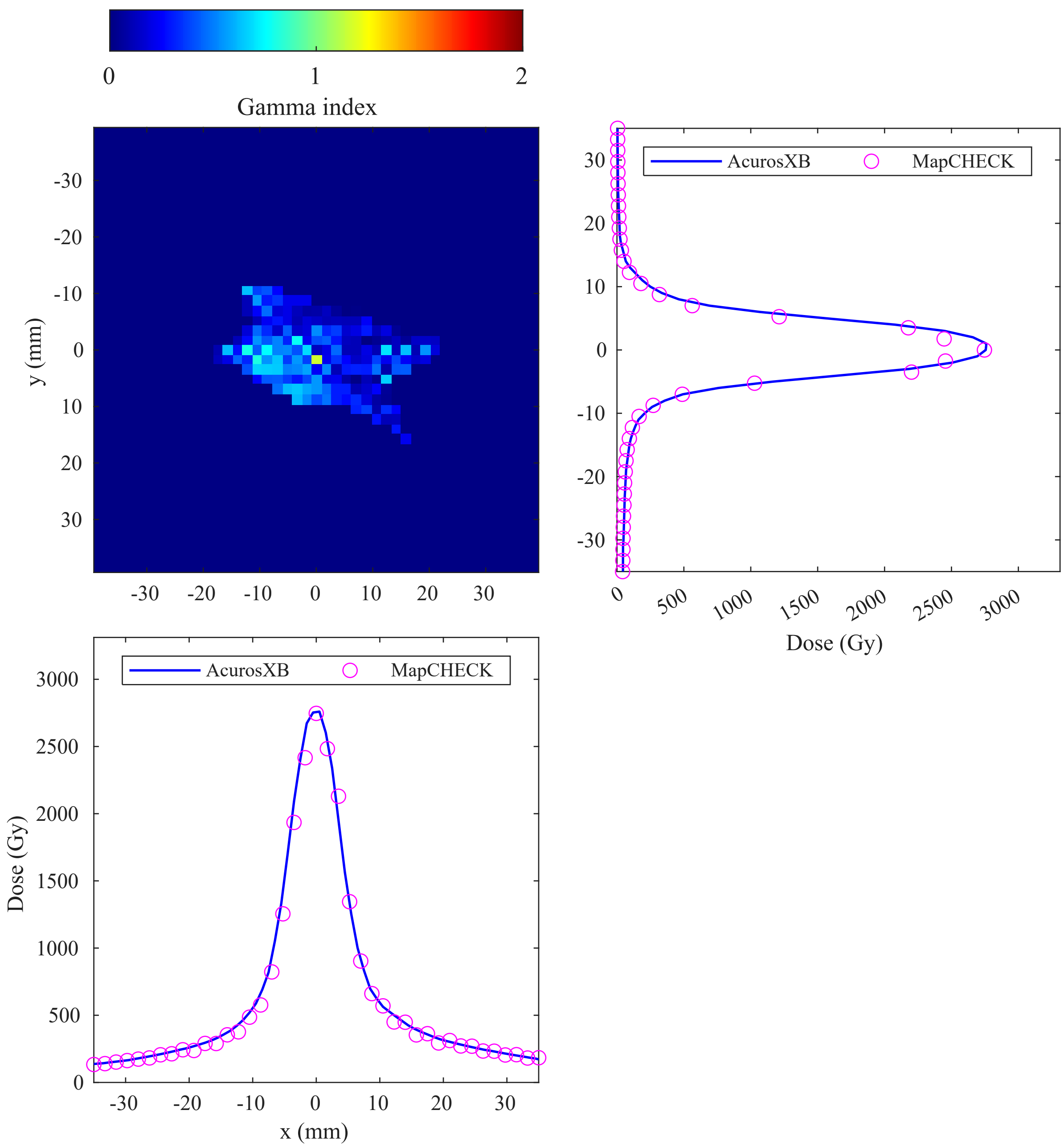

Figure S55. Measurement 53. Plan 35, 7 targets, equivalent diameter 44.7 mm. SRS MapCHECK fraction passing gamma 2%/1 mm = 79.6%

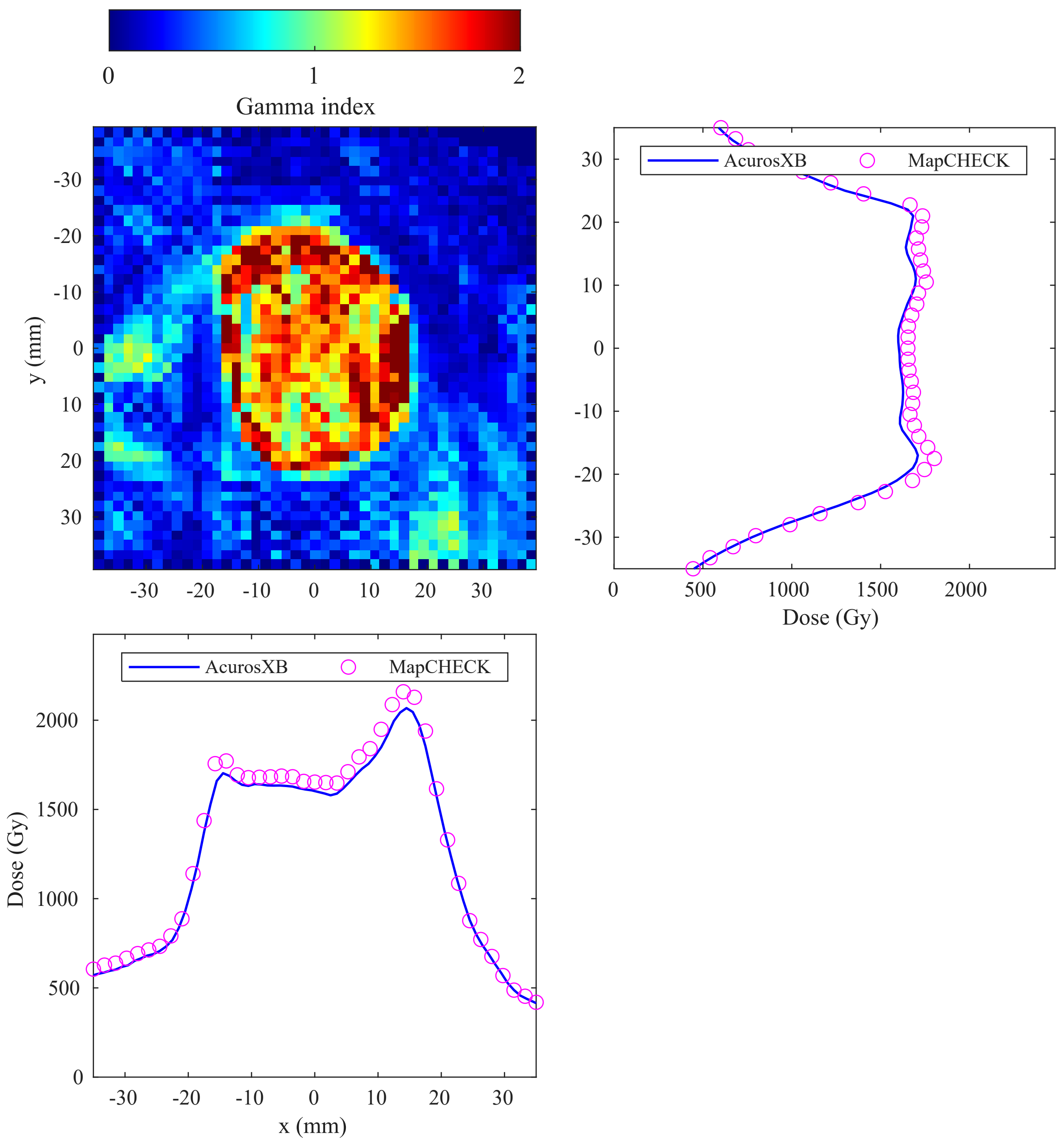

Figure S56. Measurement 54. Plan 35, 7 targets, equivalent diameter 3.5 mm. SRS MapCHECK fraction passing gamma 2%/1 mm = 99.5%

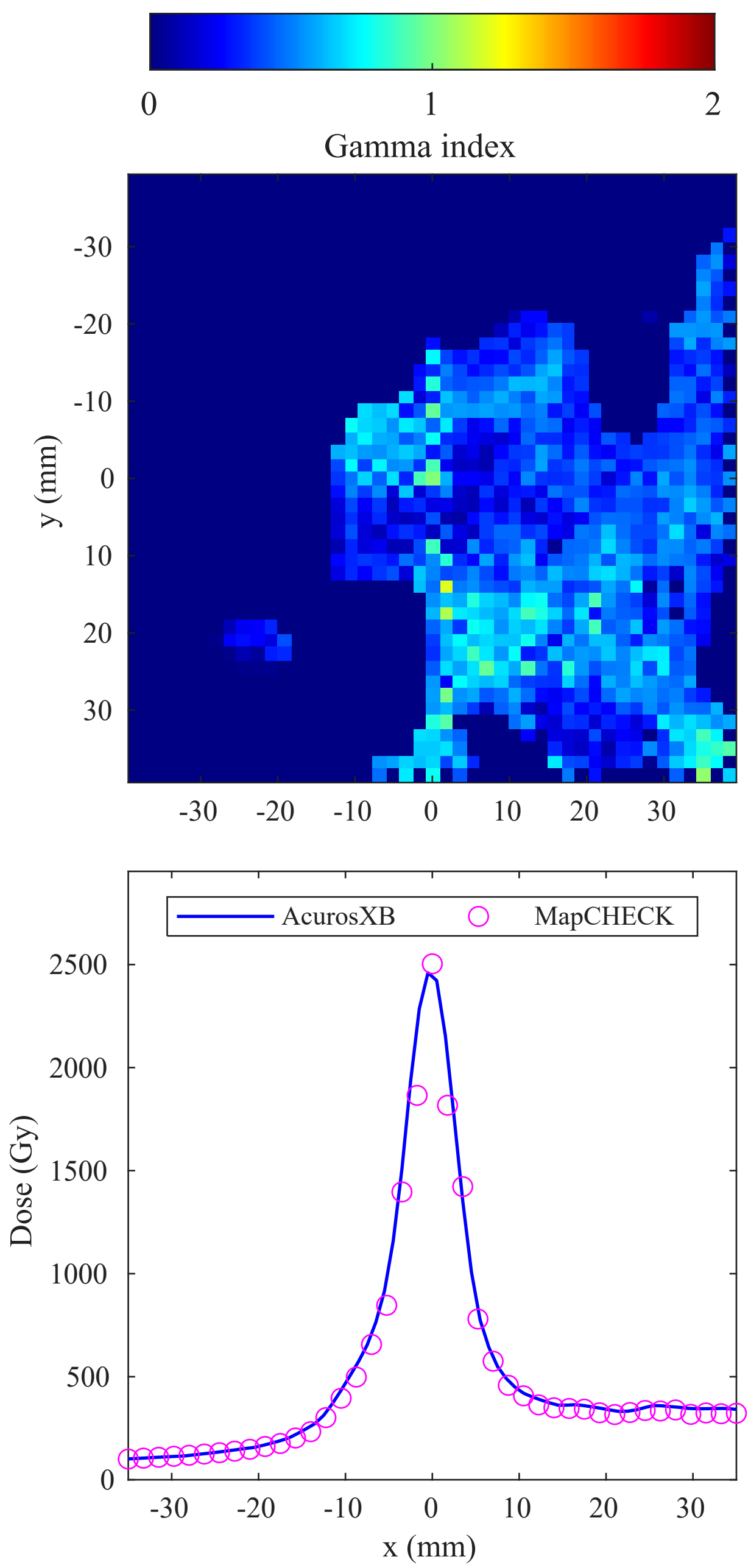

Figure S57. Measurement 55. Plan 36, 2 targets, equivalent diameter 9.2 mm. SRS MapCHECK fraction passing gamma 2%/1 mm = 100.0%

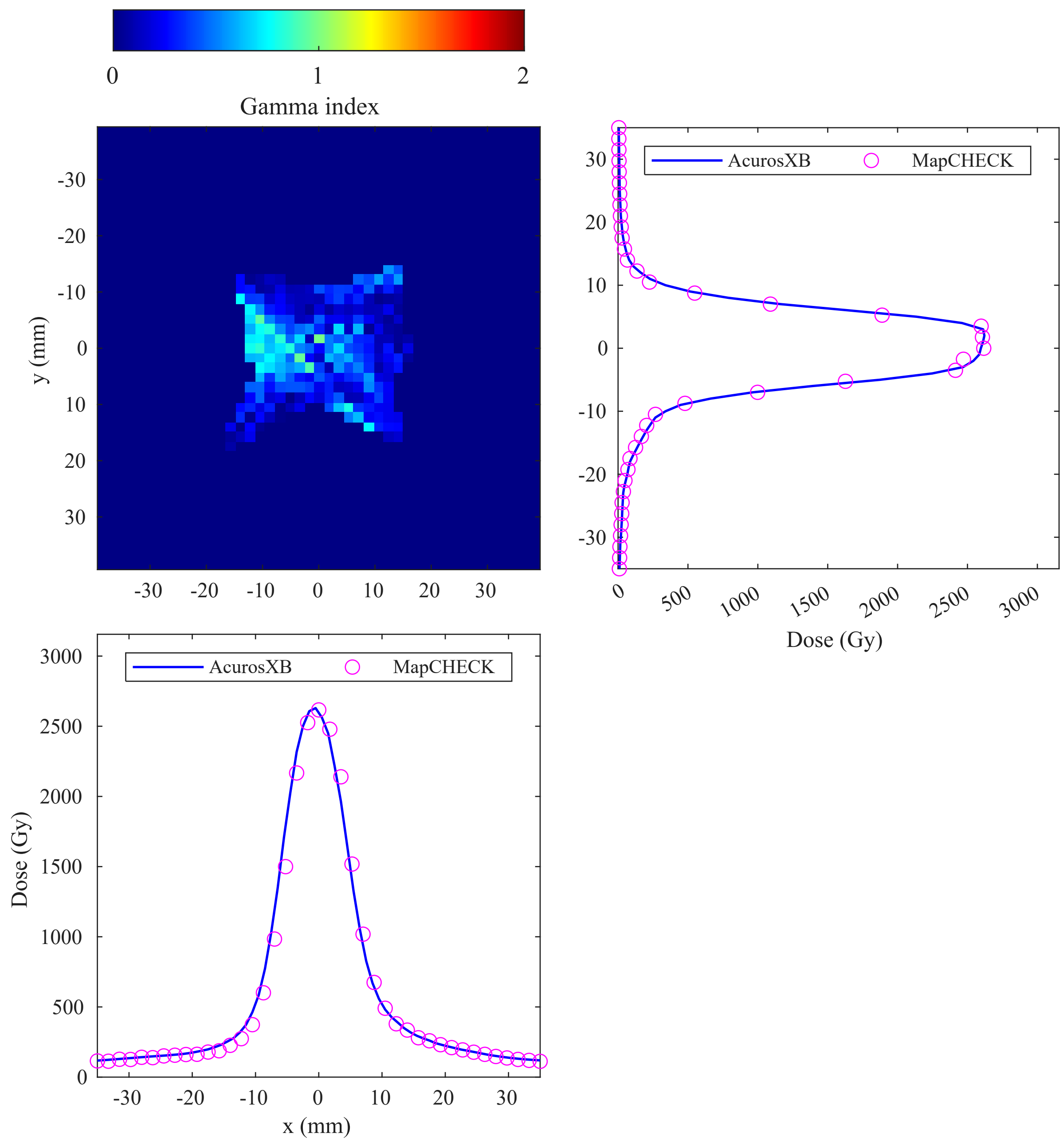

Figure S58. Measurement 56. Plan 36, 2 targets, equivalent diameter 5.6 mm. SRS MapCHECK fraction passing gamma 2%/1 mm = 91.8%

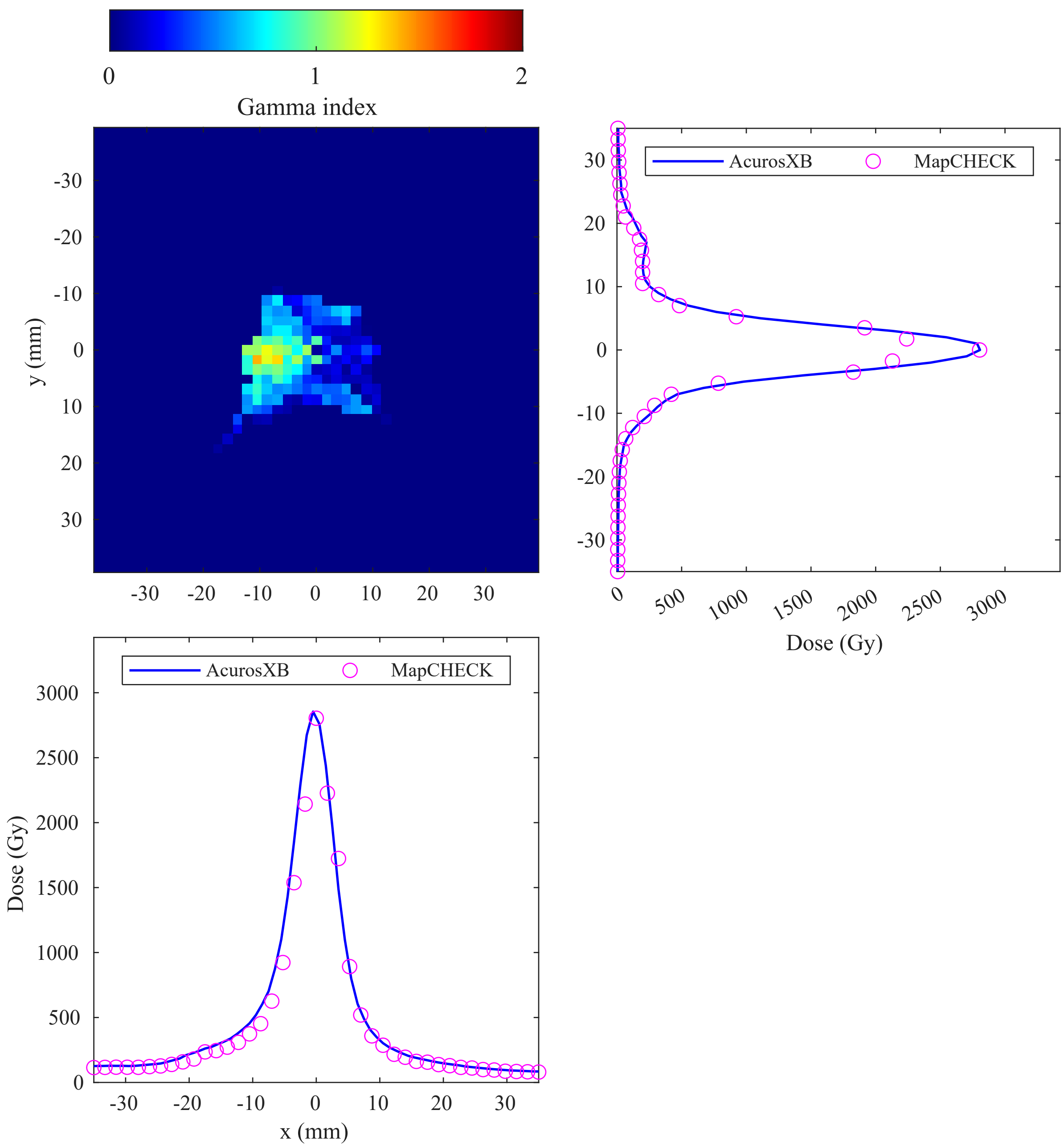

Figure S59. Measurement 57. Plan 37, single target, equivalent diameter 10.5 mm. SRS MapCHECK fraction passing gamma 2%/1 mm = 98.0%

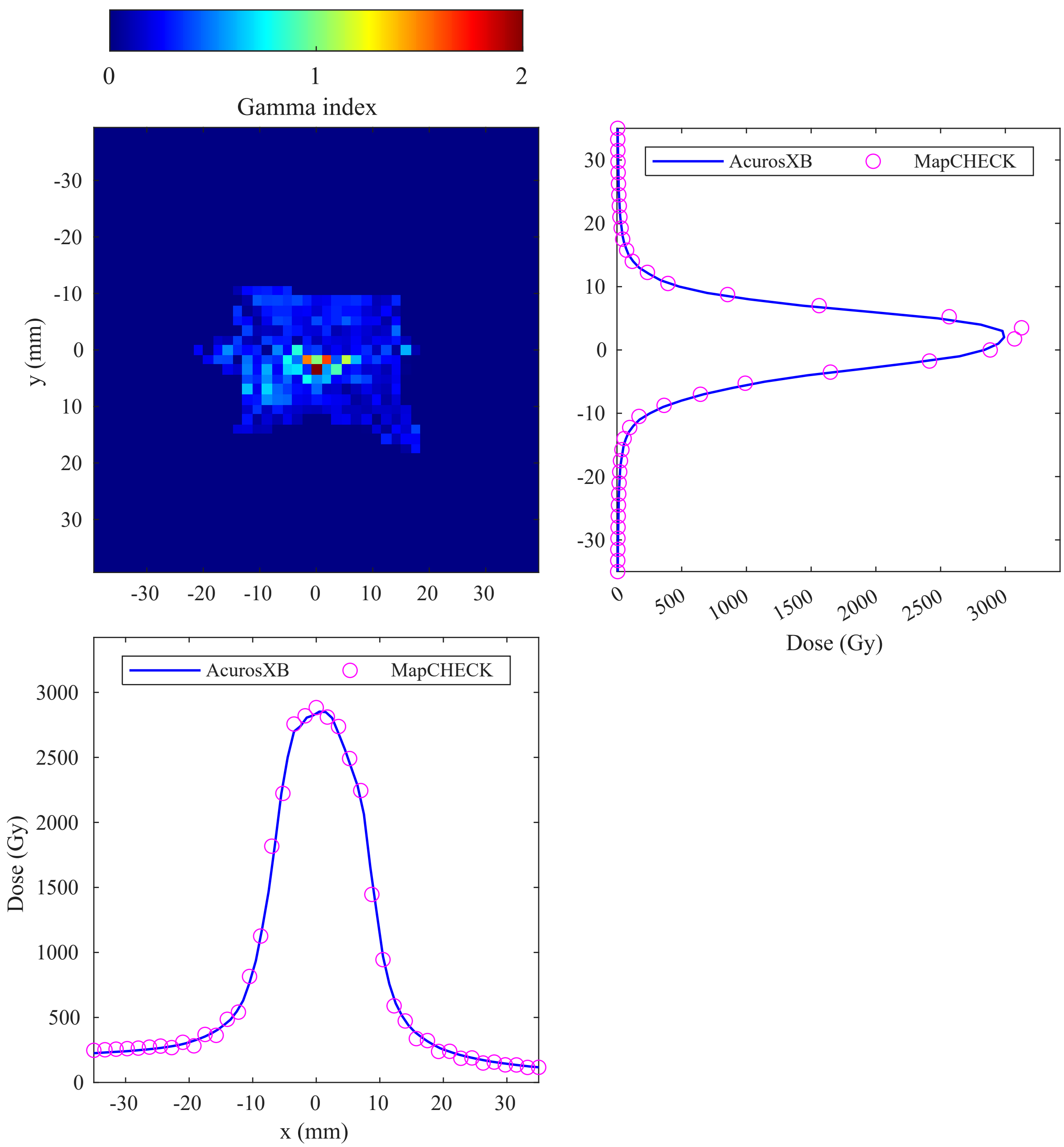

Figure S60. Measurement 58. Plan 38, 3 targets, equivalent diameter 5.8 mm. SRS MapCHECK fraction passing gamma 2%/1 mm = 99.6%

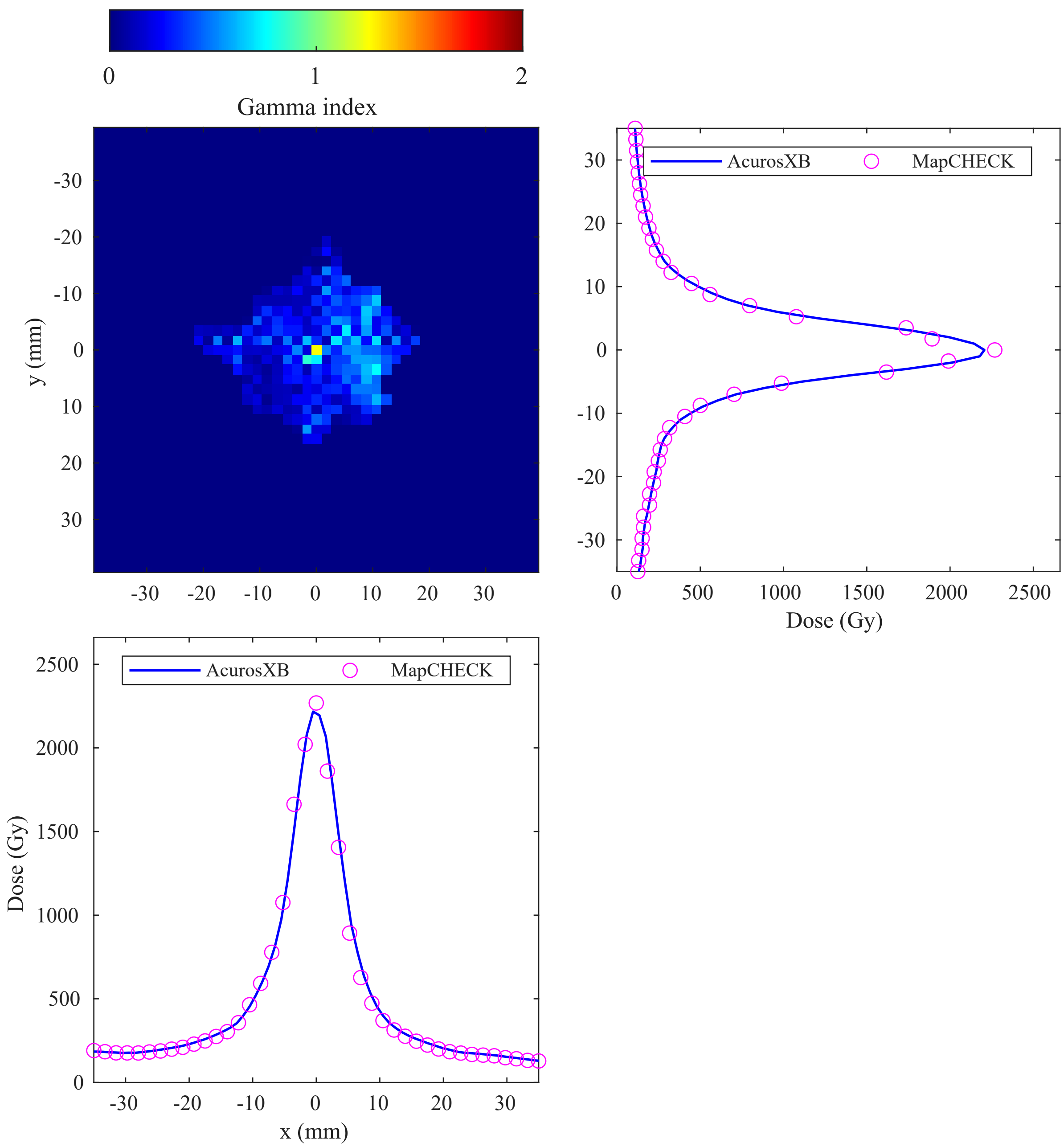

Figure S61. Measurement 59. Plan 38, 3 targets, equivalent diameter 19.9 mm. SRS MapCHECK fraction passing gamma 2%/1 mm = 99.7%

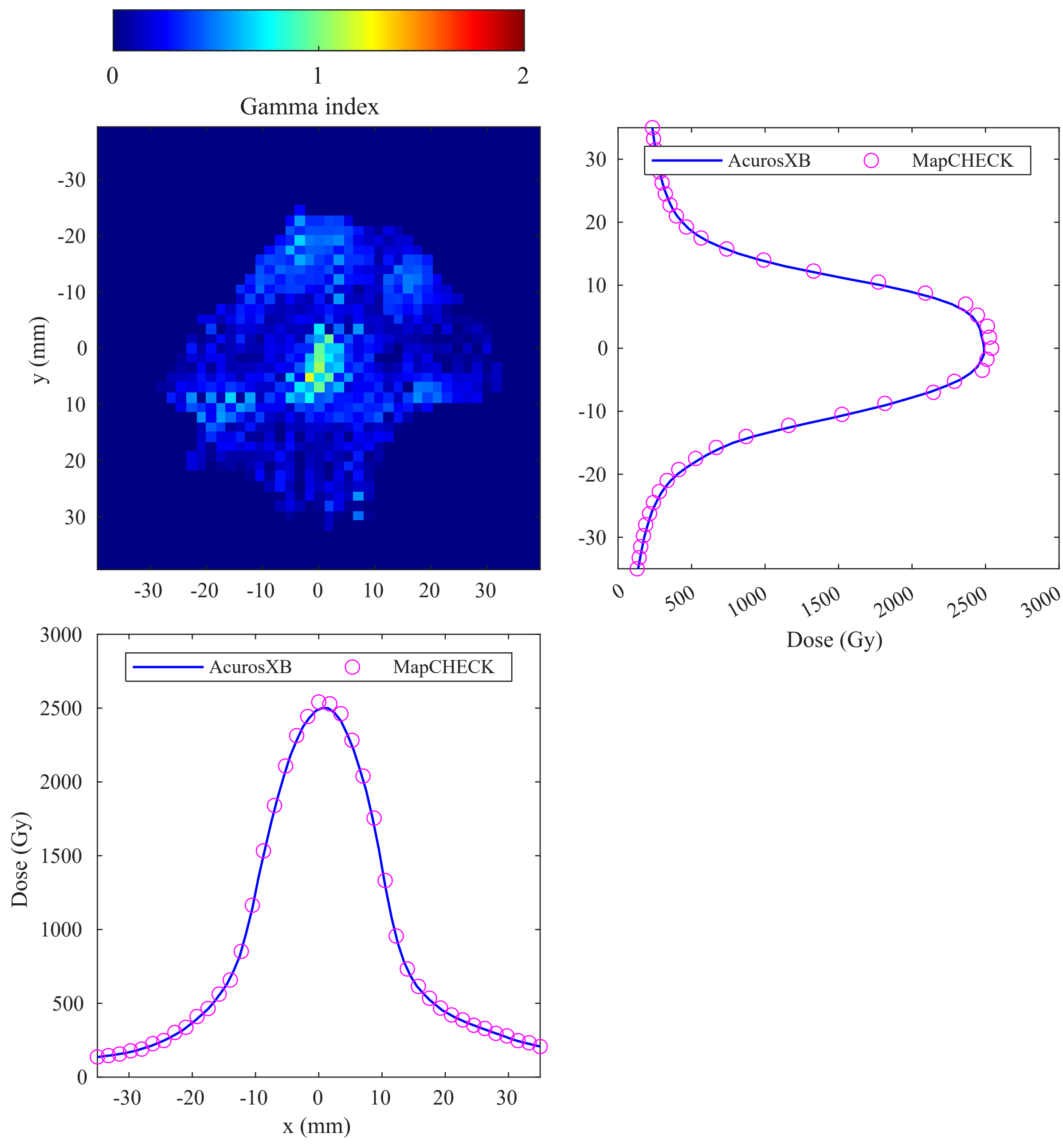

Figure S62. Measurement 60. Plan 39, 4 targets, equivalent diameter 5.7 mm. SRS MapCHECK fraction passing gamma 2%/1 mm = 99.6%

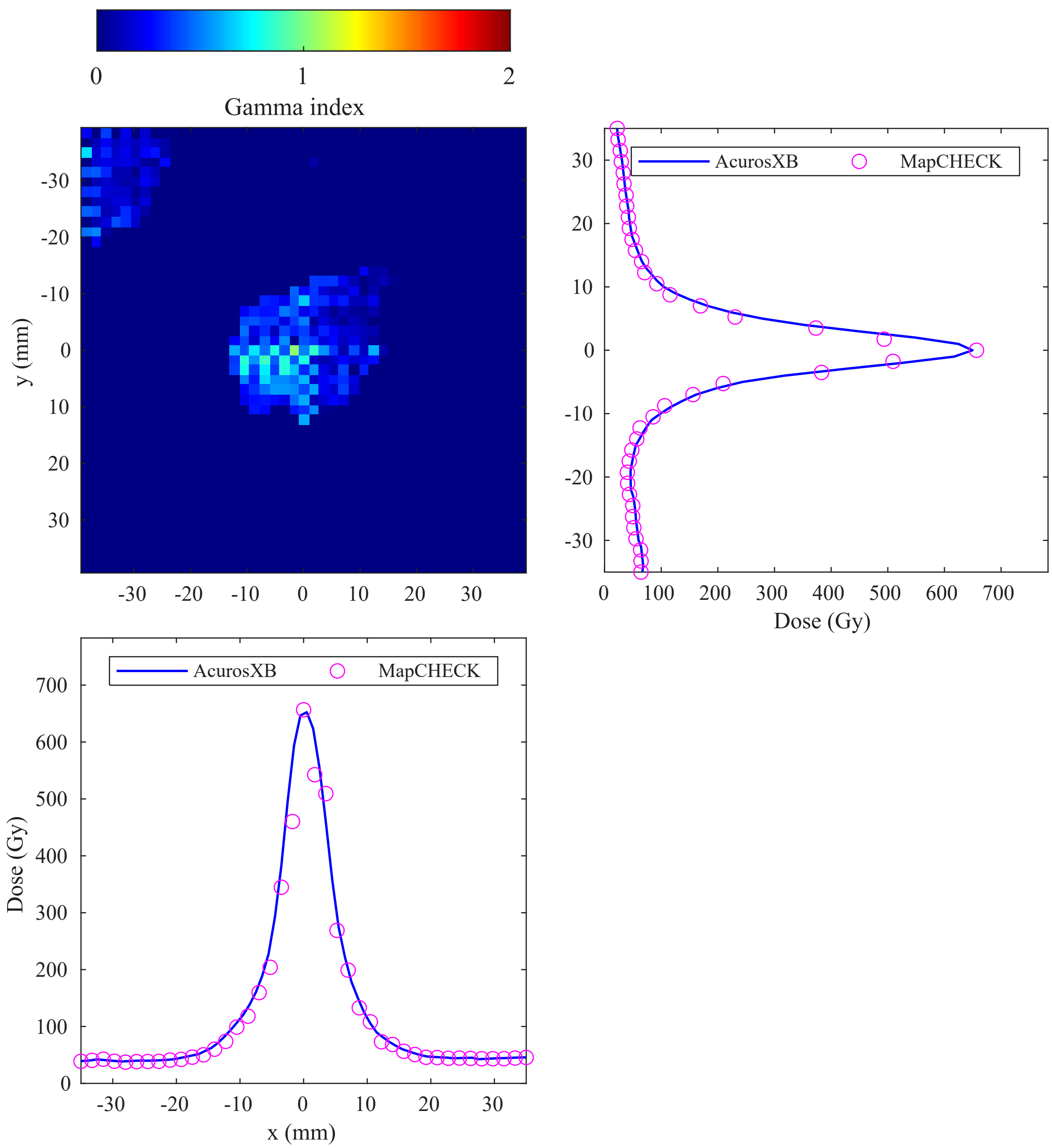

Figure S63. Measurement 61. Plan 39, 4 targets, equivalent diameter 14.7 mm. SRS MapCHECK fraction passing gamma 2%/1 mm = 99.5%

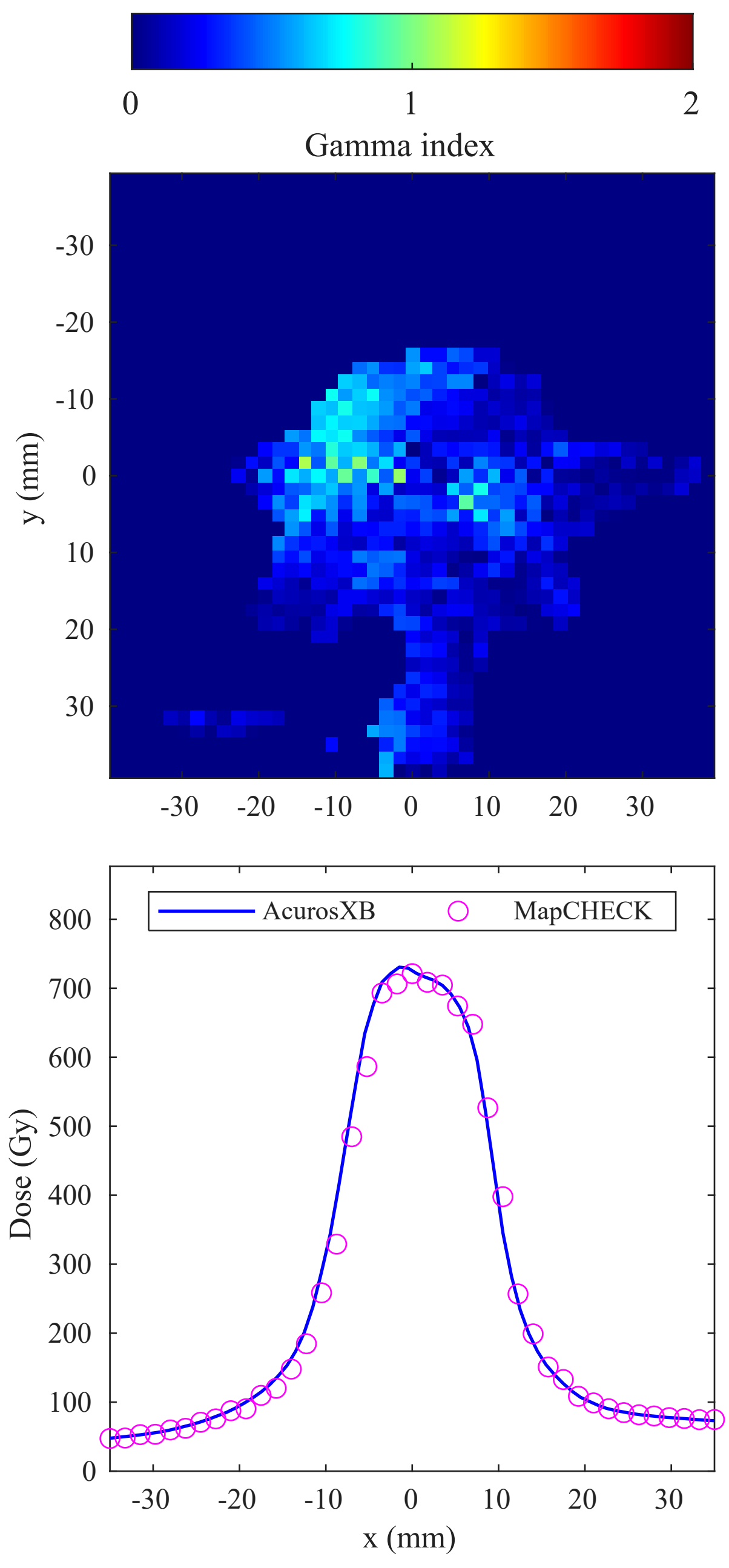

Figure S64. Measurement 62. Plan 40, 17 targets, equivalent diameter 5.8 mm. SRS MapCHECK fraction passing gamma 2%/1 mm = 98.8%

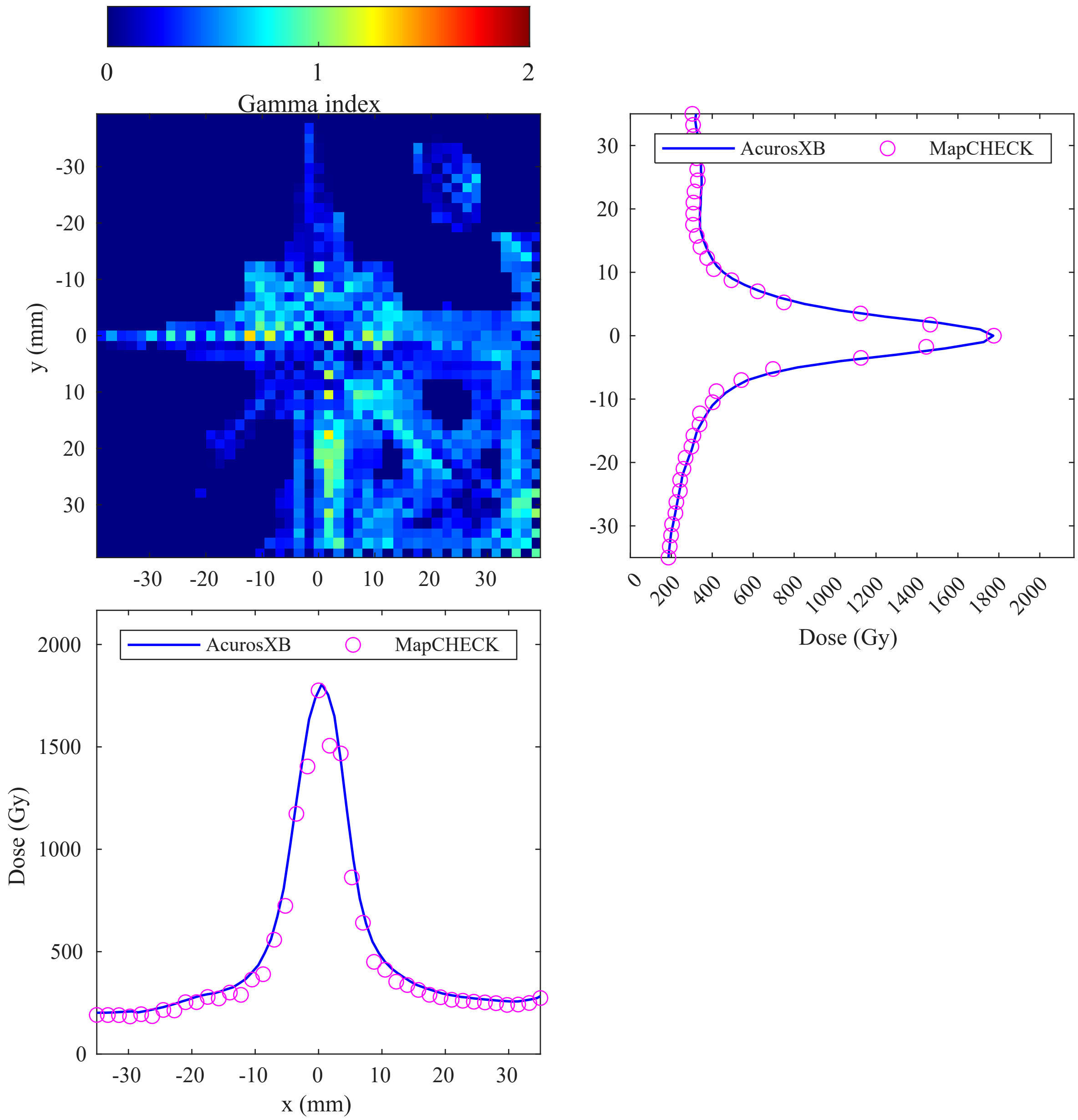

Figure S65. Measurement 63. Plan 40, 17 targets, equivalent diameter 2.8 mm. SRS MapCHECK fraction passing gamma 2%/1 mm = 97.1%

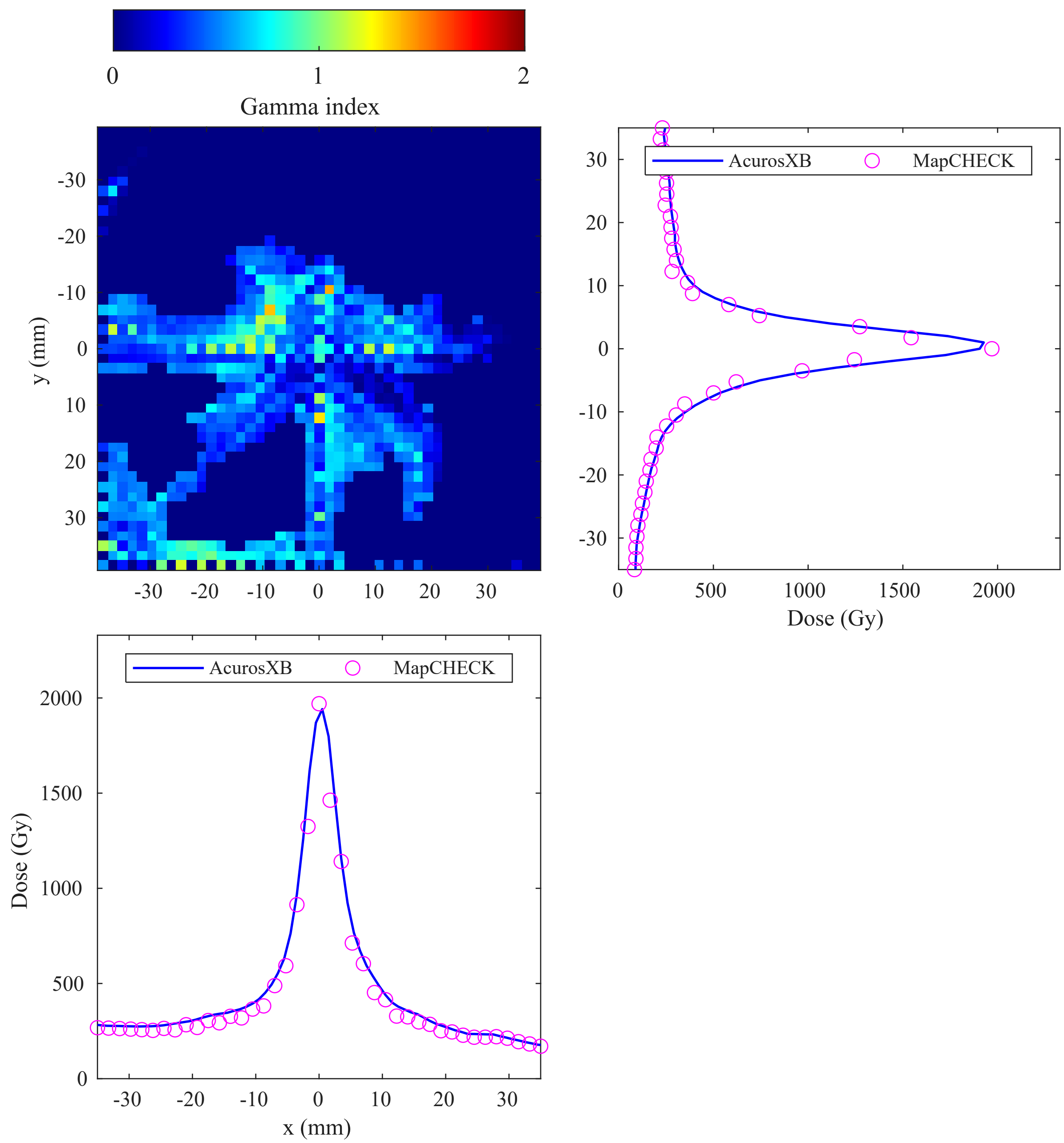

Figure S66. Measurement 64. Plan 41, single target, equivalent diameter 20.1 mm. SRS MapCHECK fraction passing gamma 2%/1 mm = 92.2%

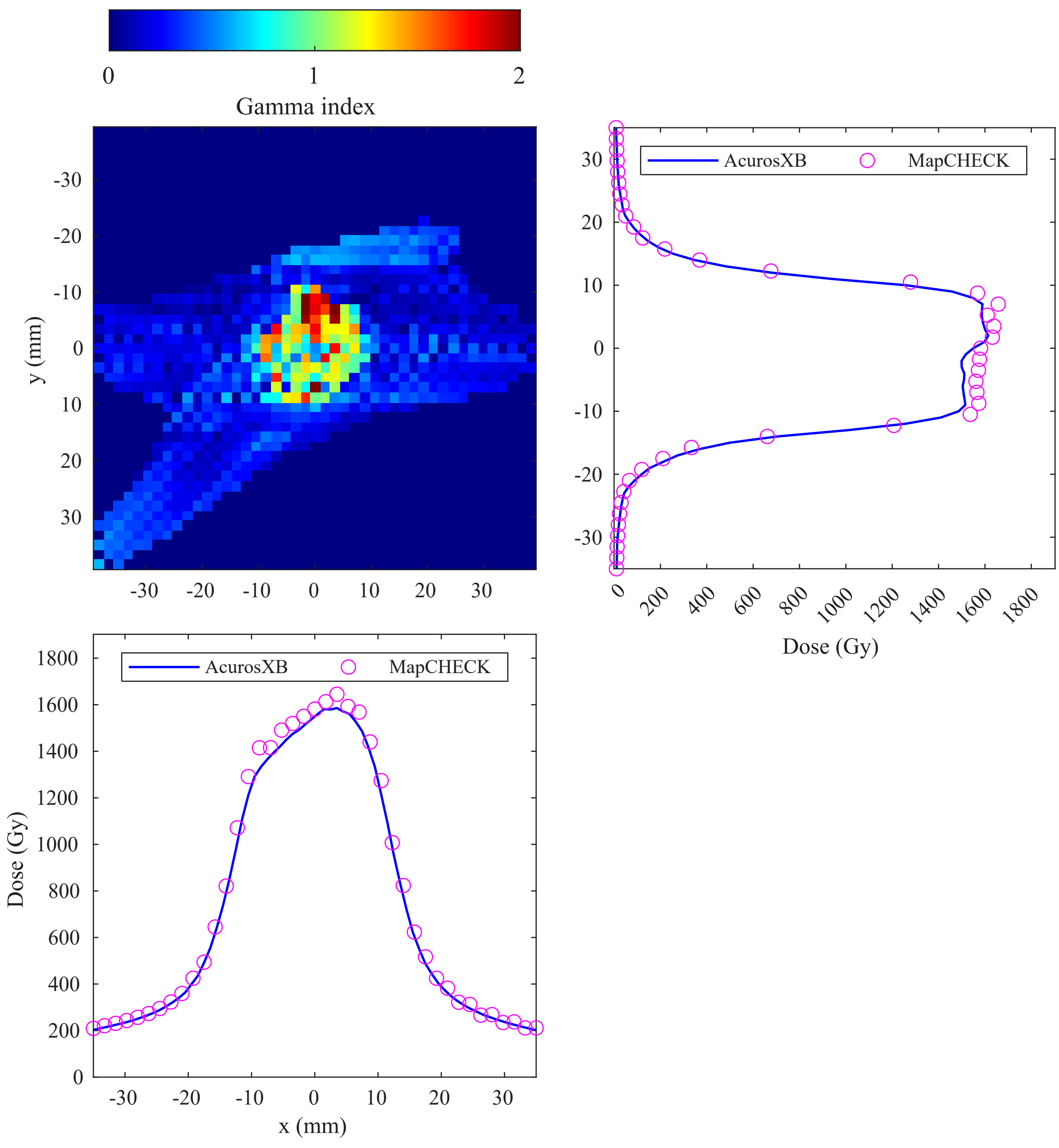

Figure S67. Measurement 65. Plan 42, single target, equivalent diameter 6.0 mm. SRS MapCHECK fraction passing gamma 2%/1 mm = 97.5%

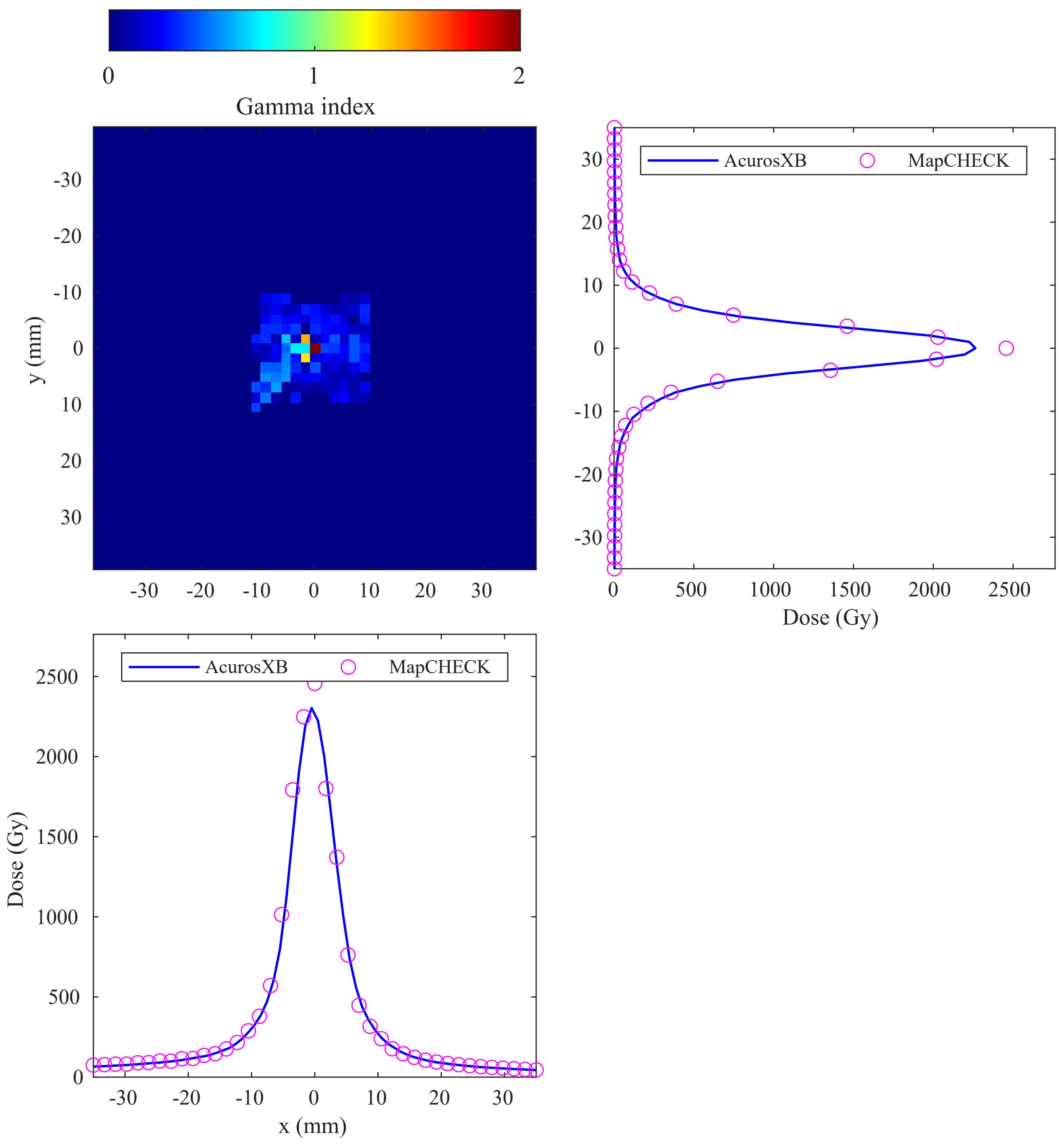

Figure S68. Measurement 66. Plan 43, single target, equivalent diameter 15.9 mm. SRS MapCHECK fraction passing gamma 2%/1 mm = 96.8%

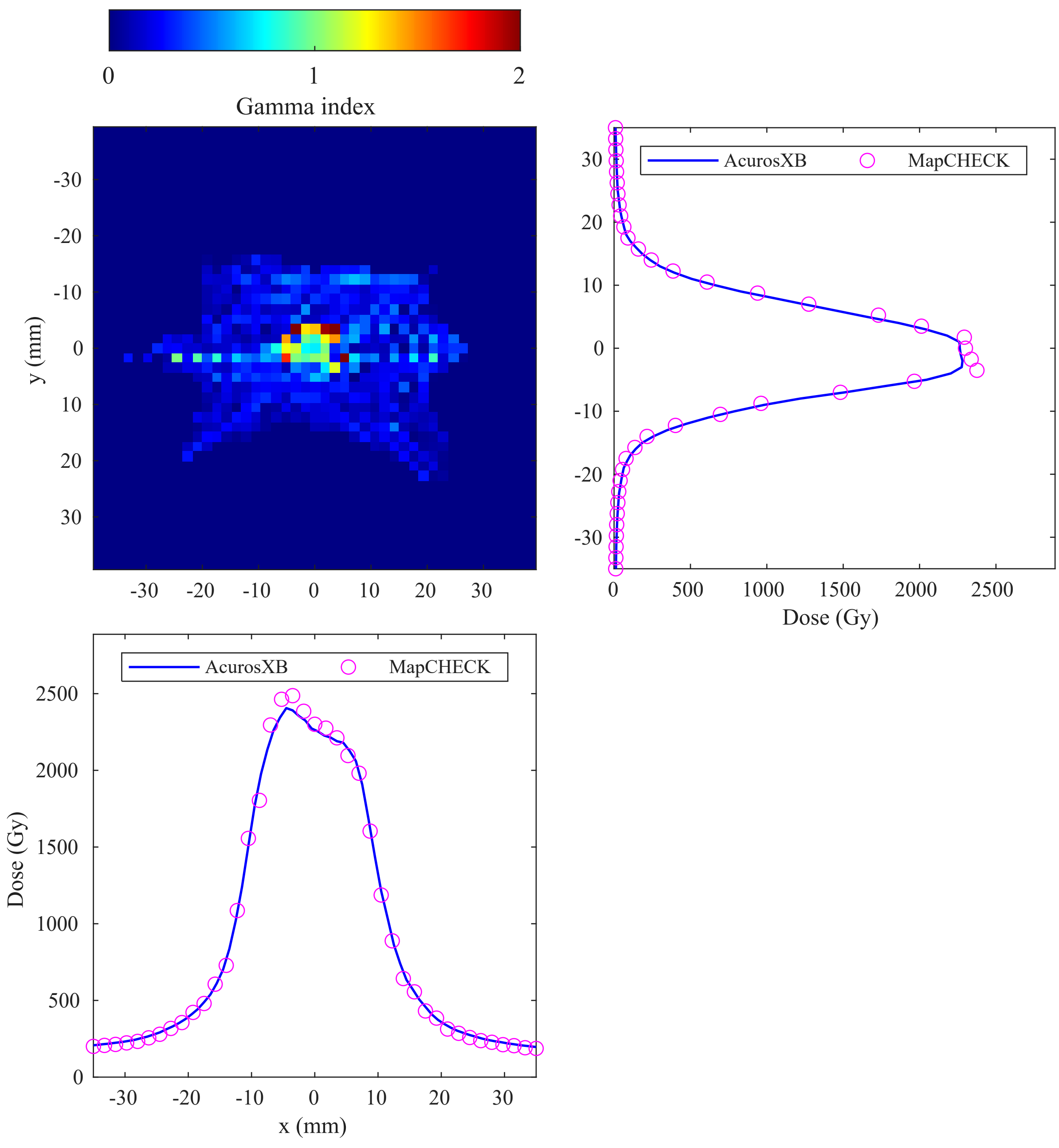

Figure S69. Measurement 67. Plan 44, single target, equivalent diameter 8.6 mm. SRS MapCHECK fraction passing gamma 2%/1 mm = 95.9%

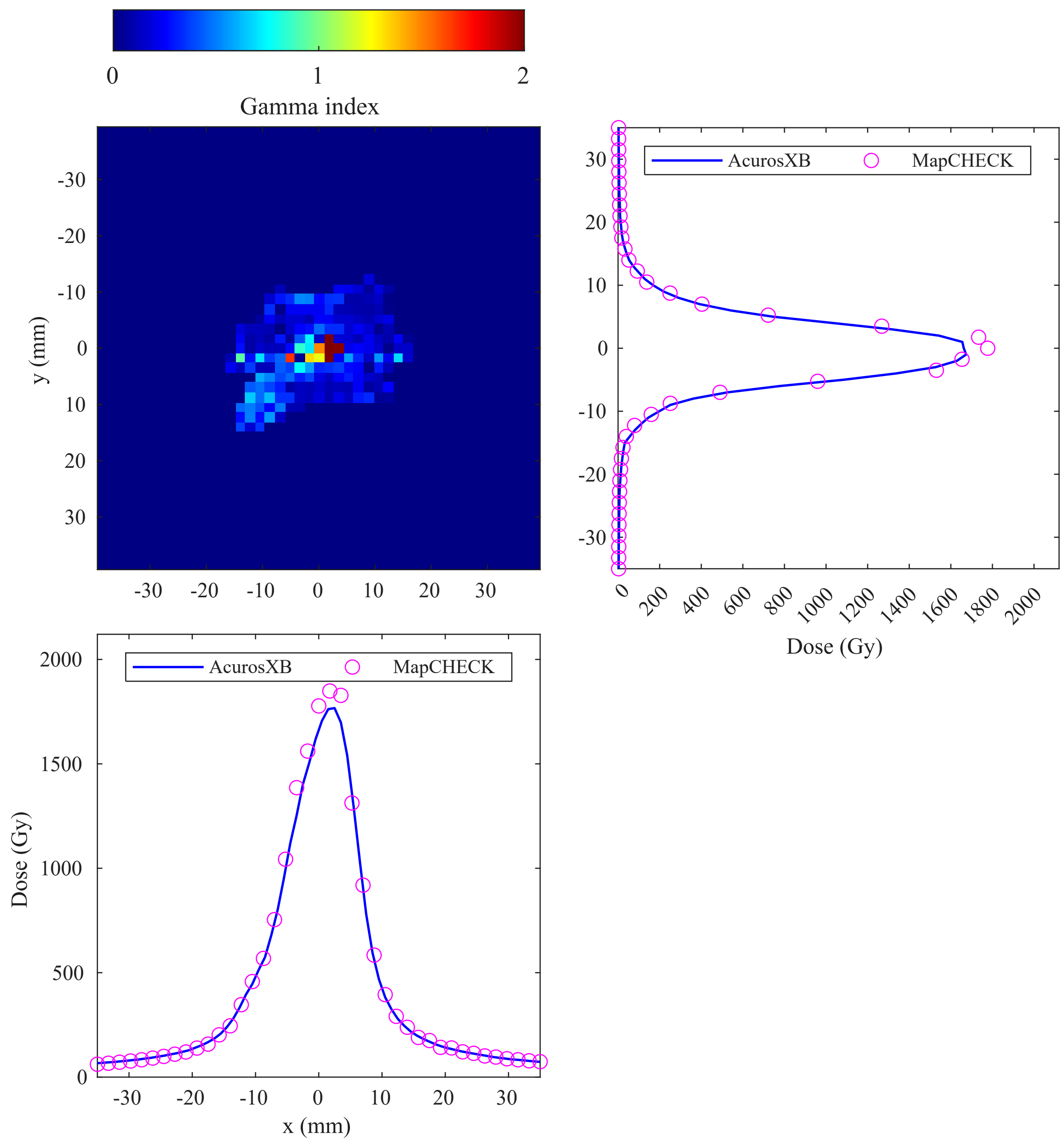

Figure S70. Measurement 68. Plan 45, 2 targets, equivalent diameter 10.8 mm. SRS MapCHECK fraction passing gamma 2%/1 mm = 99.4%

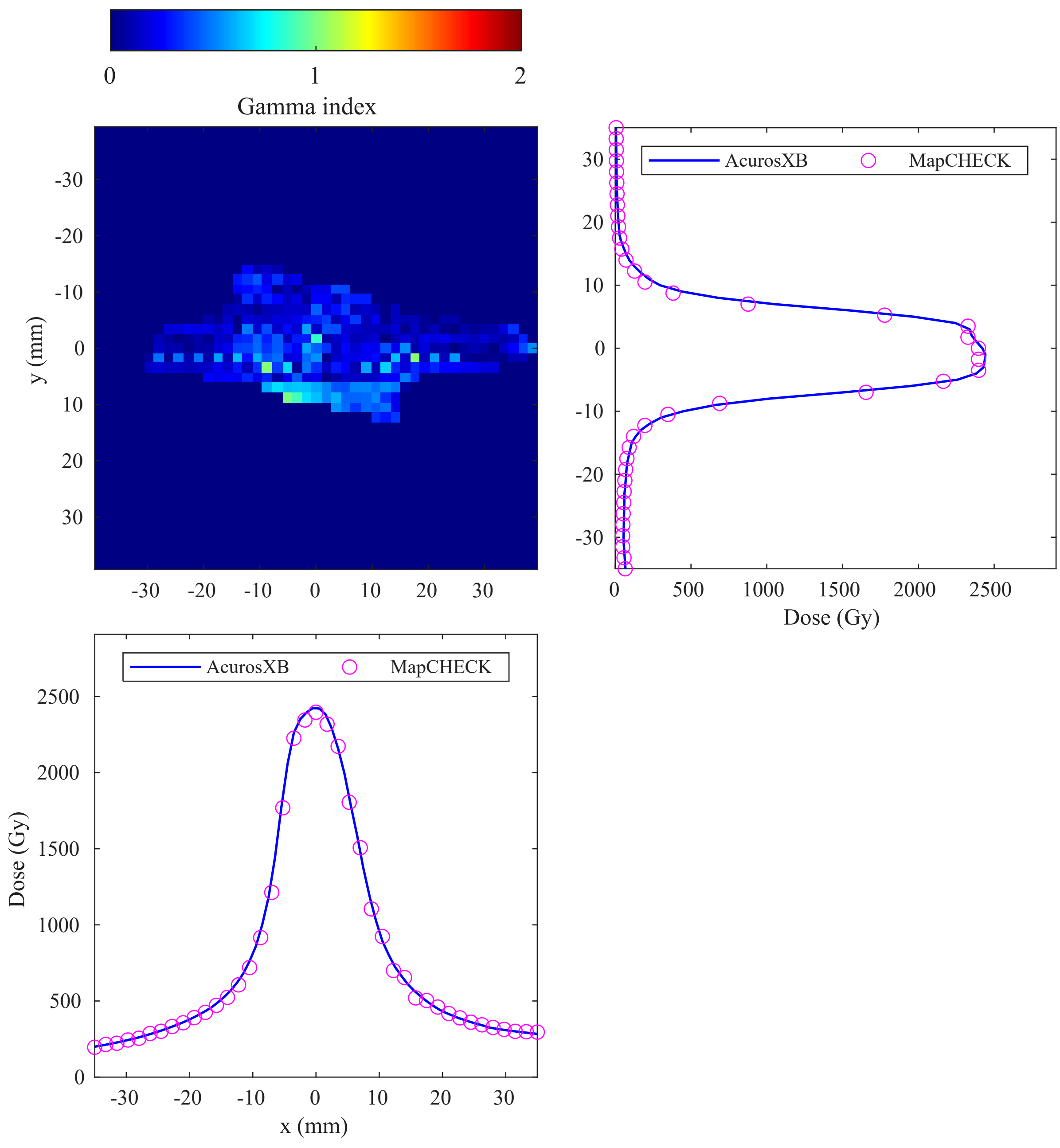

Figure S71. Measurement 69. Plan 45, 2 targets, equivalent diameter 3.4 mm. SRS MapCHECK fraction passing gamma 2%/1 mm = 94.9%

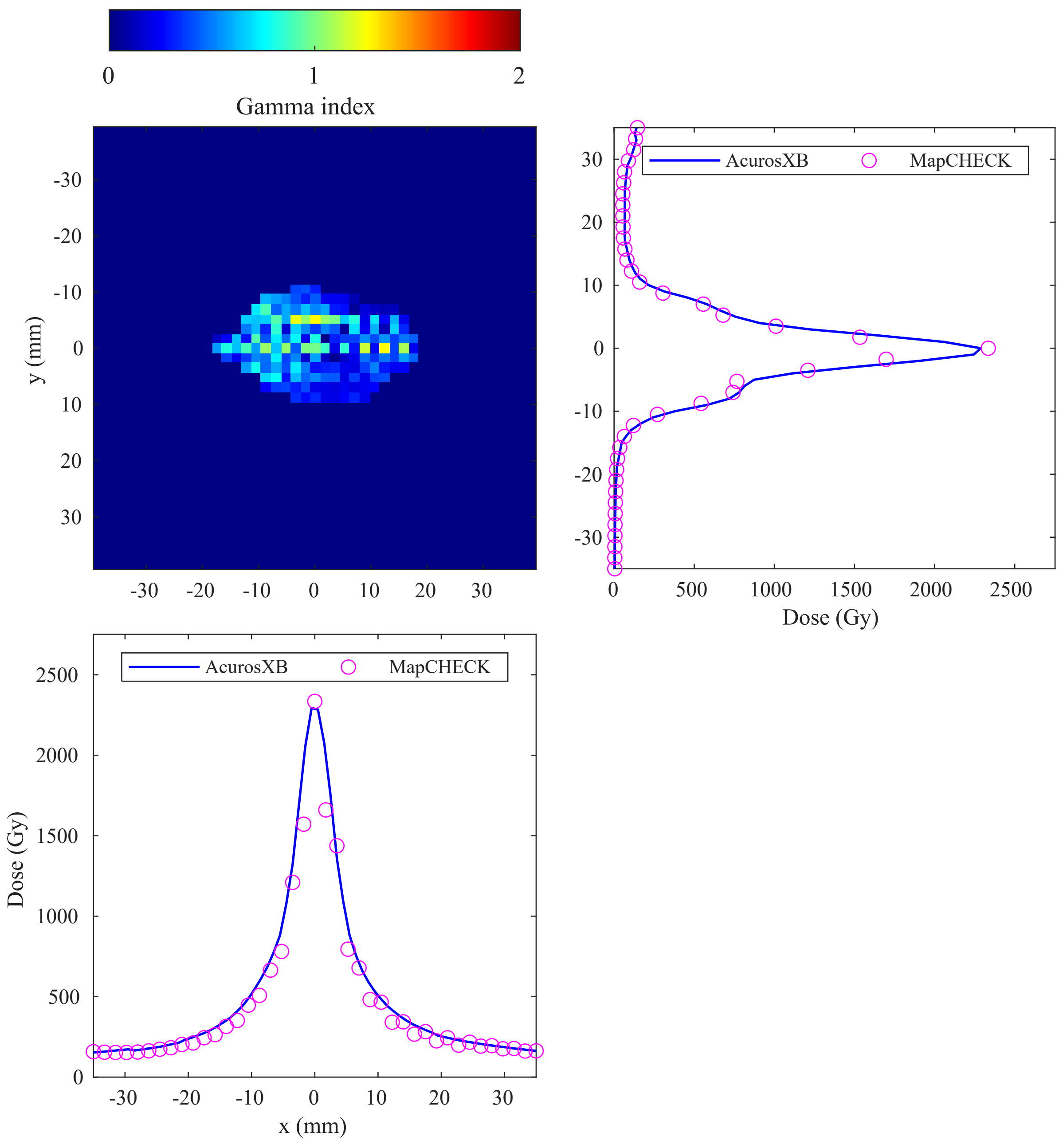

Figure S72. Measurement 70. Plan 46, single target, equivalent diameter 5.9 mm. SRS MapCHECK fraction passing gamma 2%/1 mm = 99.0%

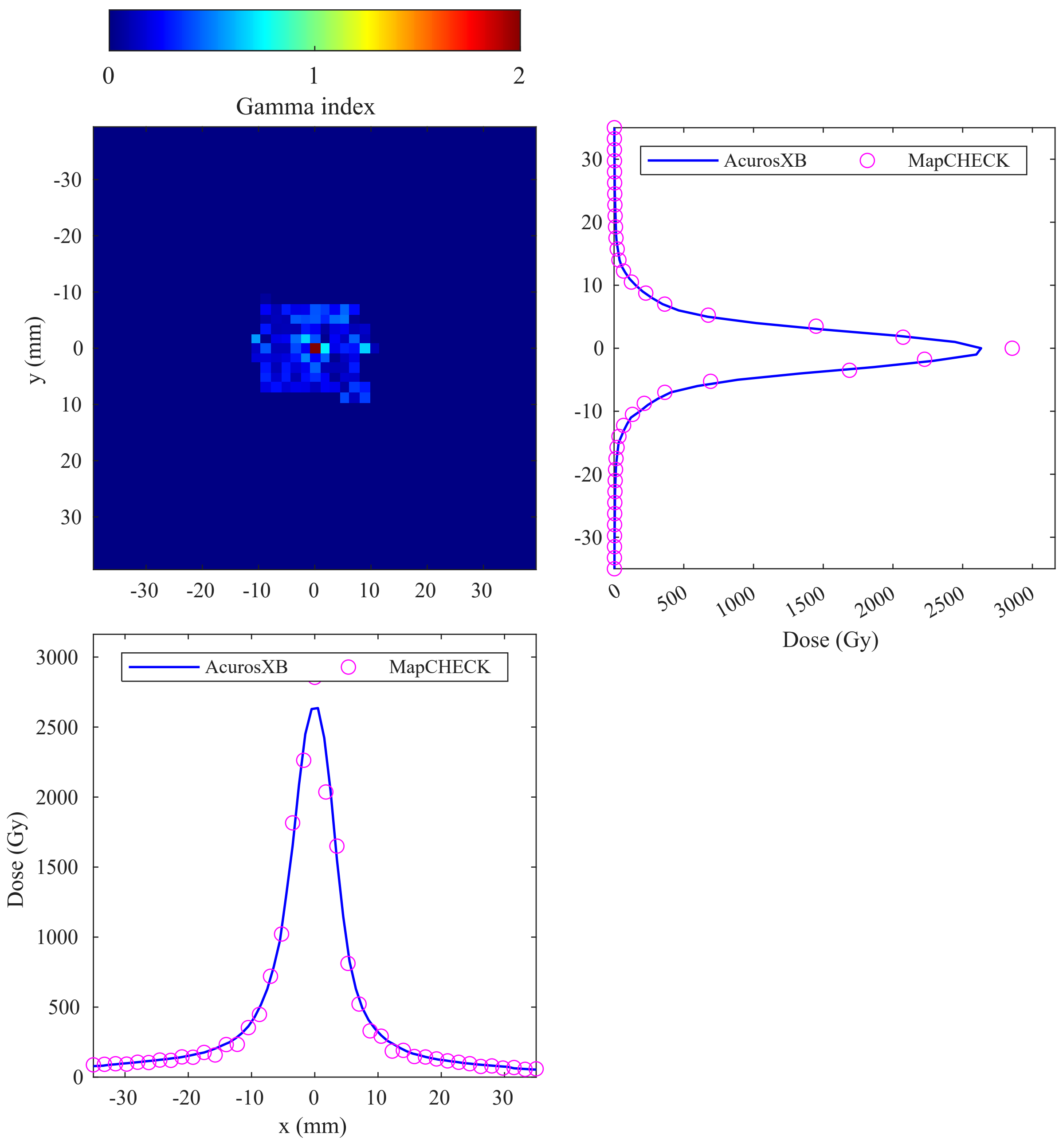

Figure S73. Measurement 71. Plan 47, 2 targets, equivalent diameter 15.7 mm. SRS MapCHECK fraction passing gamma 2%/1 mm = 98.7%

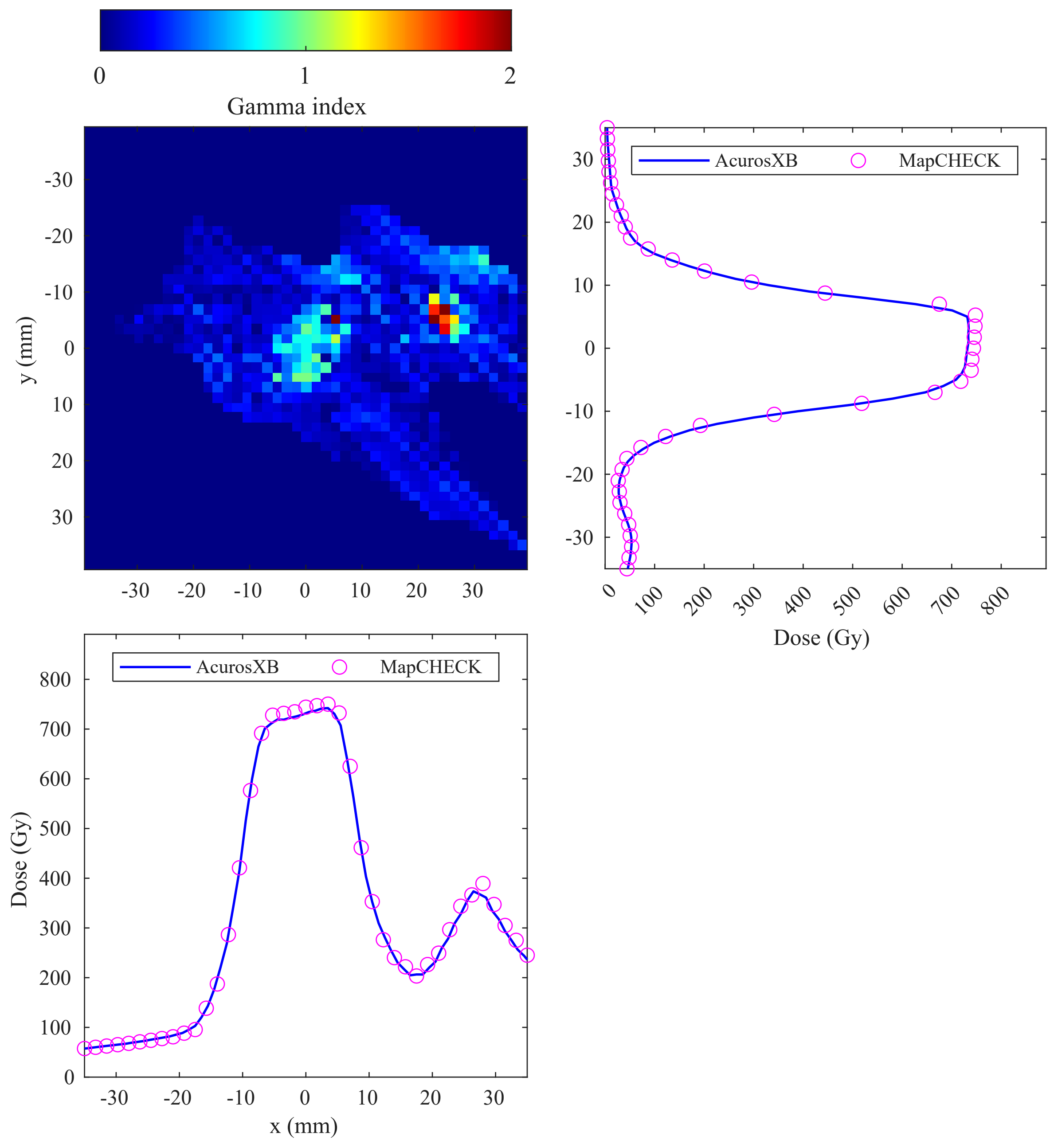

Figure S74. Measurement 72. Plan 47, 2 targets, equivalent diameter 10.8 mm. SRS MapCHECK fraction passing gamma 2%/1 mm = 96.5%

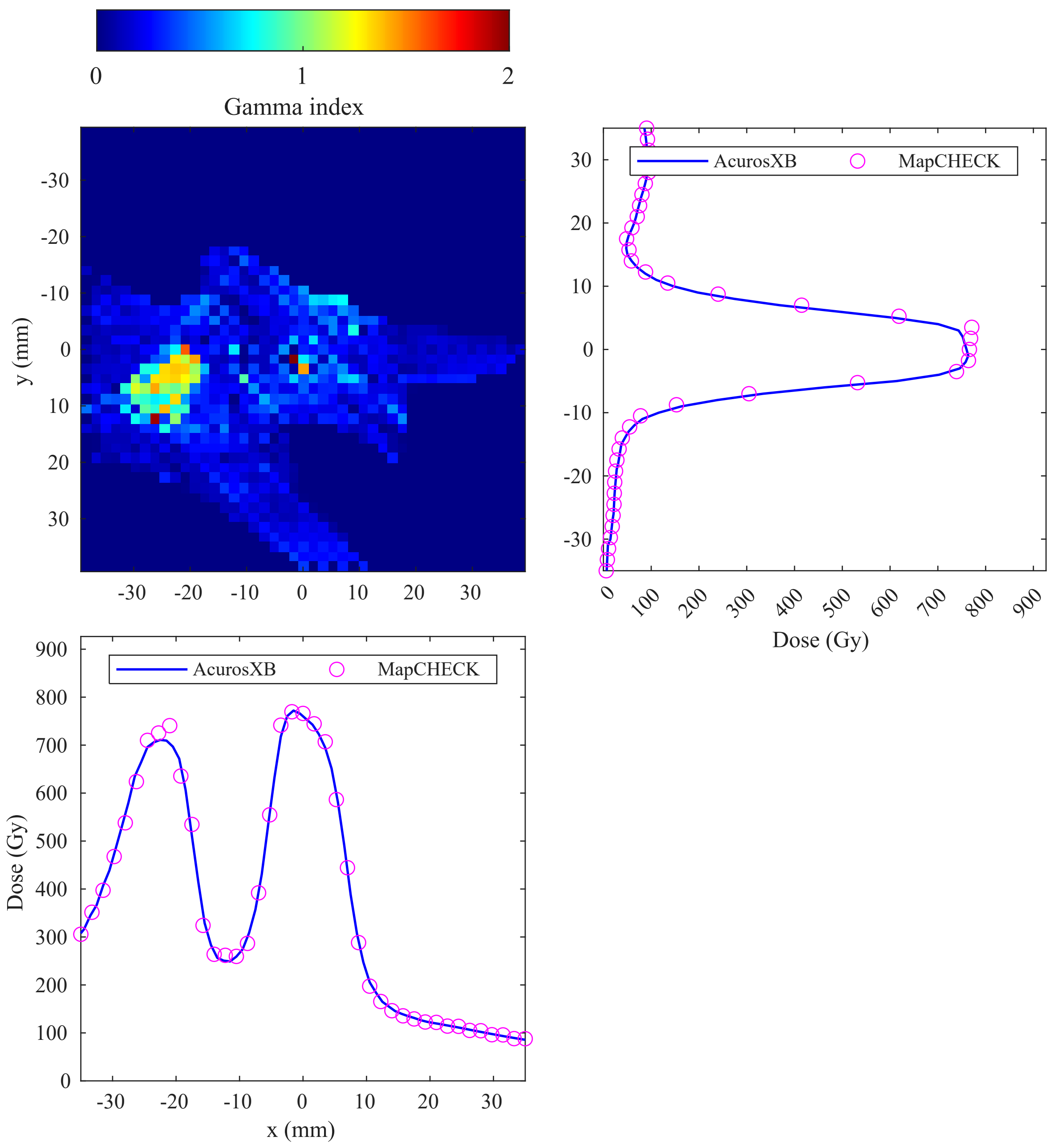

Figure S75. Measurement 73. Plan 48, single target, equivalent diameter 15.2 mm. SRS MapCHECK fraction passing gamma 2%/1 mm = 97.5%

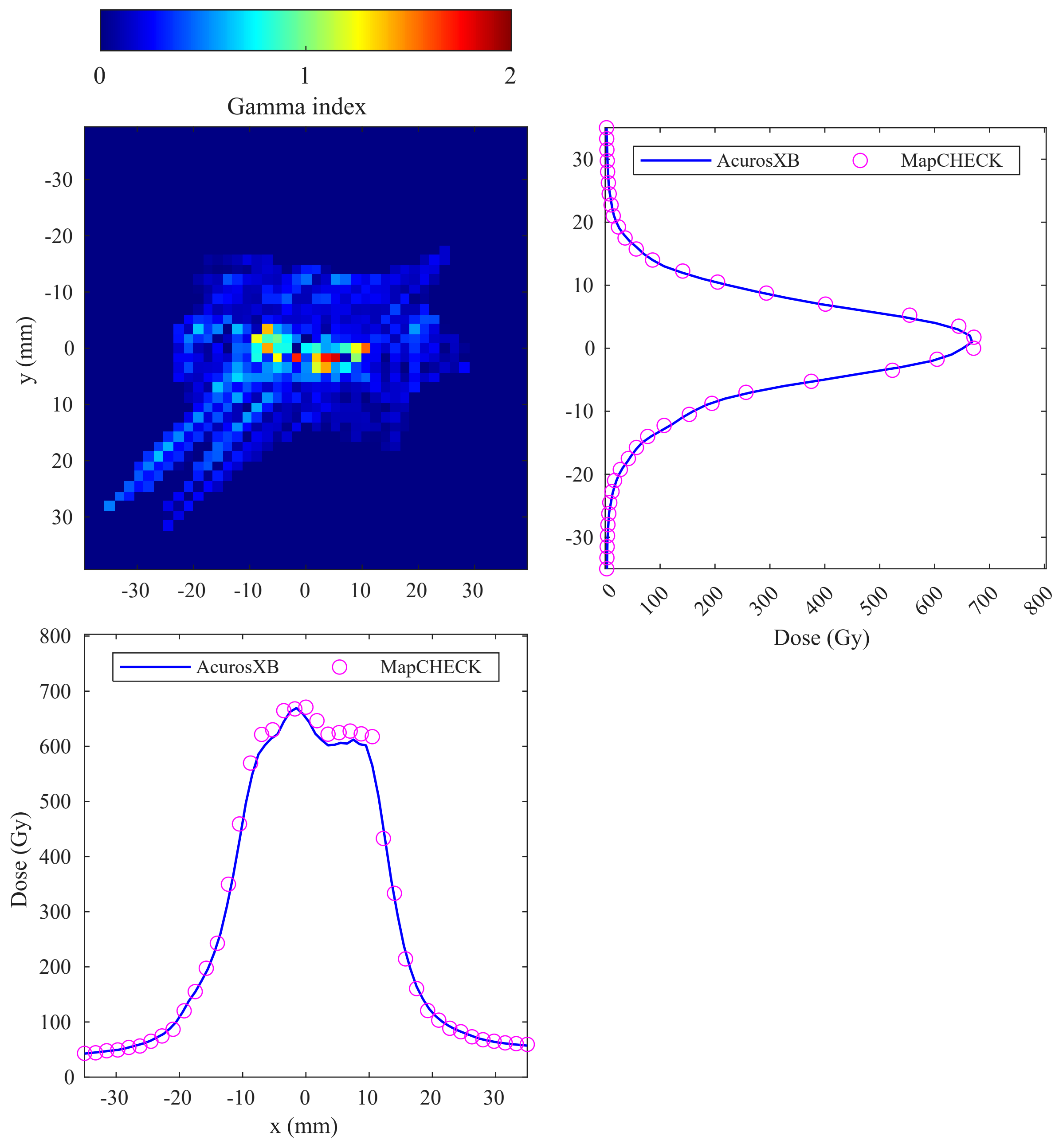

Figure S76. Measurement 74. Plan 49, single target, equivalent diameter 19.0 mm. SRS MapCHECK fraction passing gamma 2%/1 mm = 94.2%

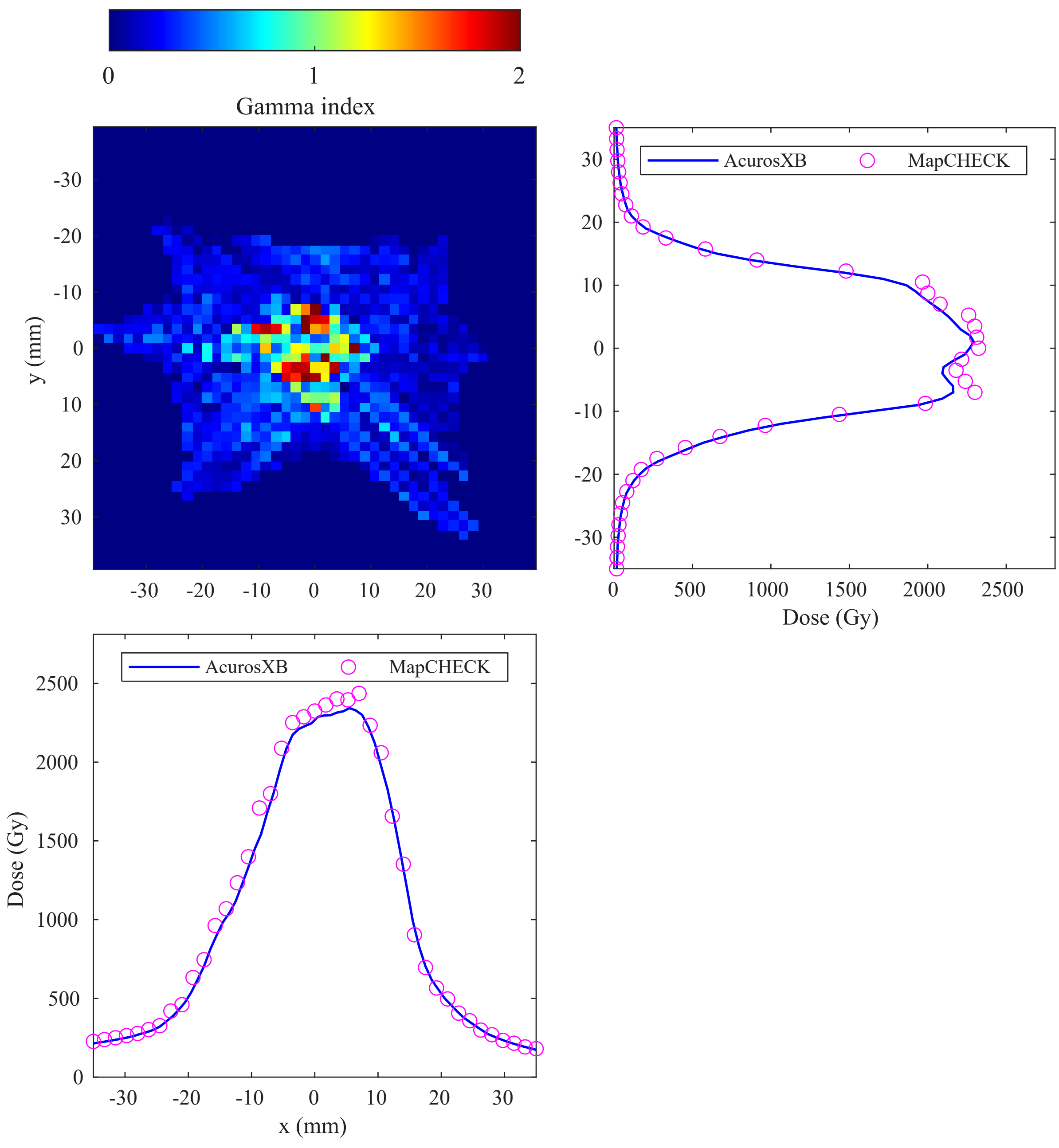

Figure S77. Measurement 75. Plan 50, 4 targets, equivalent diameter 11.8 mm. SRS MapCHECK fraction passing gamma 2%/1 mm = 100.0%

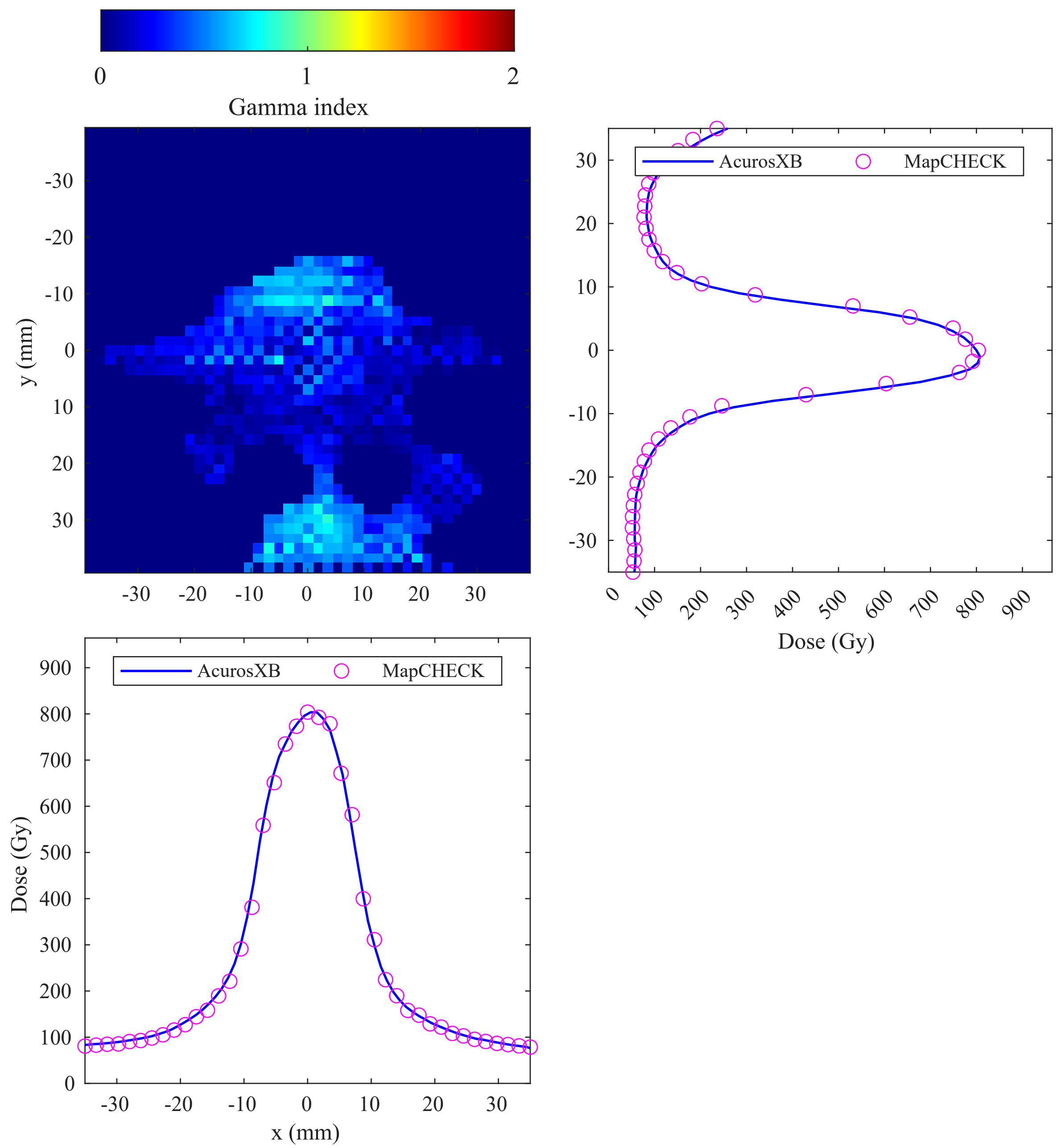

Figure S78. Measurement 76. Plan 50, 4 targets, equivalent diameter 8.3 mm. SRS MapCHECK fraction passing gamma 2%/1 mm = 96.4%

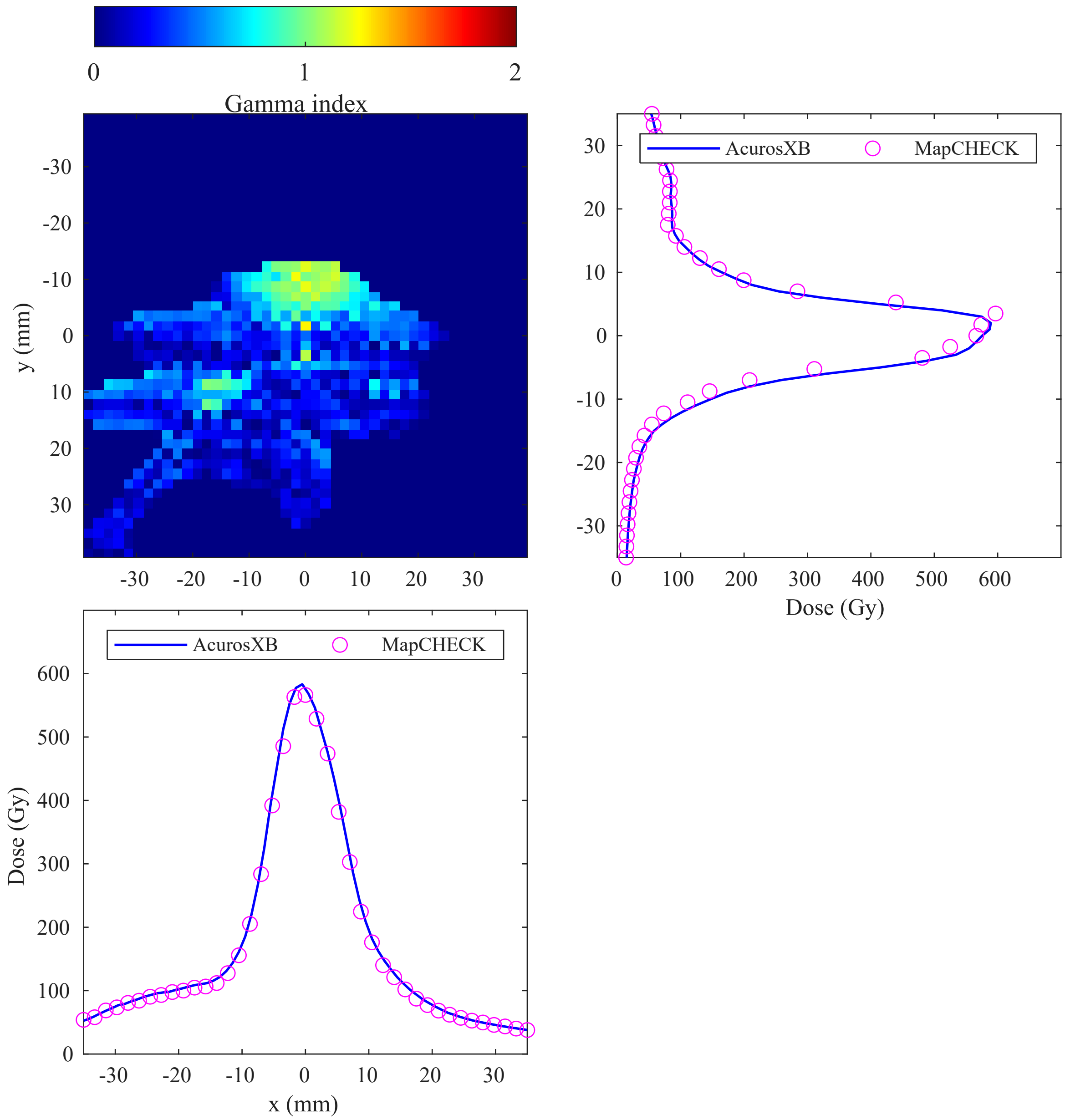

Figure S79. Measurement 77. Plan 51, 4 targets, equivalent diameter 6.8 mm. SRS MapCHECK fraction passing gamma 2%/1 mm = 99.5%

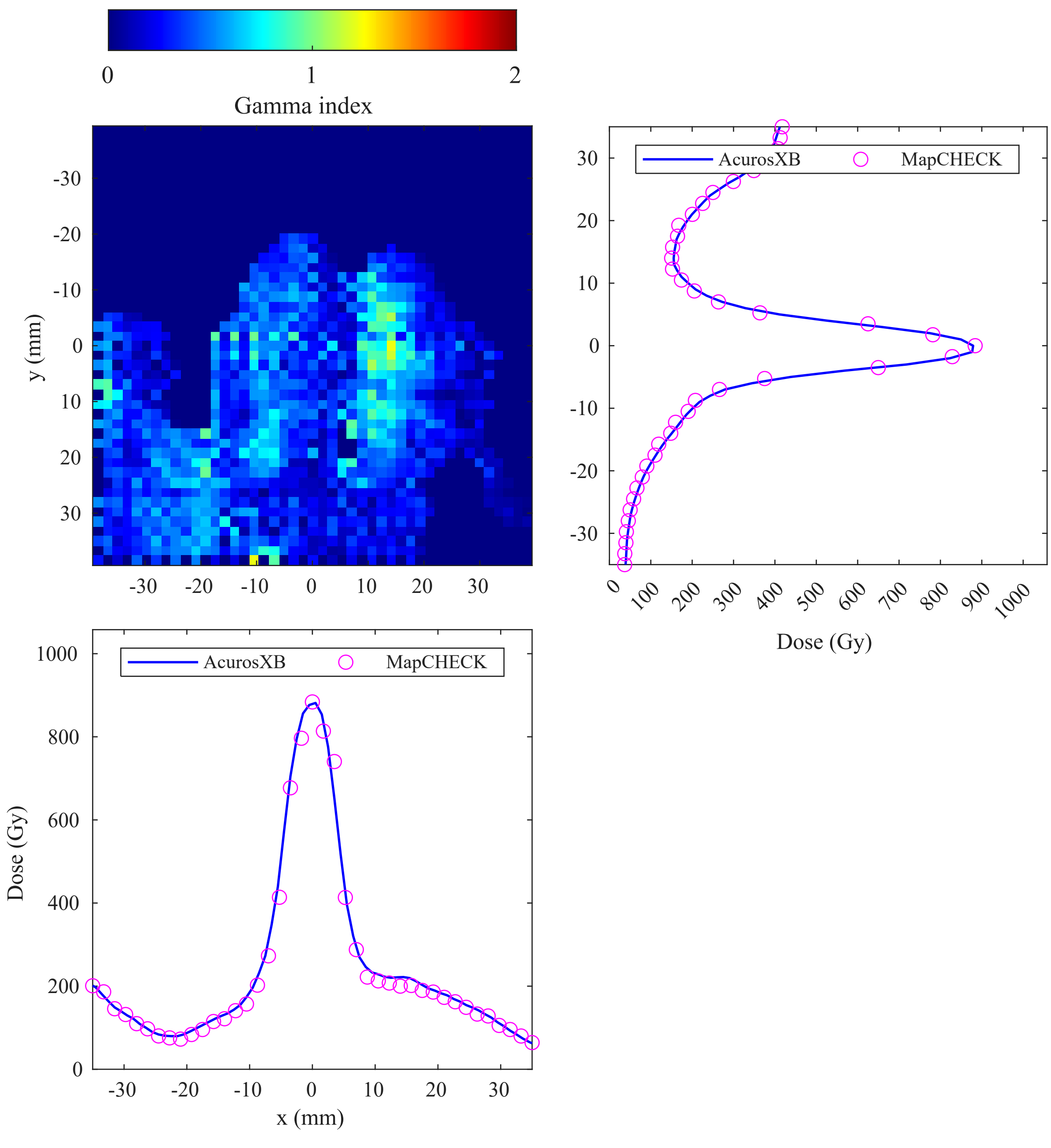

Figure S80. Measurement 78. Plan 51, 4 targets, equivalent diameter 16.2 mm. SRS MapCHECK fraction passing gamma 2%/1 mm = 98.8%

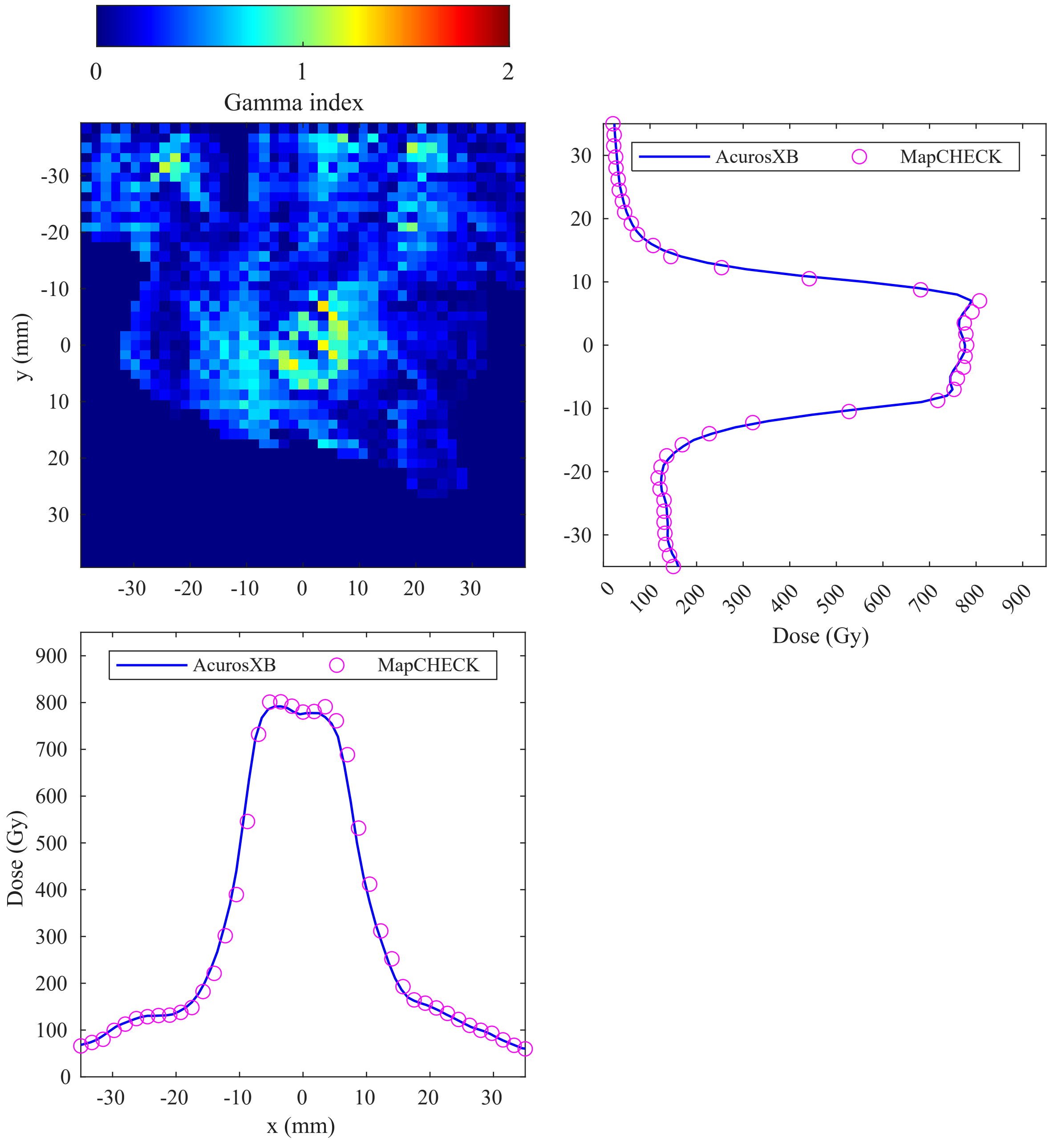

Figure S81. Measurement 79. Plan 52, single target, equivalent diameter 10.2 mm. SRS MapCHECK fraction passing gamma 2%/1 mm = 93.0%

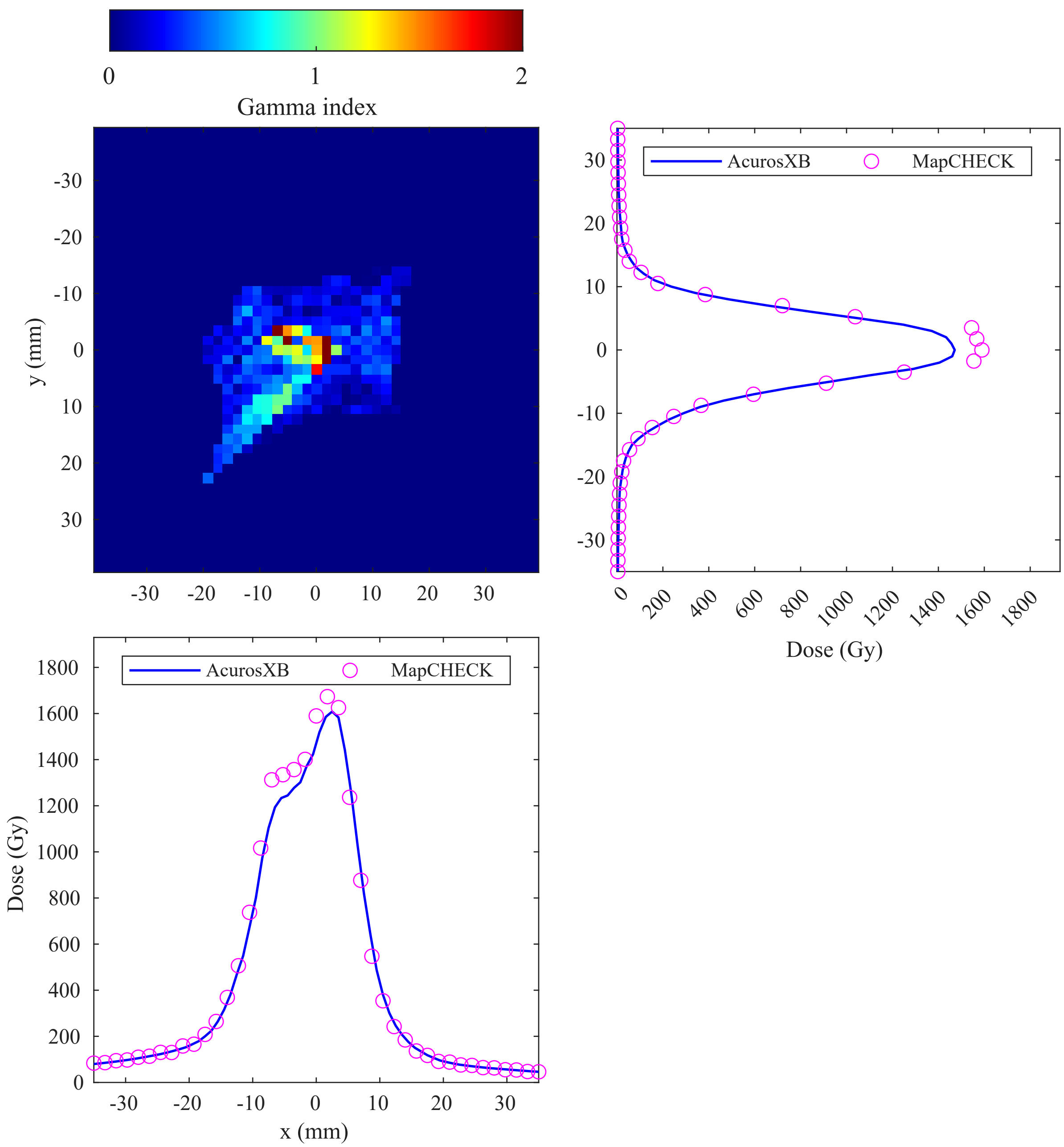

Figure S82. Measurement 80. Plan 53, 7 targets, equivalent diameter 20.4 mm. SRS MapCHECK fraction passing gamma 2%/1 mm = 99.1%

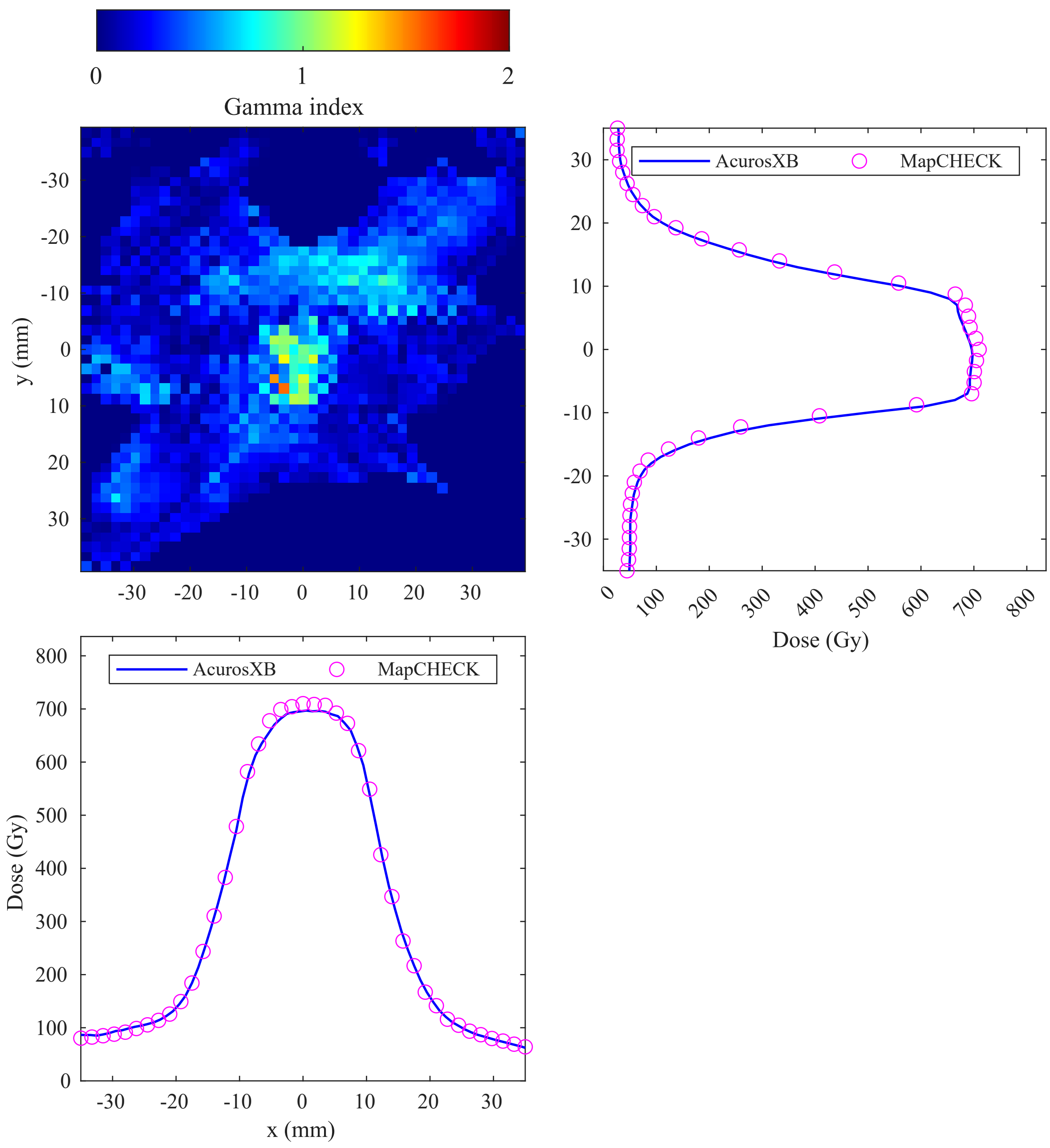

Figure S83. Measurement 81. Plan 53, 7 targets, equivalent diameter 3.2 mm. SRS MapCHECK fraction passing gamma 2%/1 mm = 98.9%

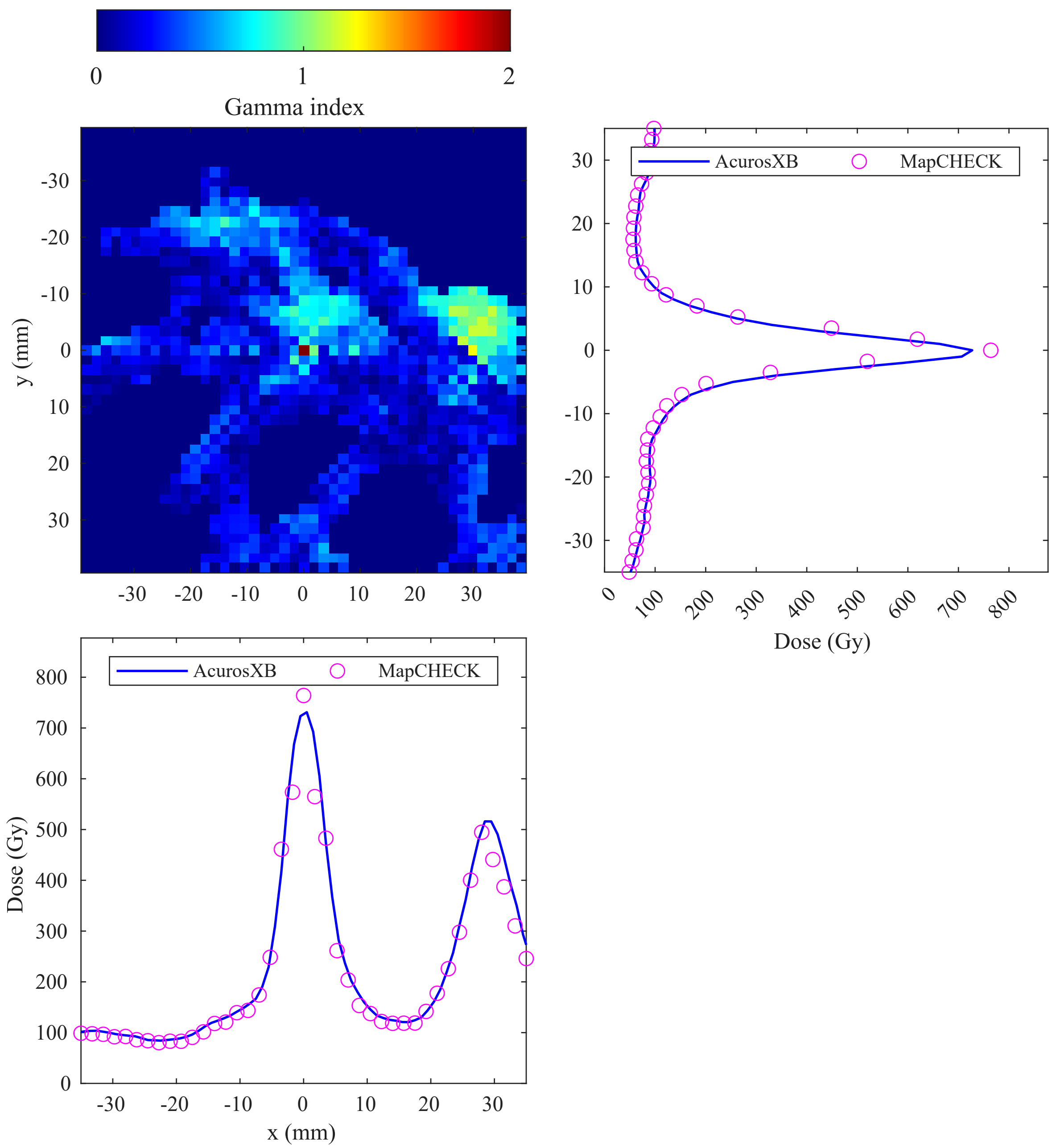

Figure S84. Measurement 82. Plan 54, 2 targets, equivalent diameter 6.6 mm. SRS MapCHECK fraction passing gamma 2%/1 mm = 99.5%

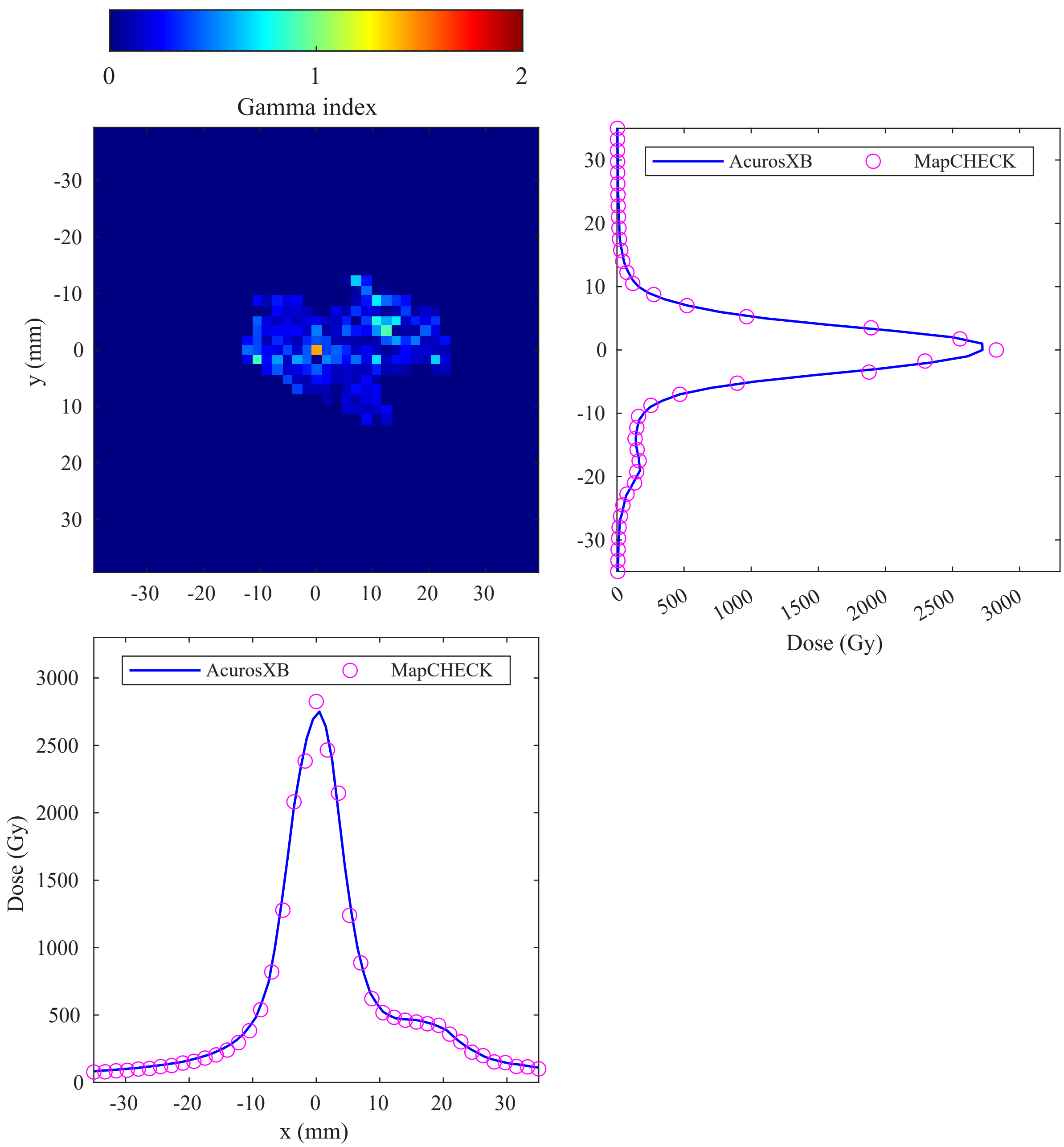

Figure S85. Measurement 83. Plan 54, 2 targets, equivalent diameter 3.8 mm. SRS MapCHECK fraction passing gamma 2%/1 mm = 97.7%

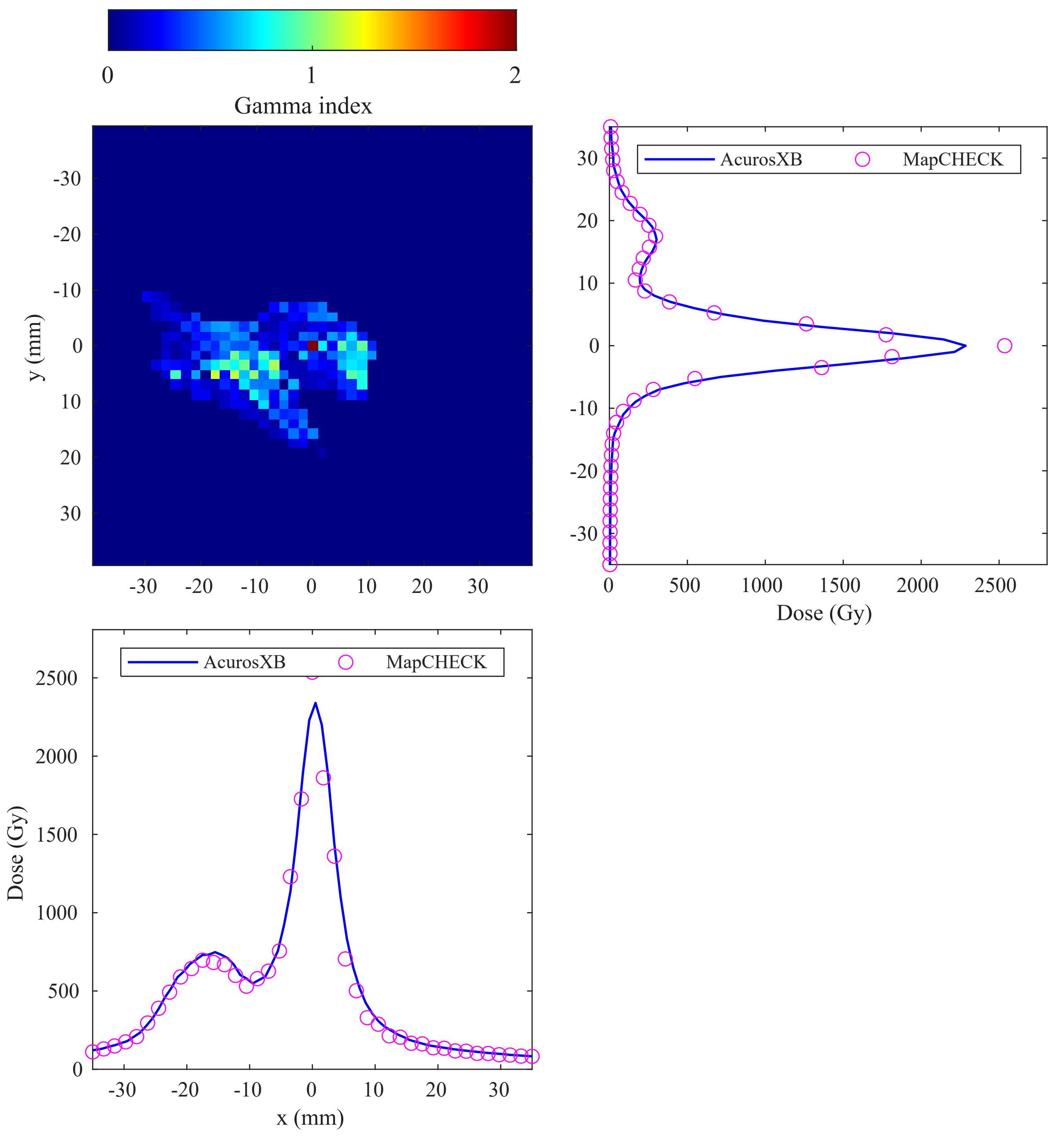

Figure S86. Measurement 84. Plan 55, single target, equivalent diameter 17.0 mm. SRS MapCHECK fraction passing gamma 2%/1 mm = 96.3%

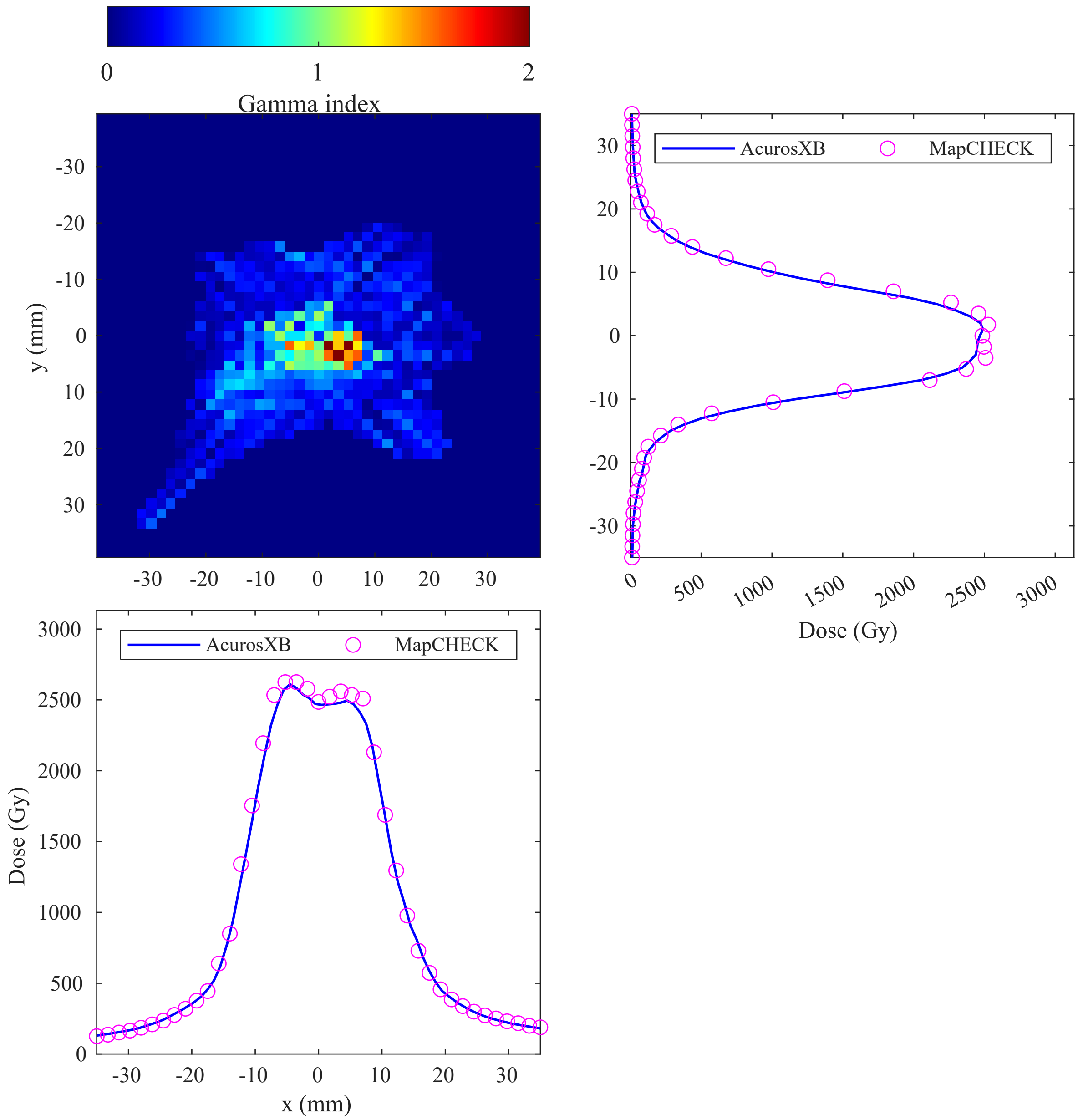

Figure S87. Measurement 85. Plan 56, single target, equivalent diameter 7.7 mm. SRS MapCHECK fraction passing gamma 2%/1 mm = 96.3%

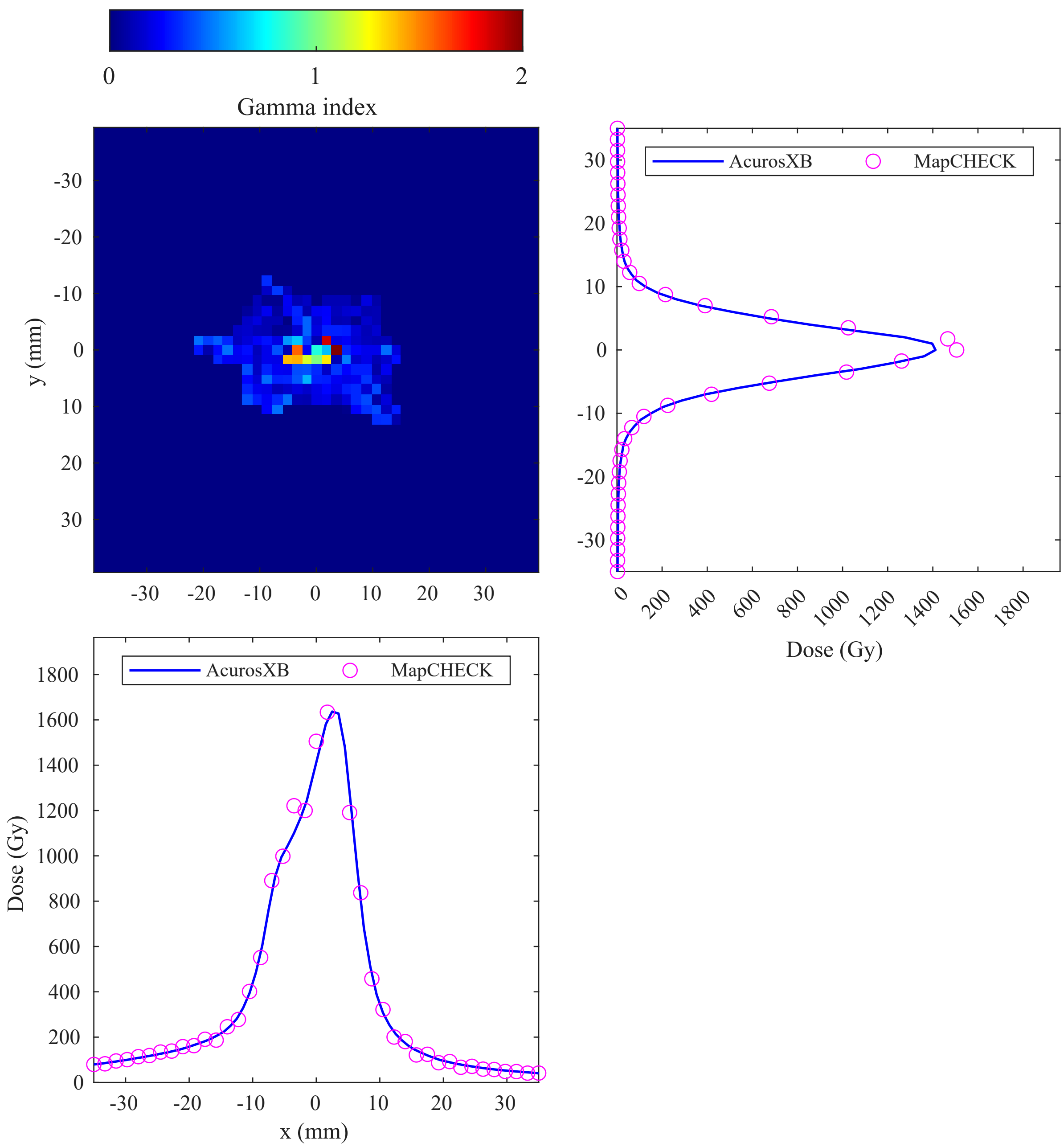

Figure S88. Measurement 86. Plan 57, single target, equivalent diameter 28.2 mm. SRS MapCHECK fraction passing gamma 2%/1 mm = 98.3%

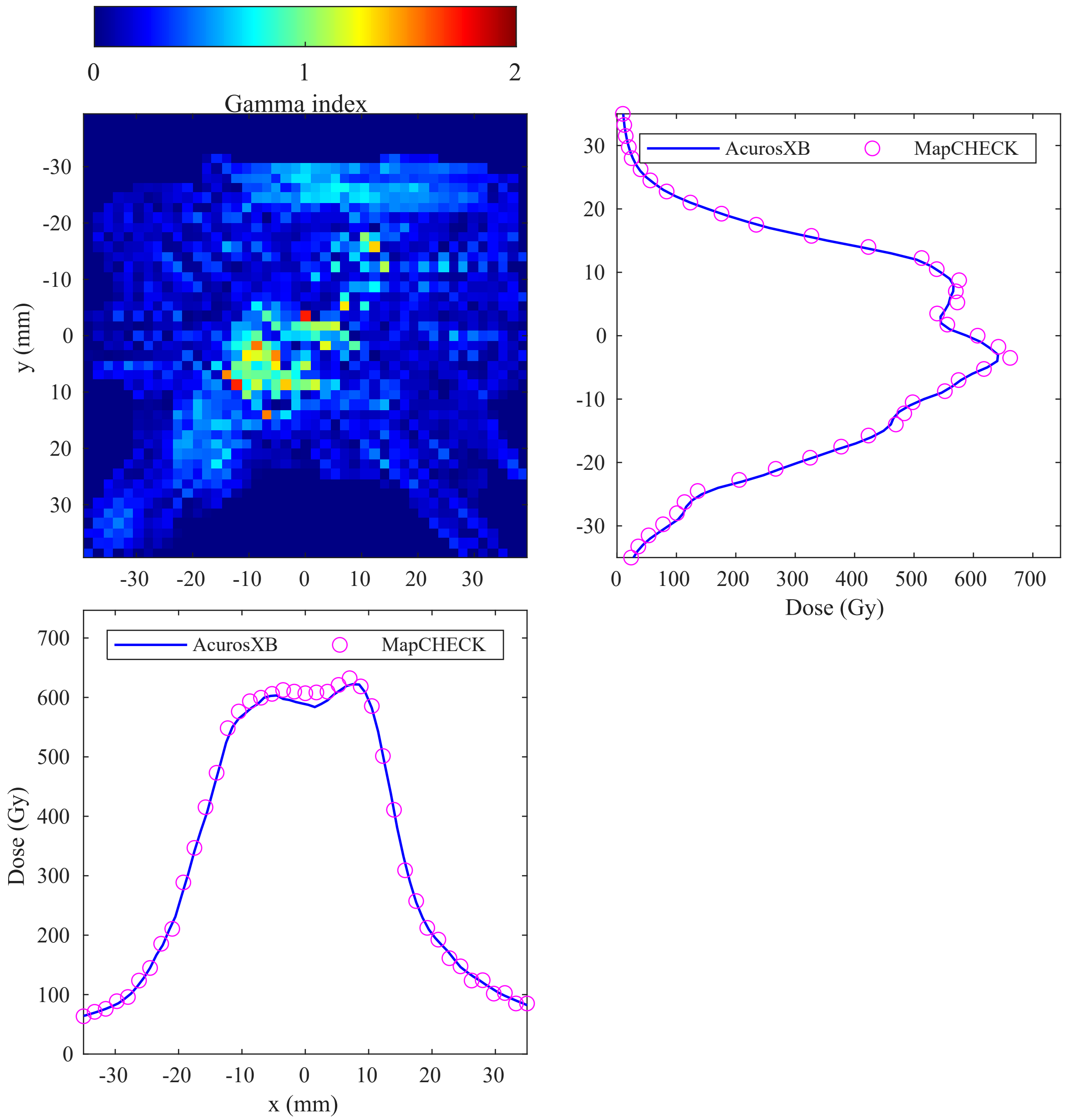

Figure S89. Measurement 87. Plan 58, single target, equivalent diameter 25.1 mm. SRS MapCHECK fraction passing gamma 2%/1 mm = 99.5%

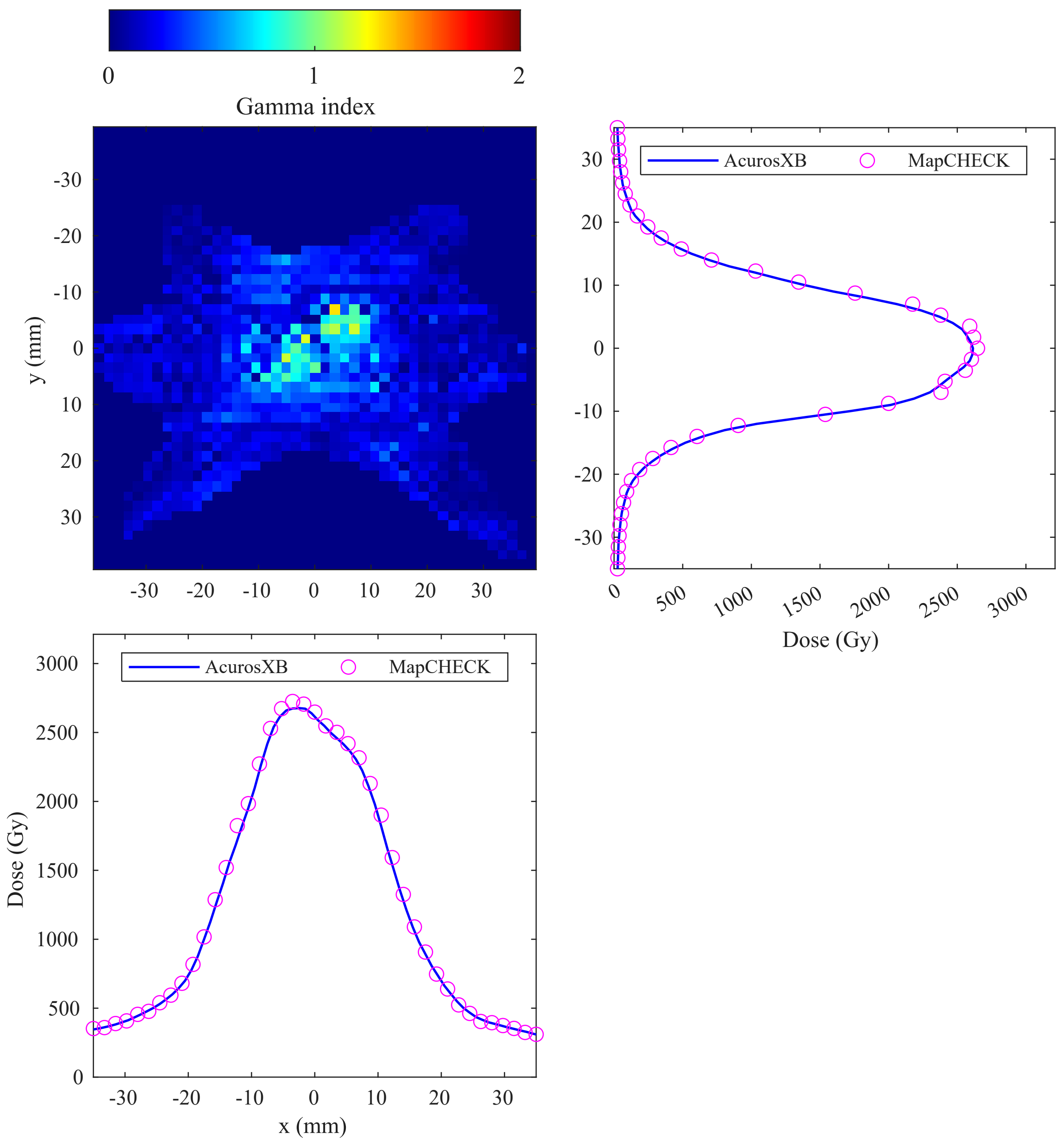

Figure S90. Measurement 88. Plan 59, single target, equivalent diameter 13.1 mm. SRS MapCHECK fraction passing gamma 2%/1 mm = 98.6%

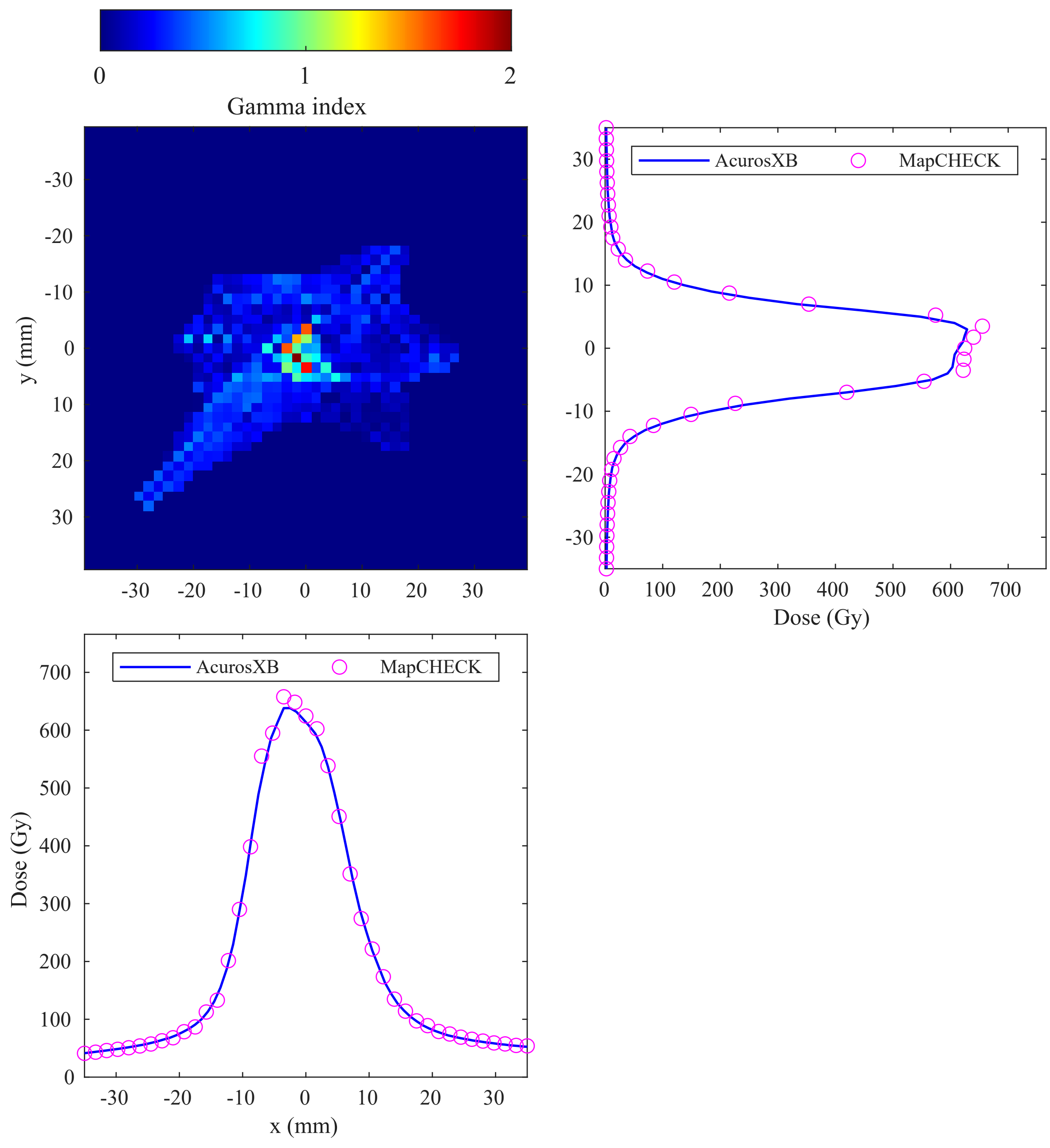

Figure S91. Measurement 89. Plan 60, 25 targets, equivalent diameter 18.2 mm. SRS MapCHECK fraction passing gamma 2%/1 mm = 97.8%

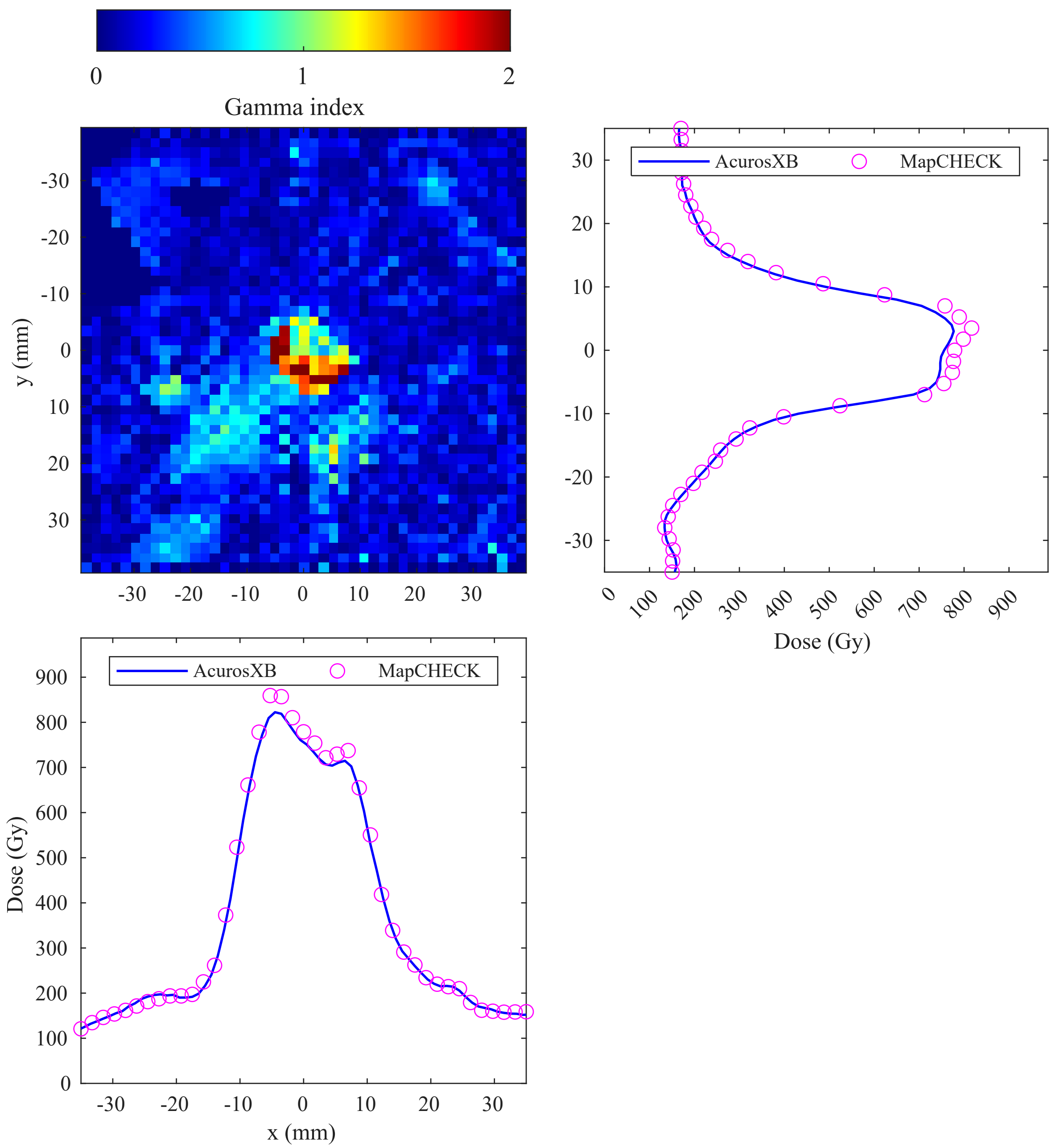

Figure S92. Measurement 90. Plan 60, 25 targets, equivalent diameter 3.5 mm. SRS MapCHECK fraction passing gamma 2%/1 mm = 98.3%

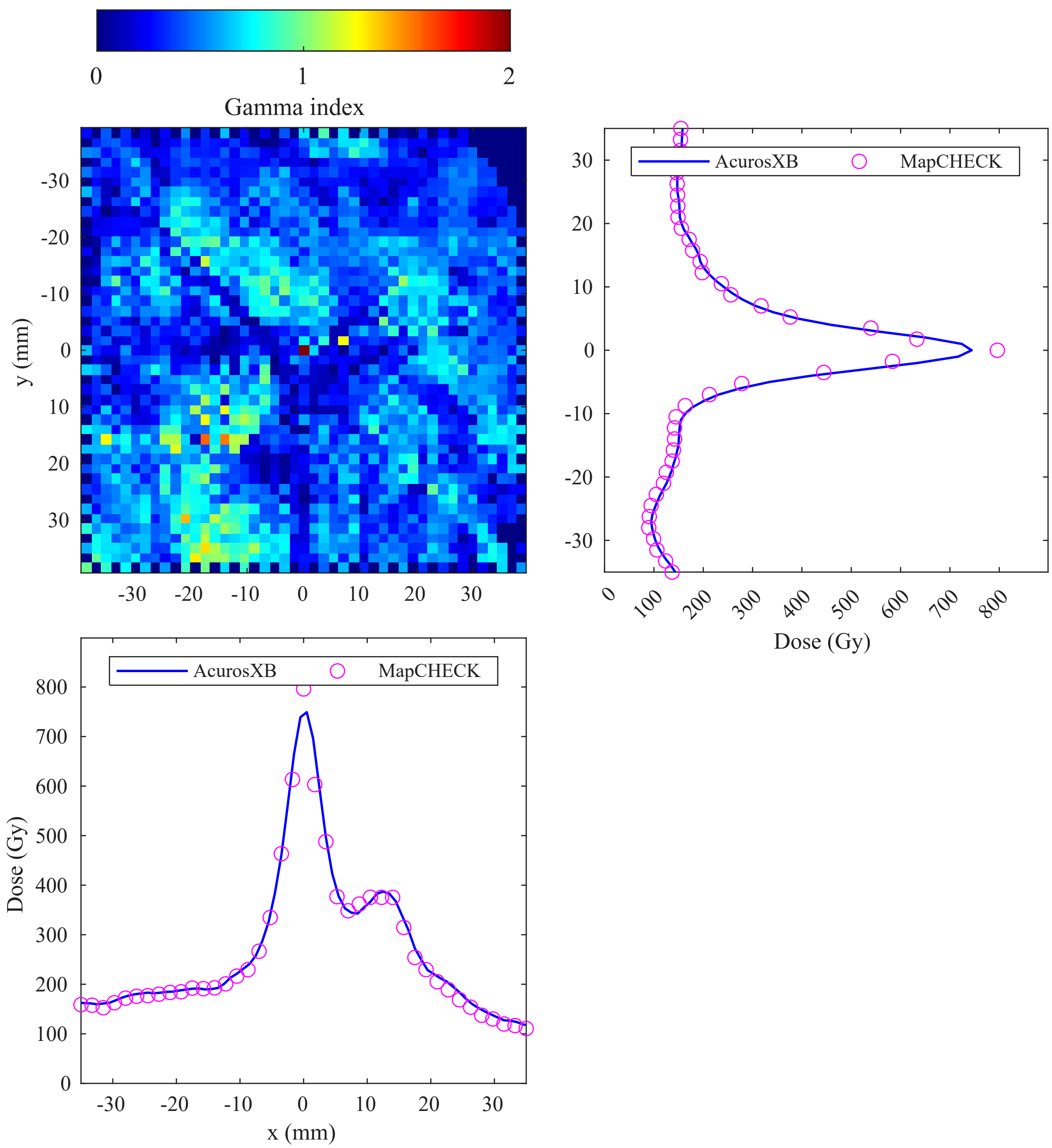

Supplement: Supplementary file 1 [file mmc1.pdf]
